# The Effect of Fiber Supplementation on Chronic Constipation in Adults: An Updated Systematic Review and Meta-Analysis of Randomized Controlled Trials

**DOI:** 10.1093/ajcn/nqac184

**Published:** 2022-07-11

**Authors:** Alice van der Schoot, Candice Drysdale, Kevin Whelan, Eirini Dimidi

**Affiliations:** Department of Nutritional Sciences, King's College London, London, United Kingdom; Department of Nutritional Sciences, King's College London, London, United Kingdom; Department of Nutritional Sciences, King's College London, London, United Kingdom; Department of Nutritional Sciences, King's College London, London, United Kingdom

**Keywords:** constipation, fiber, prebiotics, systematic review, meta-analysis

## Abstract

**Background:**

Chronic constipation is a prevalent disorder that remains challenging to treat. Studies suggest increasing fiber intake may improve symptoms, although recommendations on the fiber type, dose, and treatment duration are unclear.

**Objectives:**

We investigated the effects of fiber supplementation on stool output, gut transit time, symptoms, and quality of life in adults with chronic constipation via a systematic review and meta-analysis of randomized controlled trials (RCTs).

**Methods:**

Studies were identified using electronic databases, backward citation, and hand searches of abstracts. RCTs reporting administration of fiber supplementation in adults with chronic constipation were included. Risks of bias (RoB) was assessed with the Cochrane RoB 2.0 tool. Results were synthesized using risk ratios (RRs), mean differences, or standardized mean differences (SMDs) and 95% CIs using a random-effects model.

**Results:**

Sixteen RCTs with 1251 participants were included. Overall, 311 of 473 (66%) participants responded to fiber treatment and 134 of 329 (41%) responded to control treatment [RR: 1.48 (95% CI: 1.17, 1.88; *P* = 0.001); I^2^ = 57% (*P* = 0.007)], with psyllium and pectin having significant effects. A higher response to treatment was apparent in fiber groups compared to control groups irrespective of the treatment duration, but only with higher fiber doses (>10 g/d). Fiber increased stool frequency [SMD: 0.72 (95% CI: 0.36, 1.08; *P* = 0.0001); I^2^ = 86% (*P* < 0.00001)]; psyllium and pectin had significant effects, and improvement was apparent only with higher fiber doses and greater treatment durations (≥4 weeks). Fiber improved stool consistency (SMD: 0.32; 95% CI: 0.18, 0.46; *P* < 0.0001), particularly with higher fiber doses. Flatulence was higher in fiber groups compared to control groups(SMD: 0.80; 95% CI: 0.47, 1.13; *P* < 0.00001).

**Conclusions:**

Fiber supplementation is effective at improving constipation. Particularly, psyllium, doses >10 g/d and treatment durations of at least 4 weeks appear optimal, though caution is needed when interpreting the results due to considerable heterogeneity. These findings provide promising evidence on the optimal type and regime of fiber supplementation, which could be used to standardize recommendations to patients. The protocol for this review is registered at PROSPERO as CRD42020191404.

## Introduction

Chronic constipation is a common gastrointestinal disorder with an estimated prevalence of 12% in adults (1). It is characterized by predominant symptoms of infrequent, difficult, or incomplete defecation (2). Chronic constipation impacts quality of life, with impairments in social functioning and mental health (3). In the United States, each year there are more than 2.5 million visits to medical centers where constipation is the leading diagnosis, with annual direct costs of the condition ranging from $1912 to $11,991 per patient (4–6).

The management of chronic constipation can be challenging. Recommendations include lifestyle advice (e.g., increasing dietary fiber), followed by laxatives (7). However, half of people with constipation are dissatisfied with the current treatment options, primarily because of a lack of effectiveness and side effects (8). This highlights the need for better access to management strategies that can safely and effectively improve constipation symptoms.

Fiber supplementation is indicated as first-line management for chronic constipation in British, American, and European guidelines (7, 9–11). Fiber encompasses all carbohydrates that are neither digested nor absorbed in the small intestine and have a degree of polymerization of 3 or more monomeric units, plus lignin (12). This includes prebiotic fibers, which are substrates that are selectively utilized by host microorganisms, conferring a health benefit (13). Soluble, viscous fibers can influence stool bulking directly through water retention in the colon, resulting in softer stools. Insoluble, nonviscous fibers can cause mechanical stimulation of the gut mucosa that accelerates gut transit time (GTT) (14–16). Fermentable fibers may increase stool bulk indirectly through fermentation byproducts that influence contractile activity in the gut and increase microbial biomass through changes in the gut microbiome (14, 17). However, fermentation also results in the production of gas, which can cause side effects, such as flatulence or bloating.

A meta-analysis published in 2016 indicated beneficial effects of fiber supplementation at improving stool frequency and consistency in adults with chronic constipation, although its consumption led to side effects, such as flatulence (18). The review included only 7 randomized controlled trials (RCTs), limiting the interpretation of the overall effectiveness and subgroup analyses based upon the types of fiber, doses, and treatment durations, rendering its application to clinical practice challenging. Many studies have since been performed evaluating a range of types and doses of fiber and measuring additional outcomes. The aim of this systematic review and meta-analysis was to investigate the effects of fiber supplementation on response to treatment, stool output, GTT, symptoms, quality of life, adverse events, and compliance in adults with chronic constipation.

## Methods

This systematic review and meta-analysis was conducted following the guidelines of the Cochrane Handbook for Systematic Reviews of Interventions (19) and reported in accordance with the Preferred Reporting Items for Systematic Reviews and Meta-Analyses (PRISMA) updated guidelines (20). The eligibility criteria, search strategy, and methods of screening, data extraction, and data analysis were specified in advance and detailed in a protocol published on PROSPERO (CRD42020191404).

### Eligibility criteria

The eligibility criteria were developed using a Patient, Intervention, Comparators, Outcome, and Study Design (PICOS) approach and are outlined in **Table 1**. Briefly, the inclusion criteria were any RCTs reporting the effects of fiber supplementation in adults with chronic constipation that measured constipation outcomes.

**TABLE 1:** Table of inclusion and exclusion criteria and data extracted for participants, interventions, comparators, outcomes, and study designs (PICOS)

Characteristic	Inclusion and exclusion criteria	Data extracted
Participants	Adults (aged ≥ 18 years) of any sex or ethnicity with chronic idiopathic constipation identified through: *1*) clinical diagnostic criteria (e.g., Rome criteria); *2*) author-defined or clinician-defined criteria/diagnosis; *3*) participant-defined criteria (e.g., self-reported constipation); or *4*) the presence of ≥1 of the following symptoms indicative of constipation: <3 bowel movements per week, hard or lumpy stools, a sensation of incomplete evacuation, straining, manual maneuvers, physiological markers (e.g., slow gut transit time), or an evacuation disorder. Studies with inclusion criteria of ≥3 bowel movements per week and no other symptoms indicative of constipation were excluded. Community or outpatient settings were eligible. Studies were excluded if all participants had secondary constipation or belonged to specific clinical population groups (e.g., pregnant women, inpatients). However, a study was eligible if only a subset of the population had secondary constipation or belonged to 1 of these specific population groups.	Age, sex, and location participants recruited from; type of constipation and diagnosis method; inclusion and exclusion criteria; number of participants in groups; and number of participants with secondary constipation or from a specified population group (if present)
Intervention	Studies using supplementary fiber defined by the Scientific Advisory Committee on Nutrition (12). Individual- or mixed-fiber supplements, including prebiotic fibers, could be administered in the form of a pill, capsule, powder sachet, solution, or fortified food or drink (as long as the control group was such that the effect of the fiber alone could be isolated). Eligible dose was ≥3 g/d (Englyst method) or ≥4 g/d (Association of Analytical Chemists method) for a minimum of 2 weeks. Studies of fiber supplements in conjunction with other interventions (e.g., a dietary modification) were included if the effect of the fiber alone could be isolated. Studies based on dietary advice to increase fiber intake as an intervention were excluded.	Name of study product and ingredients; fiber type, degree of polymerization, form and dose; and schedule and duration of intervention
Comparators	Studies using an appropriate placebo as a control that allowed the effect of the fiber alone to be isolated. Where the fiber intervention was a fortified food or drink, an appropriate comparator was the same food or drink without the fiber. Studies that contained multiple study arms were included if the fiber and control arms could be isolated. Control interventions could contain a negligible amount of fiber (<0.5 g/d using the Englyst method or <0.67 g/d using the Association of Analytical Chemists method).	Type, form, dose, schedule, and duration of comparator
Outcomes	Studies reporting either dichotomous or continuous data on treatment success; stool frequency, consistency, and weight; whole and regional gut transit time; gastrointestinal symptoms (integrative symptom scores, frequency and severity of individual symptoms); quality of life; laxative use; adverse events; and compliance.	Outcomes; method of measurement; baseline, midpoint and endpoint values or change from baseline; and details of adverse events and compliance
Study design	Randomized controlled trials with ≥2 study groups where it was possible to extract data on the fiber and control interventions. Parallel-group and cross-over studies with a washout period of ≥2 weeks were eligible. Cross-over studies without an adequate washout period were only considered eligible if the data from the first period could be extracted to reduce the risk of a carryover effect.	Study design; washout period duration; intention-to-treat analysis; number of excluded participants and reasons for exclusion; number of centers; randomization method, allocation concealment, and blinding; and funding source, funder involvement, and conflicts of interest

### Search strategy

Studies were identified through a systematic search of electronic databases and a clinical trials register, a hand search of conference abstracts, and a back search of reference lists of eligible studies and relevant review papers.

The following databases were searched for eligible studies: MEDLINE (1946 to March 2022; OvidSP), EMBASE (1974 to March 2022; OvidSP), Web of Science (1900 to March 2022; Web of Knowledge portal) and the Cochrane Central Register of Controlled Trials (all years; The Cochrane Library). The final search date was 18 March 2022. Combinations of terms related to constipation and dietary fiber were used in searches as medical subject headings and free-text terms. Detailed search strategies are presented in **Supplemental Methods 1**. No restrictions were applied to language or publication date, and foreign-language articles were translated by native speakers. The US National Institute of Health clinical trials register (www.clinicaltrials.gov/) was searched in March 2022 to identify unpublished trials.

Abstracts from the following annual conferences were hand-searched: the American Society for Parenteral and Enteral Nutrition (2004 to 2022, *J Parent Enteral Nutr*), the American Society of Colon and Rectal Surgeons (2004 to 2021, *Dis Colon Rectum*), the Association of Coloproctology of Great Britain and Ireland (2004 to 2021, *Colorectal Dis*), the British Association for Parenteral and Enteral Nutrition (2004 to 2021, *Proc Nutr Soc, e-SPEN*), the British Dietetic Association (2004 to 2020, *J Hum Nutr Diet*), the British Society of Gastroenterology (2004 to 2021, *Gut*), Digestive Disease Week (2004 to 2021, *Gastroenterology*), and the European Society for Clinical Nutrition and Metabolism (2004 to 2021, *Clin Nutr, Clin Nutr Supp, e-SPEN*).

### Selection process

References were imported into a reference manager for assessment of eligibility (EndNote X9; Thomson Reuters). Following automatic and manual removal of duplicates, 2 reviewers (AvdS, CD) independently screened the titles and abstracts and then the full-text articles against the predefined inclusion and exclusion criteria. Disagreements were resolved by a third reviewer (ED).

### Data collection process

Two reviewers (AvdS, CD) independently extracted data from eligible studies onto a standardized form. The data extracted included the characteristics of the trial participants, the intervention, the comparator group, the outcomes measured, and the study design (Table 1). Extracted data were compared and discrepancies were resolved. Where a full paper, abstract, or trial registration provided insufficient data, the authors were contacted to provide additional information. When trial reporting allowed, data were extracted as intention-to-treat analyses. For dichotomous data, dropouts were assumed to be treatment failures. If this information was not clear in the paper, an analysis on all participants with reported evaluable data was undertaken. Where a study reported multiple measurement methods for an outcome [e.g., Patient Assessment of Constipation Symptoms (PAC-SYM) and Cleveland Clinic Constipation Score (CCCS) (21,22)], the data for the most frequently reported method across studies were selected for the meta-analysis. When necessary, data were estimated from figures using Plot Digitizer Version 2.6.9, as recommended by the Cochrane Handbook (19).

### Risk of bias assessment

Risks of bias (RoB) for eligible studies was assessed independently by 2 reviewers (AvdS, CD) (23). The Cochrane RoB 2.0 tool assesses 5 domains consisting of bias arising from: randomization; deviations from intended interventions; missing outcome data; outcome measurements; and selection of the reported results. Judgements were categorized as having a low risk, some concerns, or a high risk, and reasoning for each judgement was recorded. The highest level of bias across the individual domains determined the overall RoB for each outcome of each included study. Disagreements were resolved through discussion with a third reviewer (ED). Information in available clinical trial registrations and protocols was compared against each final publication to ensure that prespecified outcomes matched those in the final publication.

### Data synthesis

A meta-analysis was performed when data for the same outcome from 2 or more studies were obtained, using Review Manager Version 5.4 (The Cochrane Collaboration, 2020). When a study reported multiple timepoints, endpoint data were used for the meta-analysis. Dichotomous outcomes were expressed as risk ratios (RRs) and 95% CIs. Mean differences (MDs) were calculated for continuous outcomes that were measured using the same tool and reported in the same units, or where the same unit could be calculated through direct conversion (e.g., bowel movements per day converted to per week). Standardized mean differences (SMDs) were calculated for continuous outcomes that were measured or reported differently. Means and SDs, sample sizes, and *P* values were used for the analysis. Where medians and ranges were reported, means and SDs were estimated using guidance from Wan et al. (24). For studies that had multiple intervention arms administering different fiber doses, each dose was compared to the control separately (21, 22, 25), and the sample size of the control group was divided by the number of intervention arms to reduce the unit-of-analysis error (19).

Meta-analyses were performed using a random-effects model. Statistical heterogeneity was assessed using the χ^2^ test and quantified using the I^2^ statistic. Thresholds of 50% and 75% were considered to represent substantial and considerable heterogeneity, respectively. Subgroup analyses were performed to investigate heterogeneity and explore the effects of fiber type, prebiotic status, treatment dose, and treatment duration. For subgroup analyses, a *P* value < 0.1 was considered to be statistically significant (26). To assess for publication bias, funnel plots were generated where a meta-analysis included ≥10 studies and symmetry was identified by visual inspection (19).

## Results

**FIGURE 1:**
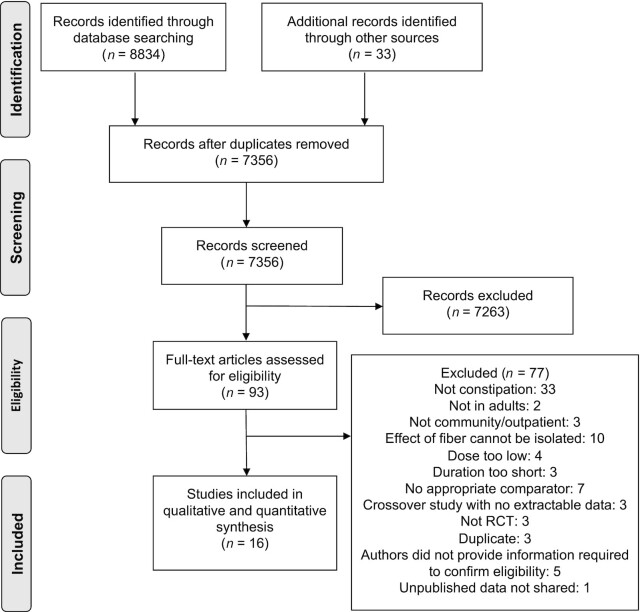
PRISMA flow diagram of studies included in the systematic review. PRISMA, Preferred Reporting Items for Systematic Reviews and Meta-Analyses; RCT, randomized controlled trial.

A total of 7356 nonduplicated records were identified, of which 93 were deemed to be potentially eligible (**Figure 1**). Of these, 77 records were excluded (**Supplemental Table 1**). One completed but unpublished trial (NCT01847950) was confirmed to be eligible after contact with the principal investigator; however, the data could not be shared due to a confidentiality agreement (27). In total, 16 RCTs fulfilled the criteria for inclusion in the review, involving 1251 participants with chronic constipation (21, 22, 25, 28–40). **Table 2** displays the characteristics of the included studies. Twelve studies were in English, 2 were in Spanish (29, 35), 1 was in Korean (40), and 1 was in Chinese (38). There were 14 parallel RCTs and 2 crossover RCTs. For 1 crossover study, data from the first period only were extracted due to an inadequate washout period (39). There was variability in the fiber type (e.g., psyllium, polydextrose), dose (4 to 40 g/d), and treatment duration (2 to 8 weeks) studied. Authors of all studies were contacted for further information. Of these, 7 replied (21, 22, 25, 31, 32, 34, 36) and 1 provided data for inclusion in the analyses (21).

**TABLE 2:** Characteristics of randomized controlled trials investigating the effect of fiber supplementation on chronic constipation in adults^1^

Study, year (ref)	Study design	Sample size (% female)	Age, years, mean ± SD (range)^2^	Constipation diagnosis	Fiber type and form	Daily dose, duration	Comparator, form	Outcomes included in meta-analysis
Duncan et al., 2018 (21)	Double blind, parallel	119 (93%)	High dose: 46.8 ± 15.1 (19–64); low dose: 29.2 ± 15.7 (19–72); control: 30.9 ± 16.0 (19–71)	Modified Rome III criteria and CCCS score of 8–20	Polydextrose powder, mixed in 200 ml water (nonprebiotic)	High dose: 12 g/d; low dose: 8 g/d, 4 weeks	Maltodextrin, mixed in 200 ml water	Stool frequency, stool consistency, GTT, symptoms, QoL
Ibarra et al., 2019 (25)	Double blind, parallel	192 (69%)	42.7 ± 18.8 (NR)	Rome III criteria	Polydextrose powder, mixed in 250 ml water (nonprebiotic)	High dose: 12 g/d; medium dose: 8 g/d; low dose: 4 g/d, 2 weeks	Maltodextrin, mixed in 250 ml water	Stool frequency, stool consistency, GTT, symptoms, QoL, response (constipation relief)
Ashraf et al., 1995 (28)	Double blind, parallel	22 (64%)	51, NR (35–70)	≤3 bowel movements/week, confirmed by prospectively administered stool diaries	Psyllium powder, mixed in 230ml water (nonprebiotic)	10.2 g/d, 8 weeks	Maltodextrin and excipients in 230 ml water	Stool frequency, stool consistency, stool weight, GTT, symptoms
Tomás-Ridocci et al., 1992 (29)	Double blind, parallel	20 (NR)	36 ± 14 (NR)	<3 bowel movements/week and/or hard stools that were difficult/painful to pass. IBS-C diagnosis based upon clinical symptomatology, absence of pathologies	Psyllium powder (nonprebiotic)	20 g/d, 1 month	Coated food paste	Stool frequency, stool consistency, stool weight, GTT, response (global symptom improvement)
Fenn et al., 1986 (30)	Single blind, parallel	201 (75%)	49, NR (17–70)	Functional constipation “assessed clinically on entry”, definition NR	Psyllium powder (nonprebiotic)	10.8 g/d, 2 weeks	Powder of sucrose, citric acid, other ingredients not disclosed	Stool frequency, stool consistency, symptoms, response (global symptom improvement)
Yang et al., 2021 (31)	Single blind, parallel	54 (100%)	Fiber: 31.2 ± 6.3; control: 34.1 ± 6.2 (18–49)	Rome IV criteria	Psyllium powder mixed in warm water (nonprebiotic)	40 g/d, 4 weeks	Components of psyllium without the seed husk	Response (no straining)
Glibowski et al., 2020 (32)	NR, parallel	20 (NR)	NR (20–29)	≤2 bowel movements/week, and/or hard and dry stools that are difficult to pass, and/or feeling of incomplete evacuation	Inulin-enriched apple juice, 300 ml (prebiotic)	12 g/d, 2 weeks	Apple juice without fiber supplement, 300 ml	Stool frequency
Marteau et al., 2011 (33)	Double blind, parallel	50 (88%)	57, NR (50–70)	<3 bowel movements/week and/or straining in defecation	Inulin powder (prebiotic)	15 g/d, 4 weeks	Maltodextrin	Stool consistency, symptoms
Micka et al., 2017 (34)	Double blind, cross-over	54 (75%)	46.9 (NR)	2–3 bowel movements/week for at least 6 months	Inulin powder, mixed in 200 ml hot drink (prebiotic)	12 g/d, 4 weeks	Maltodextrin, mixed in 200 ml hot drink	Stool frequency, stool consistency, QoL (physical discomfort and satisfaction subscales), symptoms
López Román et al., 2008 (35)	Double blind, parallel	32 (88%)	46.9 ± 13.2 (NR)	Rome II criteria	Inulin and resistant maltodextrin-enriched milk, 0.5 L/d (mixture of prebiotic and nonprebiotic)	22.5 g/d, 20 days	Milk without fiber supplement, 0.5 L/d	Stool frequency, stool consistency, response (no straining)
Waitzberg et al., 2012 (36)	Double blind, parallel	60 (100%)	Fiber: 36.1, NR (NR); control: 40.2, NR (NR)	<3 bowel movements/week for at least 3 months	Inulin and partially hydrolyzed guar gum powder; (mixture of prebiotic and nonprebiotic)	15 g/d, 3 weeks	Maltodextrin	Stool frequency, response (constipation relief)
Li et al., 2017 (37)	Double blind, parallel	103 (69%)	NR (NR)	Decreased stool frequency, hardened stool consistency, or <3 bowel movements/week	α-Galacto-oligosaccharide powder, mixed in water (prebiotic)	5 g/d, 30 days	Maltodextrin, mixed in water	Stool frequency, stool consistency
Schoemaker et al., 2022 (22)	Double blind, parallel	132 (94%)	High dose: 37.3 ± 11.5; low dose: 38.9 ± 11.8; control: 38.8 ± 13.3, (18–56)	Rome IV criteria	Galacto-oligosaccharide powder mixed in drink (prebiotic)	High dose: 11 g/d; low dose: 5.5 g/d, 3 weeks	Maltodextrin, mixed in drink	Stool frequency, stool consistency, symptoms, response (increase of ≥1 bowel movement/week)
Xu et al., 2014 (38)	Double blind, parallel	80 (61%)	44.7 ± 13.4 (NR)	Rome III criteria and slow-transit constipation	Pectin powder, before meal (nonprebiotic)	24 g/d, 4 weeks	Maltodextrin before meal	Stool frequency, GTT, symptoms, response (effective symptom improvement)
Badiali et al., 1995 (39)	Double blind, cross-over	29 (89%)	37, NR (20–65)	Slow large-bowel transit and seeking medical advice for chronic primary constipation	Wheat bran powder, mixed in water (nonprebiotic)	12.5 g/d, 4 weeks	Saccharose, cocoa powder, maltose in water	Stool frequency, stool weight, GTT, symptoms, response (no straining)
Huh et al., 2007 (40)	Double blind, parallel	77 (100%)	Fiber: 31.2 ± 8.3; control: 32.2 ± 6.9 (NR)	Rome II criteria	Unspecified fiber-enriched yogurt, 150 ml	15 g/d, 4 weeks	Yogurt without fiber supplement, 150 ml	Stool frequency, GTT, symptoms

1 CCCS, Cleveland Clinic Constipation Score; GTT, gut transit time; IBS-C, irritable bowel syndrome with constipation; QoL, quality of life; NR, not reported; Ref, reference.

2 Values are for the whole study population unless groups are specified.

### Outcomes

The outcomes of the meta-analysis are reported in **Table 3**. Forest plots for subgroup analyses on fiber type, prebiotic status, doses, and treatment durations for each outcome are found in **Supplemental Figures 1–22**.

**TABLE 3:** Results of meta-analyses comparing fiber supplementation with control groups for response to treatment, stool output, gut transit time, symptoms and quality of life in adults with chronic constipation^1^

			Results		Heterogeneity		
Outcome	Number of studies in meta-analysis (refs)	Participants (*n*)	Meta-analysis overall estimate (95% CI)	*P * ^2^	χ^2^ test	*P*	I^2^ (%)
Response to treatment	9^3,4^ (22,25,29–31,35,36,38,39)	802	RR: 1.48 (1.17, 1.88)	0.001	25.87	0.007	57%
Stool output							
Stool frequency	14^3,5^ (21,22,25,28–30,32,34–40)	1040	SMD: 0.72 (0.36, 1.08)	0.0001	121.12	<0.00001	86%
Stool consistency	10^3,5^ (21,22,25,28–30,33–35,37)	918	SMD: 0.32 (0.18, 0.46)	<0.0001	12.73	0.47	0%
Stool weight	3 (28,29,39)	59	MD: 31.93 g/d (−3.74 g/d, 67.60 g/d)	0.08	3.73	0.15	46%
Gut transit time							
Whole GTT	7^3,4^ (21,25,28,29,38–40)	485	MD: −7.5 h (−18.1 h, 3.1 h)	0.17	47.55	<0.00001	81%
Right GTT	2^3^ (21,29)	125	MD: −1.4 h (−12.0 h, 9.3 h)	0.80	9.65	0.008	79%
Left GTT	2^3^ (21,29)	125	MD: 2.8 h (−6.5 h, 12.1 h)	0.56	6.76	0.03	70%
Rectosigmoid GTT	2^3^ (21,29)	125	MD: 3.6 h (−1.5 h, 8.8 h)	0.17	0.89	0.64	0%
Gastrointestinal symptoms							
Integrative symptom score	5^3,5^ (21,22,25,38,39)	531	SMD: −0.15 (−0.39, 0.08)	0.20	12.68	0.12	37%
PAC-SYM global	3^3,5^ (21,22,25)	428	MD: −0.10 (−0.31, 0.11)	0.35	11.49	0.07	48%
PAC-SYM abdominal	2^3,4^ (21,25)	296	MD: −0.03 (−0.26, 0.19)	0.77	6.03	0.20	34%
PAC-SYM rectal	2^3,4^ (21,25)	296	MD: −0.07 (−0.28, 0.14)	0.53	9.85	0.04	59%
PAC-SYM stool	2^3,4^ (21,25)	296	MD: −0.14 (−0.37, 0.10)	0.25	5.35	0.25	25%
Straining (severity)	4^3^ (25,28,30,34)	498	SMD: −0.32 (−0.59, −0.04)	0.02	9.27	0.10	46%
Incomplete evacuation (severity)	2 (28,34)	110	SMD: −0.02 (−0.39, 0.35)	0.93	0.00	0.96	0%
Bloating (severity)	4 (33,34,38,39)	239	SMD: 0.07 (−0.38, 0.51)	0.77	6.29	0.10	52%
Flatulence (severity)	3 (33,34,39)	153	SMD: 0.80 (0.47, 1.13)	<0.00001	0.35	0.84	0%
Abdominal pain/discomfort (severity)	3 (31,34,39)	299	SMD: −0.14 (−0.36, 0.09)	0.23	1.39	0.50	0%
Quality of life							
PAC-QoL global	2^3,4^ (21,25)	296	MD: −0.04 (−0.19, 0.10)	0.54	6.36	0.17	37%
PAC-QoL satisfaction	3^3,4^ (21,25,34)	384	MD: −0.05 (−0.32, 0.23)	0.74	12.15	0.03	59%
PAC-QoL physical discomfort	3^3,4^ (21,25,34)	384	MD: 0.12 (−0.08, 0.32)	0.25	6.77	0.24	26%
PAC-QoL psychosocial discomfort	2^3,4^ (21,25)	296	MD: −0.04 (−0.18, 0.11)	0.63	1.74	0.78	0%
PAC-QoL worries and concerns	2^3,4^ (21,25)	296	MD: −0.04 (−0.21, 0.12)	0.60	3.69	0.45	0%

1 Data were meta-analyzed using a random effects model. Statistical heterogeneity was assessed using the χ^2^ test and quantified using the I^2^ statistic. GTT, gut transit time; MD, mean difference; PAC-SYM, Patient Assessment of Constipation Symptoms; PAC-QoL, Patient Assessment of Constipation Quality of Life; Refs, references; RR, risk ratio; SMD, standardized mean difference.

2 *P* value is statistically significant at a value <0.05.

3 Analysis includes 1 study that has 3 separate entries for different doses (25).

4 Analysis includes 1 study that has 2 separate entries for different fiber doses (21).

5 Analysis includes 2 studies that have 2 separate entries for different fiber doses (21, 22).

### Response to treatment

**FIGURE 2:**
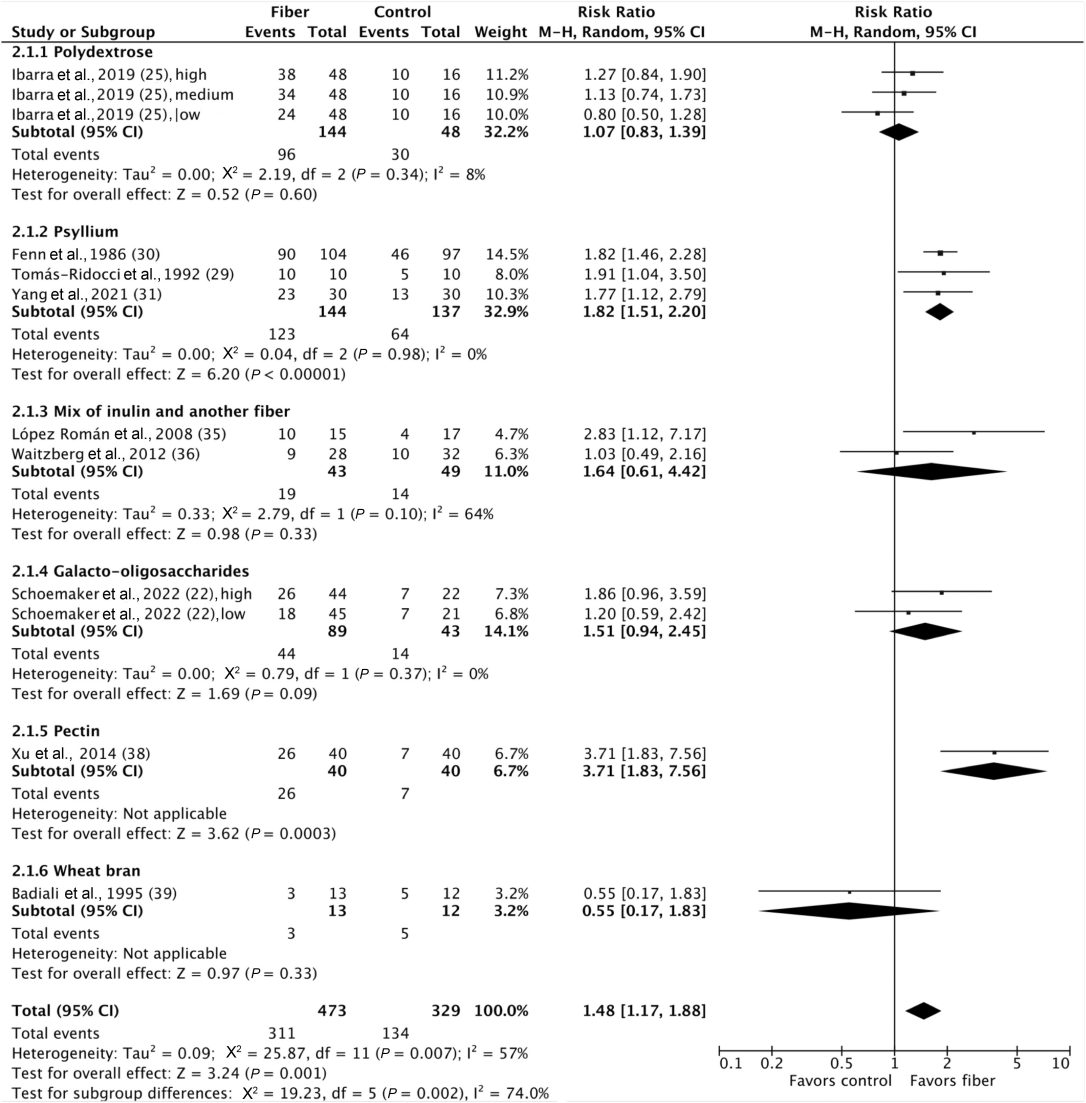
Forest plot of response to treatment in randomized controlled trials comparing fiber with control groups in adults with chronic constipation (*n* = 802). Values were calculated as RRs (95% CIs) using a random-effects model. M-H, Mantel-Haenszel; RR, risk ratio.

Response to treatment, measured as symptomatic improvement, was reported as dichotomous data in 9 studies including 802 participants (22, 25, 29–31, 35, 36, 38, 39). Overall, 311 of 473 (66%) participants responded to fiber treatment and 134 of 329 (41%) responded to control treatment (RR: 1.48; 95% CI: 1.17, 1.88; *P* = 0.001), with substantial heterogeneity between studies (I^2^ = 57%; *P* = 0.007). Subgroup analyses showed significantly greater responses to treatment in the 3 trials of psyllium supplementation, with no significant heterogeneity [RR: 1.82 (95% CI: 1.51, 2.20; *P* < 0.00001); I^2^ = 0% (*P* = 0.98)] and in 1 trial of pectin supplementation (RR: 3.71; 95% CI: 1.83, 7.56; *P* = 0.0003) (**Figure 2**). Nonprebiotics increased response to treatment [RR: 1.46 (95% CI: 1.08, 1.97; *P* = 0.01); I^2^ = 69% (*P* = 0.002)], while prebiotics and mixtures of prebiotics and nonprebiotics did not; however, no significant subgroup differences were detected (*P* = 0.97). A significant effect was shown for high fiber doses (>10 g/d) [RR: 1.72 (95% CI: 1.35, 2.18; *P* < 0.0001); I^2^ = 41% (*P* = 0.09)], but not low fiber doses (≤10 g/d), with a statistically significant subgroup effect (*P* = 0.005); however, only a low number of studies contributed data to the low-dose subgroup. All treatment durations (less than or greater than 4 weeks) were effective at increasing response to treatment (Supplemental Figure 1).

### Stool frequency

**FIGURE 3:**
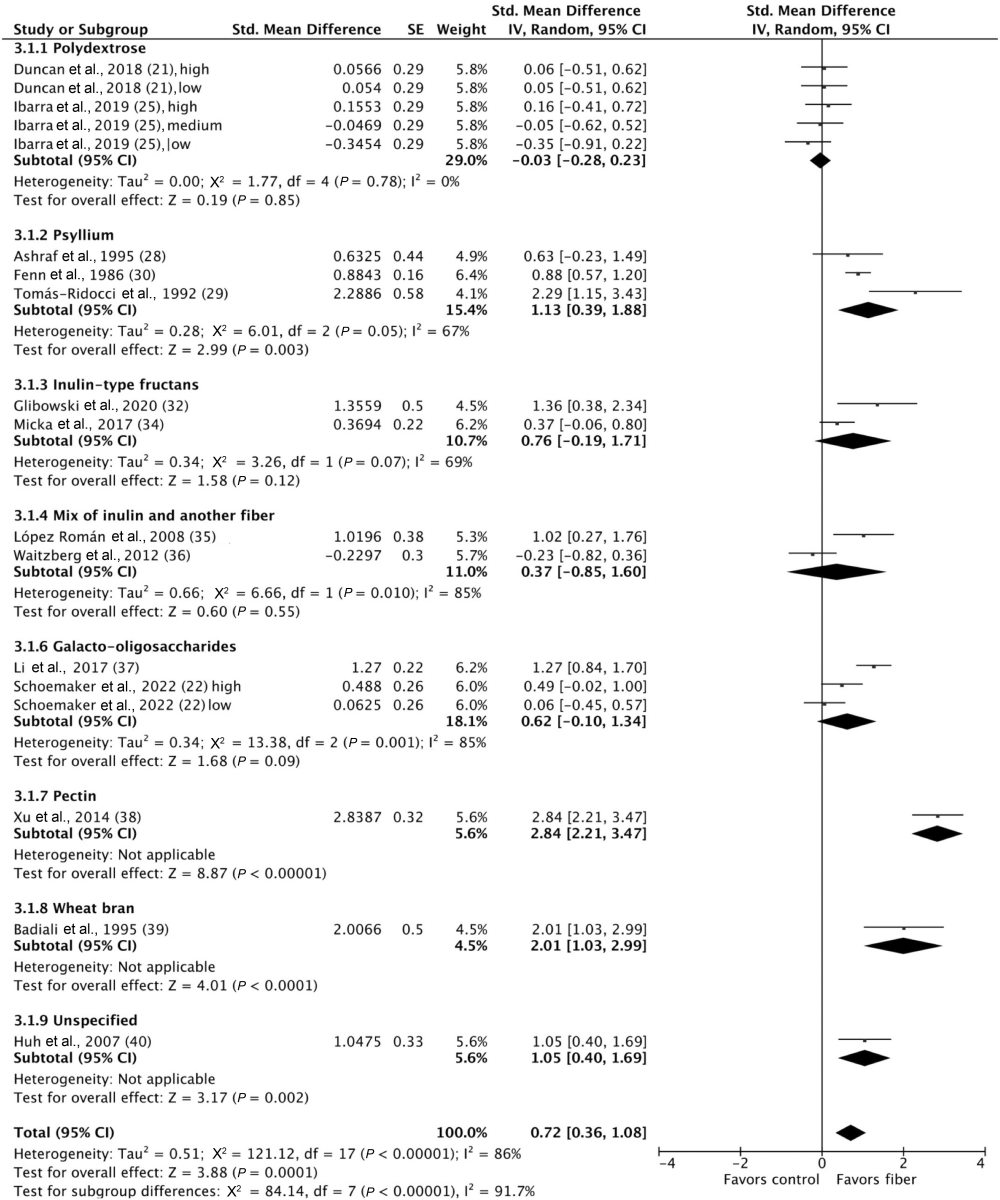
Forest plot of stool frequency in randomized controlled trials comparing fiber with control groups in adults with chronic constipation (*n* = 1040). Values were calculated as standardized mean differences (95% CIs) using a random-effects model. IV, inverse variance.

Stool frequency was measured in 15 studies; however, 1 study did not report data in a form that could be included in the meta-analysis, and these data were not obtained on request (33). Fourteen studies including 1040 participants were included in the meta-analysis (21, 22, 25, 28–30, 32, 34–40). Stool frequency was reported in various units, such as bowel movements/week and days between bowel movements. Fiber significantly increased stool frequency compared to control (SMD: 0.72; 95% CI: 0.36, 1.08; *P* = 0.0001); however, considerable heterogeneity was detected (I^2^ = 86%; *P* < 0.00001). Subgroup analyses showed that psyllium significantly increased stool frequency [SMD: 1.13 (95% CI: 0.39, 1.88; *P* = 0.003); I^2^ = 67% (*P* = 0.05)], as did pectin and wheat bran. However, polydextrose, inulin-type fructans, and galacto-oligosaccharides (GOS) did not impact stool frequency (**Figure 3**). Prebiotics and nonprebiotics were effective at increasing stool frequency when administered individually but not as mixtures; however, no significant subgroup differences were detected (*P* = 0.73). High fiber doses (>10 g/d) significantly increased stool frequency [SMD: 0.93 (95% CI: 0.49, 1.38; *P* < 0.0001); I^2^ = 86% (*P* < 0.00001)], but low fiber doses (≤10 g/d) did not, with a statistically significant subgroup effect (*P* = 0.06). Greater treatment durations (≥4 weeks) significantly increased stool frequency [SMD: 1.13 (95% CI: 0.50, 1.76; *P* = 0.0001); I^2^ = 89% (*P* < 0.00001)], but shorter durations (<4 weeks) did not, with a statistically significant subgroup effect (*P* = 0.03) (Supplemental Figure 2). Heterogeneity remained high between studies within each subgroup. No significant funnel plot asymmetry was detected (**Supplemental Figure 23**).

A sensitivity analysis was conducted to include studies that measured and reported stool frequency using the same methods and units (bowel movements/week), and a MD was calculated. In 12 studies including 966 participants (21, 22, 25, 28–32, 34, 36, 37, 39, 40), fiber significantly increased stool frequency by +1.19 bowel movements/week (95% CI: 0.59, 1.78 bowel movements/week; *P* < 0.0001) compared to control; however, heterogeneity remained considerable (I^2^ = 80%; *P* < 0.00001) (Supplemental Figure 3). Psyllium significantly increased stool frequency by +3.08 bowel movements/week [95% CI: 0.61, 5.54 bowel movements/week; *P* = 0.01; I^2^ = 90% (*P* < 0.0001)] and GOS significantly increased stool frequency by +0.71 bowel movements/week [95% CI: 0.07, 1.34 bowel movements/week; *P* = 0.03; I^2^ = 68% (*P* = 0.04)]. High fiber doses (>10 g/d) increased stool frequency [MD: +1.60 bowel movements/week (95% CI: 0.71, 2.48 bowel movements/week; *P* = 0.0004); I^2^ = 85% (*P* < 0.00001)], but low fiber doses (≤10 g/d) did not, with a significant subgroup effect (*P* = 0.07). Greater treatment durations (≥4 weeks) increased stool frequency [MD: +1.33 bowel movements/week (95% CI: 0.62, 2.03 bowel movements/week; *P* = 0.0002); I^2^ = 81% (*P* < 0.00001)], but shorter durations (<4 weeks) did not; however, no significant subgroup differences were detected (*P* = 0.59). Heterogeneity remained high between studies within each subgroup (Supplemental Figure 3).

### Stool consistency

**FIGURE 4:**
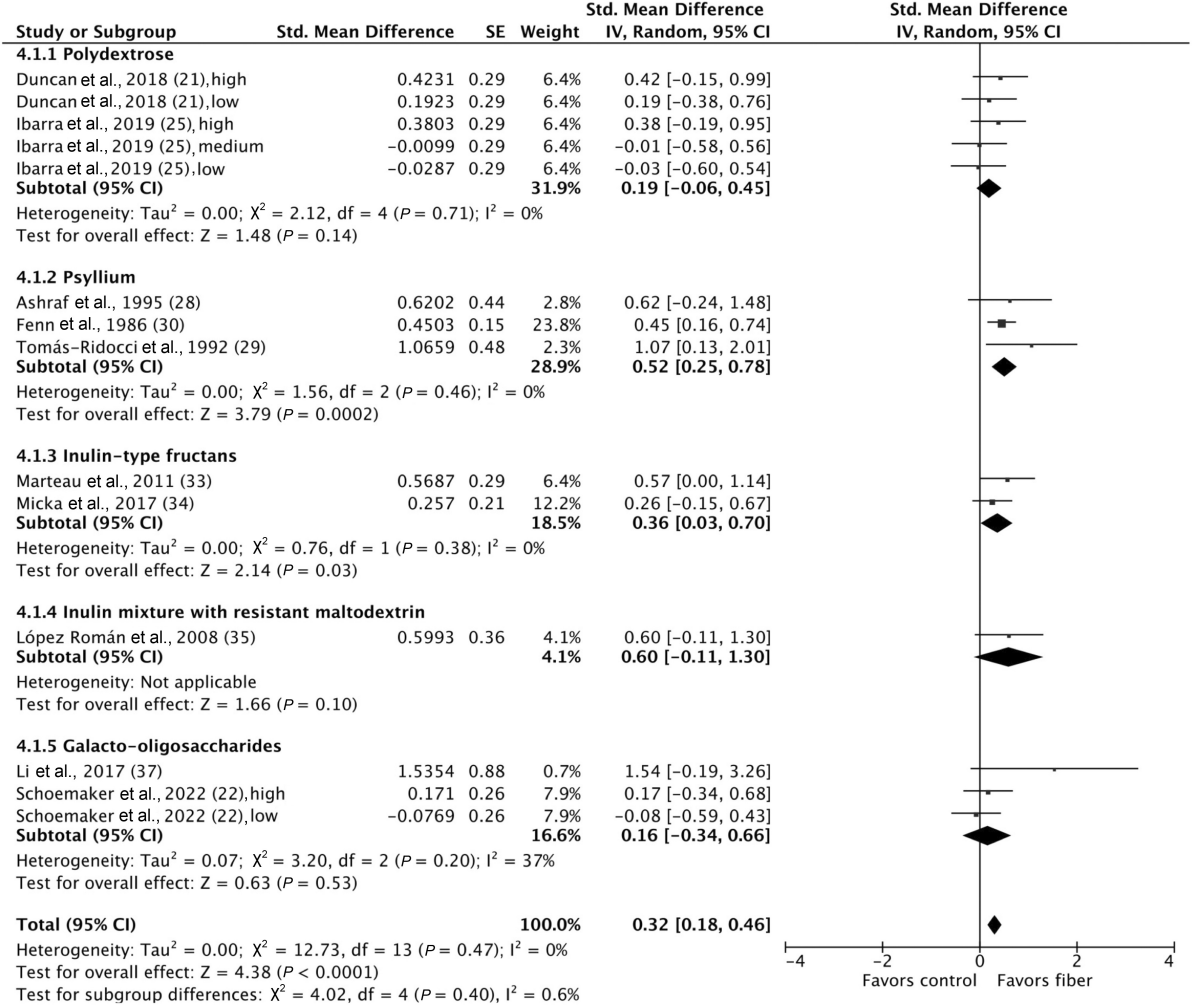
Forest plot of stool consistency in randomized controlled trials comparing fiber with control groups in adults with chronic constipation (*n* = 918). Values were calculated as standardized mean differences (95% CIs) using a random-effects model. IV, inverse variance.

Stool consistency was measured in 11 studies; however, 1 study did not report data, and these data were not obtained on request (36). Ten studies including 918 participants were included in the meta-analysis (21, 22, 25, 28–30, 33–35, 37). The methods of measuring stool consistency varied, with most studies using the Bristol Stool Form Scale or a modified version of it. Fiber significantly improved stool consistency compared to control (SMD: 0.32; 95% CI: 0.18, 0.46; *P* < 0.0001), and no significant heterogeneity was detected (I^2^ = 0%; *P* = 0.56). Subgroup analyses showed that psyllium [SMD: 0.52 (95% CI: 0.25, 0.78; *P* = 0.0002); I^2^ = 0% (*P* = 0.46)] and inulin-type fructans [SMD: 0.36 (95% CI: 0.03, 0.70; *P* = 0.03); I^2^ = 0% (*P* = 0.38)] significantly improved stool consistency, but polydextrose and GOS did not; however, no significant subgroup differences were detected (*P* = 0.40 )  (**Figure 4**). Nonprebiotic interventions significantly improved stool consistency [SMD: 0.35 (95% CI: 0.16, 0.53; *P* = 0.0002); I^2^ = 0% (*P* = 0.46)], while prebiotics or mixtures did not; however, no significant subgroup differences were detected (*P* = 0.65). High fiber doses (>10 g/d) improved stool consistency (SMD: 0.42; 95% CI: 0.26, 0.59; *P* < 0.00001), but low fiber doses (≤10 g/d) did not, with a significant subgroup effect (*P* = 0.02). All durations (less than or greater than 4 weeks) were effective at improving stool consistency. There was no significant heterogeneity detected between studies within each subgroup (Supplemental Figure 4). Funnel plot asymmetry was detected (**Supplemental Figure 24**).

### Stool weight

Stool weight was reported in 3 studies including 59 participants (28, 29, 39). Fiber did not affect stool weight compared to control (MD: 31.93 g/d; 95% CI: −3.74, 67.60 g/d; *P* = 0.08), and there was no significant heterogeneity (I^2^ = 46%; *P* = 0.15) (Supplemental Figure 5).

### Gut transit time

Whole GTT was reported in 7 studies including 485 participants (21, 25, 28, 29, 38–40). All studies measured transit time using the radio-opaque marker method. Fiber had no significant effect on whole GTT (MD: −7.5 hours; 95% CI: −18.1, 3.1 hours; *P* = 0.17), and considerable heterogeneity was detected (I^2^ = 81%; *P* < 0.00001) (Supplemental Figure 6). The effect of higher doses of fiber (>10 g/d) in decreasing whole GTT approached conventional statistical significance [MD: −12.3 hours (95% CI: −25.1, 0.5 hours; *P* = 0.06); I^2^ = 83% (*P* < 0.00001)], but low fiber doses (≤10 g/d) did not impact whole GTT, with a significant subgroup effect (*P* = 0.05). Greater treatment durations (≥4 weeks) significantly decreased whole GTT by −18.4 hours [95% CI: −31.5, –5.4 hours; *P* = 0.006; I^2^ = 77% (*P* = 0.001)], but shorter durations (<4 weeks) did not, with a significant subgroup effect (*P* = 0.003) (Supplemental Figure 6).

Regional GTT was reported in 2 studies (21, 29). Fiber had no significant impact on right, left, or rectosigmoid transit time compared to control (Supplemental Figure 7).

### Integrative symptom scores

Various questionnaires were used to measure integrative symptom scores in 5 studies including 531 participants (21, 22, 25, 38, 39). Fiber had no significant effect on integrative symptom scores [SMD: −0.15 (95% CI: −0.39, 0.08; *P* = 0.20); I^2^ = 37% (*P* = 0.12)]. Fiber doses higher or lower than 10 g/d had no effect on symptom scores. Greater treatment durations (≥4 weeks) led to significant improvements in symptom scores [SMD: −0.42 (95% CI: −0.77, −0.06; *P* = 0.02); I^2^ = 32% (*P* = 0.22)] but shorter durations (<4 weeks) did not, with a significant subgroup effect (*P* = 0.04). However, low numbers of studies contributed data in each subgroup (Supplemental Figure 8).

A sensitivity analysis was conducted for studies that measured and reported integrative symptom scores using the PAC-SYM questionnaire, and a MD was calculated. In 3 studies including 428 participants (21, 22, 25), fiber had no impact on PAC-SYM global scores [MD: −0.10 scale points (95% CI: −0.31, 0.11 scale points; *P* = 0.35); I^2^ = 48% (*P* = 0.07); a lower score denotes less severe symptoms]. In addition, fiber had no impact on the abdominal, rectal, or stool subscales of PAC-SYM (Supplemental Figures 9–12). All 3 studies measured 1 other integrative symptom score—2 measured the CCCS (21, 22) and 1 measured the Bowel Function Index (25)—and reported no difference between intervention and control groups.

### Individual gastrointestinal symptoms

The effect of fiber supplementation on the severity of individual symptoms was widely reported in various studies (Supplemental Figures 13–17).

**FIGURE 5:**
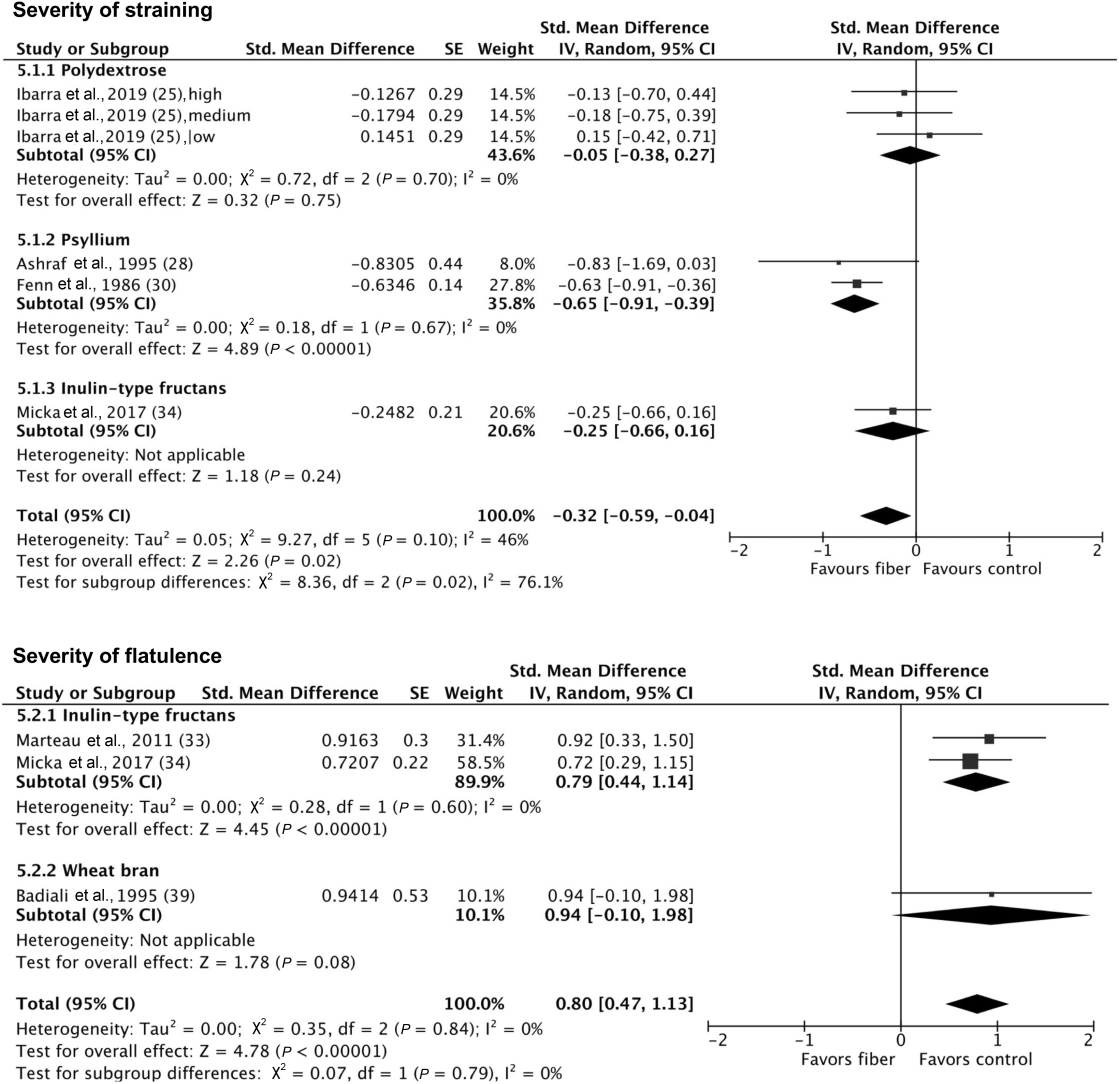
Forest plot of severity of straining (*n* = 498) and severity of flatulence (*n* = 153) in randomized controlled trials comparing fiber with control groups in adults with chronic constipation. Values were calculated as standardized mean differences (95% CIs) using a random-effects model. IV, inverse variance.

Severity of straining was reported in 4 studies including 489 participants (25, 28, 30, 34). Fiber significantly improved straining severity compared to control [SMD: *−*0.32 (95% CI: −0.59, −0.04; *P* = 0.02); I^2^ = 46% (*P* = 0.10)]. Subgroup analyses showed that only psyllium significantly improved straining severity [SMD: −0.65 (95% CI: −0.91, −0.39; *P* < 0.00001); I^2^ = 0% (*P* = 0.67)], while polydextrose and inulin-type fructans did not (**Figure 5**). High fiber doses (>10 g/d) improved straining severity [SMD: −0.45 (95% CI: −0.73, −0.16; *P* = 0.002); I^2^ = 34% (*P* = 0.002)], but low fiber doses (≤10 g/d) did not, with a significant subgroup effect (*P* = 0.09). However, a low number of studies contributed data in each subgroup (Supplemental Figure 13).

Severity of flatulence was reported in 3 studies including 153 participants (33, 34, 39). Fiber significantly worsened flatulence severity compared to control (SMD: 0.80; 95% CI: 0.47, 1.13; *P* < 0.00001), and there was no significant heterogeneity (I^2^ = 0%; *P* = 0.84). Subgroup analysis indicated that 2 studies administering inulin-type fructans significantly worsened flatulence severity [SMD: 0.79 (95% CI: 0.44, 1.14; *P* < 0.00001); I^2^ = 0% (*P* = 0.60) (Figure 5; Supplemental Figure 14)]. Fiber had no significant impact on severity of incomplete evacuation, bloating, or abdominal pain or discomfort (Supplemental Figures 15–17).

The frequency of individual symptoms was less commonly reported amongst studies, and suitable data could not be obtained for more than 1 study; therefore, a meta-analysis was not performed.

Several other individual symptoms were reported in the same way by only 1 study each and, therefore, meta-analyses were not possible. These outcomes were occurrence of hard stools (31, 35, 40), pain with defecation (31, 28, 29), sensation of anal obstruction (31, 35, 40), use of manual maneuvers (31, 35, 40), defecation difficulties (33, 38), ease of passage (37), time spent on the toilet (38), and borborygmi (33). Of note, in 1 study administering an unspecified type of fiber, the frequency of sensation of anal obstruction was significantly lower in the fiber group compared to the control group (*P* = 0.032) (40). In a study administering pectin, the time spent on the toilet was significantly lower in the fiber group compared to the control group(*P* < 0.05) (38).

### Quality of life

Two studies including 296 participants reported overall quality of life (QoL) measurements using the Patient Assessment of Constipation Quality of Life (PAC-QoL) global score (21, 25). Both studies administered polydextrose and found that it did not impact QoL scores (MD: −0.04 scale points; 95% CI: −0.19, 0.10 scale points; *P* = 0.54; a higher score indicates more severe), and heterogeneity was not significant (I^2^ = 37%; *P* = 0.17). Fiber doses higher or lower than 10 g/d had no impact on PAC-QoL. Greater treatment durations (≥4 weeks) significantly improved global scores [MD: −0.25 scale points (95% CI: −0.48, −0.03 scale points; *P* = 0.03); I^2^ = 0% (*P* = 0.66)], while shorter durations (<4 weeks) did not, with a significant subgroup effect (*P* = 0.03). However, these data derive from 2 intervention groups of the same study (Supplemental Figure 18). Fiber had no impact on QoL subscales for satisfaction, physical discomfort, worries and concerns, or psychosocial discomfort (Supplemental Figures 19–22).

### Laxative use

Two studies measured laxative use (21, 38); however, data were only reported for 1 study. In this study, pectin supplementation significantly reduced the number of days participants took laxatives compared to control [mean: 1.4 days (SD: 1.0 days) compared with 1.9 days (SD: 1.2 days), respectively; *P* < 0.01] (38).

### Adverse events

Adverse events were reported in 12 studies including 1089 participants (21, 22, 25, 28–31, 34, 37–40). In 1 study administering polydextrose, 18 events were considered to be related to the intervention and 10 events were considered to be related to the control treatment. These were all mild to moderate, except for 1 event in the polydextrose group that was severe (nausea) (25). Another study administering polydextrose reported events such as abdominal pain, but there was no link identified between treatment groups and the occurrence of adverse events (21). One study administering psyllium reported that 2 participants (18%) in the intervention group had abdominal pain, compared to none in the control group (28). Another study administering psyllium reported that 3 participants withdrew from the intervention group and 5 from the control group, for reasons related to the reported treatment side effects (30). A study administering wheat bran reported participant withdrawal due to “unbearable” abdominal pain (39). Two studies reported that while adverse events did occur, none interfered with the trial process (34, 40). Finally, 2 studies administering psyllium (29, 31), 1 administering pectin (38), and 2 administering GOS (22, 37) reported no serious adverse events associated with the fiber products.

### Compliance

Compliance was measured in 7 studies (21, 25, 30, 34, 36, 39, 40), but reported in only 3 (25, 39, 40). Of those that counted returned sachets, 1 study reported >98% compliance (25) and another reported >95% compliance in each group (40). One study reported that compliance was equally satisfactory in both groups; however, no data were provided (39). Another 2 of the 7 studies reported the numbers of participants excluded for noncompliance only: in the first RCT, 3 participants were excluded in the fiber group and none were excluded in the control group (21); and in the other, 4 participants were excluded, but the group they belonged to was not dislosed (34).

### Risk of bias at the outcome and study levels

No study was at low RoB across all domains. Bias due to the randomization process was judged to have low risk in 3 studies (25, 31, 34) and some concerns in 13 studies due to inadequate details on the methods used in the generation of the randomization sequence or for concealment of allocation (21, 22, 27–30, 32, 33, 35–40). Bias due to deviations from the intended interventions was judged to have low risk in 6 studies (21, 22, 25, 29, 37, 38), some concerns in 2 studies (28, 32), and high risk in 8 studies (30, 31, 33–36, 39, 40); as although all but 2 studies were double-blinded, many did not follow an intention-to-treat analysis. Bias across other domains varied at the outcome level (Supplemental Figures 1–22A).

## Discussion

This systematic review and meta-analysis shows that fiber supplementation is effective in the management of chronic constipation and, most importantly, identifies the optimal types of fiber, doses, and treatment durations. Psyllium supplements and fiber doses greater than 10 g/d were most effective at improving response to treatment, stool output, and straining, while treatment durations of 4 weeks or more were optimal for improving stool frequency and whole GTT.

Response to treatment was higher following fiber supplementation, with fiber increasing the likelihood of a response by 48% compared to control treatment. The type (psyllium) and doses (>10 g/d) of fiber were crucial in achieving response to treatment. Notably, just over half of the 16 included studies measured response to treatment as a binary outcome, despite recommendations by the Rome committee, and definitions of response were inconsistent across studies (41). The placebo response rate was high (41%), which is a common feature in functional bowel disorders, although the rate is higher here than in a recent meta-analysis of 73 RCTs in irritable bowel syndrome (34%) (42).

This systematic review identified fiber as having a moderate to large effect in increasing stool frequency. Psyllium led to an increase of 3 bowel movements/week, indicating that psyllium is as effective or even more effective than osmotic and stimulant laxatives, which increase stool frequencies by 2.5 bowel movements/week (43). Since normal stool frequency is 3 to 21 bowel movements/week, an increase of 3 bowel movements/week is clinically significant and could normalize stool frequency in constipation (44). Thus, psyllium may be an appropriate first-line intervention in chronic constipation prior to pharmacological therapies. Pectin also increased stool frequency; however, only 1 study contributed to this subgroup analysis.

Stool consistency was moderately improved upon fiber supplementation. Psyllium had the greatest effect on this outcome, with inulin-type fructans also modestly softening stool consistency. The effects of psyllium on stool frequency and consistency are expected, as this fiber has a high water-holding capacity that is resistant to fermentation and forms a viscoelastic substance in the gastrointestinal tract, thus softening stools (14, 45, 46). The beneficial effect of inulin-type fructans on stool consistency in chronic constipation is in line with the findings of a previous meta-analysis (47). The fermentation of inulin leads to microbiota modifications and an increase in SCFA production, resulting in an increased bacterial mass, stimulation of the enteric nervous system, and a higher water content of digesta (17). Funnel plot asymmetry was detected for stool consistency, and publication bias is likely, as 1 study did not report findings (36). Another explanation could be that the study with the largest effect size used an nonvalidated measurement tool, potentially inflating the effect size (37).

An improvement in whole GTT by 18 hours was found only when fiber was administered for a greater duration. The study that exhibited the largest effect used wheat bran (39). It is likely that the decrease in GTT with wheat bran supplementation results from modification of the gut microbiota composition and its metabolites, ultimately regulating the intestinal contractile activity (48).

Severity of straining improved with fiber supplementation, with psyllium and high fiber doses being particularly effective. However, integrative symptom scores were not affected, nor were severity of incomplete evacuation, abdominal pain or discomfort, or bloating. This suggests that fiber supplementation may be more suitable to specific cohorts of individuals who experience certain symptoms, such as infrequent bowel movements, hard stools, and straining, but not other symptoms, such as incomplete evacuation or bloating.

QoL scores were measured in studies administering polydextrose. However, QoL scores improved by only 0.25 points when polydextrose was administered for 4 weeks, which is considerably less improvement than the recommended minimal clinically meaningful difference of a 0.5- to 1-point change in PAC-QoL; therefore, it is unlikely that this will have a noticeable impact on patients’ QoL scores (49, 50). Furthermore, polydextrose was not effective at improving other patient-important outcomes, such as stool output or symptom scores.

In general, fiber was a safe intervention, although adverse events were reported. Severity of flatulence was significantly higher with fiber compared to control treatment. This is an expected side effect, as 2 of the 3 included studies, contributing to 89.9% of weight in the meta-analysis, were from inulin-type fructans, which are highly fermentable fibers that lead to microbiota-mediated gas production (45). This finding agrees with a previous meta-analysis showing that fiber increases flatulence (18). One study reported abdominal pain leading to withdrawal from a participant receiving wheat bran. These adverse effects highlight the importance of selecting the appropriate type of fiber and dose regime, both for optimum effectiveness and for the prevention of adverse effects. Psyllium supplements should be used in favor of wheat bran, and individuals should gradually increase their fiber intakes to prevent sudden symptom exacerbation (9).

The findings of this review have important implications. National guidelines for chronic constipation suggest increasing fiber intake, but there is a lack of detail on the types, doses, and durations of fiber supplementation (7, 9–11). This may result in nonstandardized recommendations in clinical care, with patients left to choose the type and regime of fiber supplements, likely leading to dissatisfaction (8, 51). This review provides important evidence on the optimal fiber supplementation, which can support clinicians in providing standardized and effective recommendations to patients with chronic constipation. A previous systematic review showed a dose-dependent response for fiber doses >15 g/d (18). The current review shows that a smaller cutoff of >10 g/d is still effective; this dose may be more realistic to ingest and more tolerable than doses >15 g/d, potentially increasing compliance and satisfaction with treatment.

This is the largest systematic review and meta-analysis to investigate the effect of fiber supplementation in chronic constipation. It identified more than double the number of trials than the most recent systematic review (18), thus allowing for more robust subgroup analyses of specific fibers, doses, and treatment durations. This review strictly adhered to recommendations from the Cochrane Handbook and PRISMA guidelines. Efforts were made to search gray literature with no language restrictions, reducing publication bias. Limitations of this review include significant heterogeneity amongst outcomes, explained by the types of fiber and differences in the methods used to measure outcomes. While included studies provided further insight into the effects of certain fiber types in chronic constipation (e.g., psyllium), for other types (e.g., pectin), only single studies contributed to the subanalyses; therefore, how effective these fiber types might be remains unclear. No studies in this review had a low risk of bias.

In conclusion, this meta-analysis provides evidence that psyllium is the most efficacious investigated fiber at providing constipation relief, with improvements in stool frequency and severity of straining, which highlights psyllium's potential to be used as a first-line strategy for the management of constipation. Doses >10 g/d and durations of at least 4 weeks appear to be optimal for several constipation symptoms. However, there was a low number of studies and substantial heterogeneity between studies in the subgroup analyses. This limits confidence in the interpretations of the findings of the subgroup analyses regarding the types and regimes of fiber supplementation (26). It was also found that fiber may lead to increased flatulence, highlighting the need to address this with patients and apply strategies to mitigate this. These findings provide evidence for the optimal types and regimes of fiber supplementation that could impact clinical care and recommendations to patients, ultimately improving their care and treatment response.

## Supplementary Material

nqac184_Supplemental_File

## Data Availability

Data described in the manuscript, code book, and analytic code will be made available upon request pending application and approval by the corresponding author.

## Notes

This systematic review was part of a project funded by the General and Education Trust Fund, British Dietetic Association (grant ID: 19/14).

The funders had no role in the design, conduct, or publishment of this review.

Supplemental Methods 1, Supplemental Table 1, and Supplemental Figures 1–24 are available from the “Supplementary data” link in the online posting of the article and from the same link in the online table of contents at https://academic.oup.com/ajcn/.

Abbreviations used: CCCS, Cleveland Clinic Constipation Score; GOS, galacto-oligosaccharides; GTT, gut transit time; MD, mean difference; IBS-C, irritable bowel syndrome with constipation; PAC-QoL, Patient Assessment of Constipation Quality of Life; PAC-SYM, Patient Assessment of Constipation Symptoms; PRISMA, Preferred Reporting Items for Systematic Reviews and Meta-Analyses; QoL, quality of life; RCT, randomized controlled trial; RoB, risk of bias; RR, risk ratio; SMD, standardized mean difference.
