# Supplementary material for: The Effect of Fiber Supplementation on Chronic Constipation in Adults: An Updated Systematic Review and Meta-Analysis of Randomized Controlled Trials
Source: Am J Clin Nutr. 2022 Jul 11;116(4):953–69. doi: 10.1093/ajcn/nqac184 (PMC9535527; doi:10.1093/ajcn/nqac184)
Supplement: nqac184_Supplemental_File [file nqac184_supplemental_file.pdf]

On-line Supplementary Material

The effect of fiber supplementation on chronic constipation in adults: an updated systematic review and meta-analysis of randomized controlled trials

Authors: Alice van der Schoot, Candice Drysdale, Kevin Whelan, Eirini Dimidi

Department of Nutritional Sciences, King's College London, London, UK

Table of Contents

SECTION 1. SEARCH STRATEGY

Supplementary Methods 1: A detailed search strategy for RCTs investigating the effect of fiber supplementation on chronic constipation in adults. 4

SECTION 2. CHARACTERISTICS OF EXCLUDED STUDIES

Supplementary Table 1: Characteristics of excluded studies 9

SECTION 3. FOREST PLOTS WITH RISK OF BIAS AND SUBGROUP ANALYSES (TYPE OF FIBER, PREBIOTIC STATUS, DOSE, DURATION)

Supplementary Figure 1: Response to treatment 14

Supplementary Figure 2: Stool frequency (standardized mean difference) 18
Supplementary Figure 3: Stool frequency (mean difference) 22

Supplementary Figure 4: Stool consistency 26
_Toc109910616
Supplementary Figure 5: Stool weight 30

Supplementary Figure 6: Whole gut transit time 32

Supplementary Figure 7: Regional gut transit time 36

Supplementary Figure 8: Integrative symptom score 37

Patient Assessment of Constipation Symptoms (PAC-SYM)
Supplementary Figure 9: Global 42
Supplementary Figure 10: Abdominal 47
Supplementary Figure 11: Rectal 50
Supplementary Figure 12: Stool 53

Supplementary Figure 13: Severity of straining 56

Supplementary Figure 14: Severity of flatulence 60
_Toc109910633
Supplementary Figure 15: Severity of incomplete evacuation 63

Supplementary Figure 16: Severity of bloating 65

Supplementary Figure 17: Severity of abdominal pain/discomfort 68

Patient Assessment of Constipation Quality of Life (PAC-QoL)
Supplementary Figure 18: Global 72
Supplementary Figure 19: Satisfaction 75

Supplementary Figure 20: Physical discomfort 79
Supplementary Figure 21: Worries and concerns 83
Supplementary Figure 22: Psychosocial discomfort 86

SECTION 4. FUNNEL PLOTS TO ASSESS PUBLICATION BIAS

Supplementary Figure 23: Funnel plot of stool frequency 89
Supplementary Figure 24: Funnel plot of stool consistency 89

REFERENCES 90

Section 1. Search Strategy

Supplementary Methods 1 A detailed search strategy for RCTs investigating the effect of fiber supplementation on chronic constipation in adults.

MEDLINE

MEDLINE was searched via OvidSP (from 1946). The search was performed on 18th March 2022 and yielded 1155 records.

constipation.mp. OR exp Constipation/ OR “functional constipation”.mp. OR “primary constipation”.mp. OR “chronic constipation”.mp. OR “idiopathic constipation”.mp. OR “slow transit constipation”.mp. OR constipated.mp. OR “def?ecation disorder*”.mp. OR “Evacuation disorder*”.mp. OR “gastrointestinal transit”.mp. OR exp Gastrointestinal Transit/ OR “gut transit”.mp. OR “slow transit”.mp. OR “hard stool*”.mp. OR “lumpy stool*”.mp. OR “hard f?eces”.mp. OR straining.mp. OR “incomplete evacuation”.mp. OR infrequent bowel movement*.mp.
AND
exp Adult/ or exp Young Adult/ OR adult*.mp. OR elderly.mp. OR exp Aged/ OR individual*.ab. OR volunteer*.ab. OR participant*.ab. OR subject*.ab. OR patient*.ab. OR human*.ab.
AND
“Dietary fiber”.mp. OR exp Dietary Fiber/ OR “Dietary fibre”.mp. OR Fiber.mp. OR Fibre.mp. OR exp Plant Extracts/ OR plant extract*”.mp. OR “resistant starch”.mp. OR “resistant maltodextrin”.mp. OR “digestion-resistant maltodextrin”.mp. OR isomaltodextrin.mp. OR cellulose.mp. OR exp Cellulose/ OR pectin.mp. or exp Pectins/ OR lignin.mp. OR exp Lignin/ OR exp beta-Glucans/ OR beta-glucan*.mp. OR “soluble maize fib*”.mp. OR “Soluble corn fib*”.mp. OR pullulan.mp. OR glucomannan.mp. OR konjac.mp. OR exp Amorphophallus/ OR Galactomannan.mp. OR Arabinan*.mp. OR Arabinogalactan*.mp. OR Arabinoxylan*.mp. OR Polydextrose.mp. OR bran.mp. cereal.mp. OR “acacia gum”.mp. or exp Gum Arabic/ OR “Partially hydroly?ed guar gum”.mp. OR “guar gum”.mp. OR psyllium.mp. OR exp Psyllium/ OR “Ispaghula husk”.mp. OR “Plantago ovata”.mp. OR exp Plantago/ OR metamucil.mp. OR Fybogel.mp. OR exp Fructans/ or fructan*.mp. OR inulin.mp. OR exp Inulin/ OR “inulin type fruct*”.mp. OR chicory.mp. OR exp Chicory/ OR exp Oligosaccharides/ OR oligosaccharide*.mp. OR fructooligosaccharide*.mp. OR oligofructose.mp. OR galactooligosaccharide*.mp. OR transgalactooligosaccharide*.mp. OR isomaltooligosaccharide*.mp OR exp Galactans/ or galactan*. mp.OR mannanoligosaccharide*.mp. OR xylooligosaccharide*.mp. OR oligomer*.mp. OR sc-FOS.mp. OR methylcellulose.mp. OR exp Methylcellulose/
AND

Randomi?ed controlled trial.pt. OR controlled clinical trial.pt. OR exp Controlled Clinical Trial/ OR exp Clinical Trial/ OR randomi?ed.ti,ab. OR randomly.ti,ab. OR random*.ti,ab OR placebo.ti,ab. OR trial.ti,ab. OR control*.ti,ab OR "clinical trial".ti,ab OR clinical trials as topic.sh.<br><br>NOT<br><br>exp animals/ not humans.sh. OR (rat or rats or mouse or mice or swine or porcine or murine or sheep or lambs or pigs or piglets or rabbit or rabbits or cat or cats or dog or dogs or cattle or bovine or monkey or monkeys or trout or marmoset*).ti.

EMBASE

EMBASE was searched via OvidSP (from 1946). The search was performed on 18th March 2022 and yielded 3106 records.

exp constipation/ OR exp chronic constipation/ OR constipation.mp. OR 'functional constipation'.mp. OR 'primary constipation'.mp. OR 'chronic constipation'.mp. OR 'idiopathic constipation'.mp. OR 'slow transit constipation'.mp. OR constipated.mp. OR exp defecation disorder/ OR 'Def?ecation disorder*'.mp. OR 'evacuation disorder*'.mp. OR 'Gastrointestinal transit'.mp. OR exp gastrointestinal transit/ OR 'gut transit'.mp. OR 'slow transit'.mp. OR 'hard stool*'.mp. OR 'lumpy stool*'.mp. OR 'hard f?eces'.mp. OR exp hard feces/ OR straining.mp. OR 'incomplete evacuation'.mp. OR 'infrequent bowel movement*'.mp.
AND
exp young adult/ OR exp adult/ OR adult*.mp. OR elderly.mp. OR exp aged/ OR individual*.ab. OR volunteer*.ab. OR participant*.ab. OR subject*.ab. OR patient*.ab. OR human*.ab.
AND
'dietary fiber'.mp. OR exp dietary fiber/ OR 'dietary fibre'.mp. OR fiber.mp. OR exp fiber/ OR fibre.mp. OR prebiotic*.mp. OR 'non starch polysaccharide*'.mp. OR exp plant extract/ OR 'plant extract*'.mp. OR 'resistant starch'.mp. OR 'resistant maltodextrin'.mp. OR 'digestion-resistant maltodextrin'.mp. OR isomaltodextrin.mp. OR exp cellulose/ OR cellulose.mp. OR exp pectin/ OR pectin.mp. OR exp lignin/ OR lignin.mp. OR exp beta glucan/ OR beta-glucan*.mp. OR 'soluble maize fib*'.mp. OR 'soluble corn fib*'.mp. OR pullulan.mp. OR exp pullulan/ OR glucomannan.mp. OR konjac.mp. OR exp Amorphophallus konjac/ OR Galactomannan.mp. OR exp galactomannan/ OR arabinan*.mp. OR exp arabinogalactan/ OR arabinogalactan*.mp . OR exp arabinoxylan/ or arabinoxylan*.mp. OR polydextrose.mp. OR exp bran/ OR bran.mp. OR cereal.mp. OR exp cereal/ OR 'acacia gum'.mp. OR exp gum arabic / OR 'partially hydroly?ed guar gum'.mp. OR 'guar gum'.mp. OR exp guar gum/ OR psyllium.mp.

OR exp ispagula/ OR ‘ispaghula husk’.mp. OR ‘plantago ovata’.mp.<br>OR exp Plantago ovata/ OR metamucil.mp. OR fybogel.mp. OR exp fructan/<br>or fructan*.mp. OR exp inulin/ or inulin.mp. OR ‘inulin type fruct*’.mp. OR<br>chicory.mp. OR exp chicory/ OR exp oligosaccharide/ or oligosaccharide*.mp<br>.OR exp fructose oligosaccharide/ or fructooligosaccharide*.mp. OR<br>oligofructose.mp. OR exp galactose oligosaccharide/ or galactooligosaccharide*.mp.<br>OR transgalactooligosaccharide*.mp. OR isomaltooligosaccharide*.mp.<br>OR exp galactan/ or galactan*.mp. OR mannanoligosaccharide*.mp.<br>OR xylooligosaccharide*.mp. OR exp oligomer/ or oligomer*.mp. OR sc-FOS.mp.<br>OR methylcellulose.mp. OR exp methylcellulose/
AND
exp randomized controlled trial/ OR exp controlled clinical trial/ OR random$.ti,ab.<br>OR exp randomization/ OR exp intermethod comparison/ OR placebo.ti,ab. OR<br>(compare or compared or comparison).ti. OR ((evaluated or evaluate or evaluating<br>or assessed or assess) and (compare or compared or comparing or<br>comparison)).ab. OR (open adj label).ti,ab. OR ((double or single or doubly or<br>singly) adj (blind or blinded or blindly)).ti,ab. OR exp double blind procedure/ OR<br>parallel group$1.ti,ab. OR (crossover or cross over).ti,ab. OR ((assign$ or match or<br>matched or allocation) adj5 (alternate or group$1 or intervention$1 or patient$1 or<br>subject$1 or participant$1)).ti,ab. OR (assigned or allocated).ti,ab. OR (controlled<br>adj7 (study or design or trial)).ti,ab. OR (volunteer or<br>volunteers).ti,ab. OR exp human experiment/ OR trial.ti.<br><br>NOT<br><br>(random$ adj sampl$ adj7 ('cross section$' or questionnaire$1 or survey$ or<br>database$1)).ti,ab. not (comparative study/ or controlled study/<br>or randomi?ed controlled.ti,ab. or randomly assigned.ti,ab.) OR Cross-sectional<br>study/ not (randomized controlled trial/ or controlled clinical study/ or controlled<br>study/ or randomi?ed controlled.ti,ab. or control group$1.ti,ab.) OR<br>(((case adj control$) and random$) not randomi?ed controlled).ti,ab. OR<br>(Systematic review not (trial or study)).ti. OR (nonrandom$ not random$).ti,ab. OR<br>'Random field$'.ti,ab. OR (random cluster adj3 sampl$).ti,ab. OR (review.ab. and<br>review.pt.) not trial.ti. OR 'we searched'.ab. and (review.ti. or review.pt.) OR<br>'update review'.ab. OR (databases adj4 searched).ab. OR (rat or rats or mouse or<br>mice or swine or porcine or murine or sheep or lambs or pigs or piglets or rabbit or<br>rabbits or cat or cats or dog or dogs or cattle or bovine or monkey or monkeys or<br>trout or marmoset$1).ti. and animal experiment/ OR Animal experiment/ not (human<br>experiment/ or human/)

Web of Science

Web of Science was searched via the Web of Knowledge portal (from 1900). The search was performed on 18th March 2022 and yielded 3906 records.

(constipation OR “functional constipation” OR “primary constipation” OR “chronic constipation” OR “idiopathic constipation” OR “slow transit constipation” OR constipated OR “def?ecation disorder*” OR “Evacuation disorder*” OR “gastrointestinal transit” OR “gut transit” OR “slow transit” OR “hard stool*” OR “lumpy stool*” OR “hard f?eces” OR straining OR “incomplete evacuation” OR “infrequent bowel movement*”) as TOPIC
AND
(Adult* OR elderly OR individual* OR volunteer* OR participant* OR subject* OR patient* OR human*) as TOPIC
AND
(Fiber OR Fibre OR “Dietary fiber” OR “Dietary fibre” OR prebiotic* OR “non starch polysaccharide*” OR “plant extract*” OR “resistant starch” OR “resistant maltodextrin” OR “digestion-resistant maltodextrin” OR isomaltodextrin OR cellulose OR pectin OR lignin OR “beta-glucan*” OR “soluble maize fib*” OR “Soluble corn fib*” OR pullulan OR glucomannan OR konjac OR Galactomannan OR Arabinan* OR Arabinogalactan* OR Arabinoxylan* OR Polydextrose OR bran OR cereal OR “acacia gum” OR “Partially hydroly?ed guar gum” OR “guar gum” OR psyllium OR “Ispaghula husk” OR “Plantago ovata” OR metamucil OR Fybogel OR fructan* OR inulin OR “inulin type fruct*” OR chicory OR oligosaccharide* OR fructooligosaccharide* OR oligofructose OR galactooligosaccharide* OR transgalactooligosaccharide* OR isomaltooligosaccharide* OR galactan* OR mannanoligosaccharide* OR xylooligosaccharide* OR oligomer* OR "sc-FOS" OR methylcellulose) as TOPIC
AND
(“Randomi?ed controlled trial” OR “controlled clinical trial” OR “Clinical Trial” OR randomi?ed OR randomly OR random* OR placebo OR trial OR control*) as TOPIC

CENTRAL

The Cochrane Central Register of Controlled Trials (CENTRAL) was searched via the Cochrane Library (all years). The search was performed on 18th March 2022 and yielded 667 records.

Title, abstract, keywords	Dietary Fiber OR Fibre OR Prebiotic OR Psyllium
	AND
Search all text	Constipation

Clinical Trials Register

The following combinations of terms were entered into CinicalTrials.gov. The search was performed on 18th March 2022 and yielded 30 records.

Interventional Studies | constipation | fiber OR fibre OR pysllium OR ispaghula OR plantago ovata OR metamucil OR prebiotic OR resistant starch OR resistant maltodextrin OR cellulose OR pectin OR lignin OR glucan OR glucomannan OR konjac OR galactomannan OR arabinoxylan OR polydextrose | Adult, Older Adult | Studies that accept healthy volunteers
Interventional Studies | constipation | guar gum OR fybogel OR fructan OR inulin OR chicory OR prebiotic OR oligosaccharide OR fructooligosaccharide OR oligofructose OR 43. galactooligosaccharide OR transgalactooligosaccharide OR isomaltooligosaccharide OR xylooligosaccharide OR sc-FOS | Adult, Older Adult | Studies that accept healthy volunteers |
Interventional Studies | constipation | isomaltodextrin OR beta-glucan OR digestion-resistant maltodextrin OR pullulan OR arabinan OR arabinogalactan OR bran OR acacia gum OR galactan OR methylcellulose OR mannanoligosaccharide | Adult, Older Adult | Studies that accept healthy volunteers

Section 2. Characteristics of excluded studies

Supplementary Table 1 Characteristics of excluded studies

Study	Reason for exclusion
1. Ito et al, 2022	Participants did not have chronic constipation.
2. Müller et al, 2020	Symptom of gut transit > 35 hr is not indicative of constipation.
3. Alyousif et al, 2020	Not in constipation as participants were described as having normal bowel habits. Control food contained too much fiber (0.8-1.1 g per cookie).
4. Zhang et al, 2019	The different intervention arms do not allow the effect of the fiber be isolated.
5. Watson et al, 2019	Symptom of low stool frequency, specified as 2 - 4 days without a bowel movement/week is not indicative of constipation.
6. Machado et al, 2019	Participants not detailed to be constipated in inclusion criteria. Control breakfast contains too much fiber (2.63 g).
7. Huaman et al, 2019	Not an appropriate comparator (learning to correct dyssynergic defecation through 2-3 sessions of biofeedback per week). 3.5g psyllium husk (undefined measurement) is not a sufficient dose of fiber.
8. Hara et al, 2019	Participants with < 5 bowel movements/week were considered constipated, which is not in line with this review's constipation criteria.
9. Gokan et al, 2019	Participants had 3-5 bowel movement/week, which is not indicative of constipation.
10. Chu et al, 2019	Effect of the fiber cannot be isolated as the intervention contains other active ingredients e.g. aloe vera gel.
11. Cheng et al, 2019	Dose of fiber is not high enough: 3.5g psyllium (undefined measurement).
12. Anzawa et al, 2019	A tendency to constipation with 3–5 bowel movements/week is not indicative of constipation. Daily fiber dose of intervention is too low (inulin content 2 g out of 100 g drink).
13. Hoshino et al, 2019	Participants had 2-5 bowel movements/week, which is not indicative of constipation. Control food contained too much fiber (6.8 g fiber per jelly serving).
14. Vandeputte et al, 2017	Duplicate of included study (Micka et al, 2017) and outcomes reported in this article are not of interest in this review.
15. Nishimura et al, 2017	Participants had 2-4 bowel movements/week, which is not indicative of constipation. Control food contained too much fiber (1.7 g fiber per bar).
16. Kinoshita et al, 2017	Participants had 3-5 bowel movements/week, which is not indicative of constipation.

17. Aoe et al, 2017	Participants defecate more than 5 days weekly, which is not indicative of constipation.
18. Ishida et al, 2017	A tendency to constipation with 4 or fewer bowel movements/week is not indicative of constipation.
19. Hamaguchi et al, 2017	A tendency to constipation with 2-5 bowel movements/week is not indicative of constipation.
20. Gomez Castrejon & Campos Vega 2017	Conference abstract. Contact with author confirmed participants were not constipated.
21. Buddington et al, 2017	Participants had 1-3 bowel movements per week which is not indicative of constipation.
22. Nakazeko et al, 2016	A tendency to constipation with 3-5 bowel movements/week is not indicative of constipation. Fiber content of control food is too high (2.9 g). Outcomes are not of interest to this review.
23. Major et al, 2016	Duration of treatment too short (7 days).
24. Hamaguchi et al, 2016	A tendency to constipation with 2-4 bowel movements/week is not indicative of constipation.
25. Hoffmann Sardá et al, 2016	Not in constipation.
26. Gabrielli et al, 2016	Dose of fiber is not high enough: 3.5g psyllium (undefined measurement).
27. Abellan Ruiz et al, 2016	Participants were recruited who did not have a daily defecation habit, which is not indicative of constipation.
28. De Souza et al, 2015	Effect of fiber cannot be isolated, as the intervention contains other ingredients. Only 10/52 g is FOS or inulin.
29. Koeda et al, 2015	Cross-over study with an inadequate washout period (1 week) and data from the first period only cannot be extracted. Control bread contains too much fiber (1.8 g fiber per slice, 2 slices per day). Criteria for “tendency to constipation” not stated.
30. Kishimoto et al, 2014	A tendency to constipation with 2-4 bowel movements/week is not indicative of constipation. Control food contained too much fiber per meal (0.6 g).
31. Scheid et al, 2014	Effect of the fiber cannot be isolated as the intervention contains other ingredients. The supplement group received a dose of 18 g of freeze dried yacon powder containing 7.4 g FOS.
32. Polymeros et al, 2014	Not a randomized controlled trial (single treatment design with no control). Dose of fiber is too low (5 mg).
33. Lawton et al, 2013	Not in constipation, as study recruited healthy participants. Not an appropriate comparator (non-intervention).

34. Granata et al 2013	Not a randomized controlled trial as the elderly constipated group did not have a control. Fiber cannot be isolated from the synbiotic intervention. Participants were living in a rest home and therefore not in an outpatient or community setting.
35. Asano et al, 2013	Dose of fiber too low (5.0 mg/100 ml drink).
36. Francois et al, 2012	Not in constipation, as study recruited healthy participants.
37. Yen et al, 2011	All participants were in a long-term nursing home and therefore not in an outpatient or community setting. Study design is not randomized.
38. Tomono et al, 2010	Participants had 2-10 bowel movements per 2 weeks, and this range allowance is not indicative of constipation.
39. Surakka et al, 2009	Symptoms of “either difficulties in intestinal function (< 5 bowel movements/week) or continuous difficulties in defecation or both” is not considered indicative of constipation for this review.
40. Pilipenko et al, 2009	All participants had IBS-C, not chronic constipation.
41. Park & Jhon 2009	Duration of treatment too short (6 days). Control diet was not an appropriate comparator.
42. Liu et al, 2009	Not an appropriate comparator (self-controlled).
43. Hengst et al, 2009	Contact with author confirmed that the study was not in constipation, as healthy volunteers were recruited.
44. Paineau et al, 2008	Not in constipation.
45. Chen et al, 2008	Not a randomized controlled trial.
46. Geyer et al, 2008	Study was in healthy volunteers and constipation listed in exclusion criteria. Yacon syrup intervention contained other ingredients in addition to fiber (only 32% FOS).
47. Kim et al, 2006	Effect of fiber cannot be isolated as the intervention contains fiber supplements and whole foods (e.g. prunes, walnuts).
48. Hongisto et al, 2006	Effect of fiber cannot be isolated from rye bread intervention.
49. Lin et al, 2005	Not in adults (participants 15-19 years old). Constipation not mentioned in inclusion criteria.
50. Kim et al, 2004	Effect of fiber cannot be isolated as ear mushroom powder as it contains additional ingredients to fiber (61.9 % fiber).
51. Den Hond et al, 2000	Duration of treatment periods too short (1 week).
52. Teuri & Korpela 1998	Not in an outpatient or community setting as all participants lived in homes maintained for the elderly.

53. Sasaki, 1998	Effect of fiber cannot be isolated from the agar jelly.
54. Patrick et al, 1998	Not in an outpatient or community setting as study was in nursing home residents.
55. Neal, 1995	Effect of fiber cannot be isolated from the power pudding as it contains other ingredients. No mention of study duration but aim was to determine whether pudding induced softer stools in 3 days. Elderly were homebound and had other illnesses e.g. Parkinson's.
56. Marsicano et al, 1995	Not in constipation.
57. Gibson et al, 1995	Not in constipation. Not a randomized controlled trial.
58. Cheskin et al, 1995	Cross-over study with no washout period. Data from the first period only cannot be extracted and the author could not provide this data.
59. Bobbio et al, 1995	Dose of glucomannan fiber intervention too low (3 g daily).
60. Takahashi et al, 1994	Not an appropriate comparator as no control intervention stated.
61. Odes et al, 1993	Effect of fiber cannot be isolated as cereal contained other ingredients in addition to fiber (12.5 g fiber per 100 g). No washout period.
62. Quaade et al, 1990	No mention of constipation in inclusion criteria. No washout period.
63. Astrup et al, 1990	Duplicated results of Quaade et al, 1990 (excluded).
64. Prior & Whorwell 1987	All participants had IBS, not chronic constipation.
65. Pergola, 1987	Not an appropriate comparator, as fiber intervention was compared with a therapeutic product.
66. Borgia et al, 1986	Not an appropriate comparator, as fiber intervention was compared with a medicinal herb.
67. Pulpeiro et al, 1985	Not exclusively in adults as age range was 15-78 years. Not an adequate washout period.
68. Sculati & Giampiccoli, 1984	Not an appropriate comparator.
69. Pulpeiro et al, 1984	Duplicated results of Pulpeiro 1985 (excluded).
70. Corinaldesi et al, 1982	Cross-over study with no washout period between interventions. Data from the first period only cannot be extracted.
71. Bjorneklett et al, 1978	Not an appropriate comparator as the study compares two fiber interventions.

72. Schlagheck et al, 1996	Only abstract located. No response from author to assess eligibility.
73. Danese S, 2016; NCT01540669	Clinical trial terminated due to slow recruitment, and no response from authors to assess eligibility or obtain any available data.
74. Benamouzig R, 2018; NCT01847950	Clinical trial was completed and confirmed to be eligible after contact with principal investigator. Study was not published according to sponsor policy, and data could not be shared due to a confidentiality agreement with the partner.
75. Gendre D, 2018; NCT03707002	Clinical trial was completed but is unpublished. No response from trial contacts when details were requested for eligibility assessment
76. Danone, 2017; NCT02461485	Clinical trial completed but is unpublished. Trial contact could not provide intervention details for eligibility assessment because the study is still unpublished.
77. Ning L, 2016; NCT02291354	Ongoing clinical trial and no response from authors to assess eligibility or obtain available data.

Section 3. Forest plots with risk of bias and subgroup analyses (type of fiber, prebiotic status, dose, duration)

Response to treatment

Study or Subgroup	Fiber Events	Fiber Total	Control Events	Control Total	Weight	Risk Ratio M-H, Random, 95% CI	A	B	C	D	E	F
Badiali 1995	3	13	5	12	3.2%	0.55 [0.17, 1.83]	?	−	−	?	?	−
Fenn 1986	90	104	46	97	14.5%	1.82 [1.46, 2.28]	?	−	−	−	?	−
Ibarra (high) 2019	38	48	10	16	11.2%	1.27 [0.84, 1.90]	+	+	+	+	?	?
Ibarra (medium) 2019	34	48	10	16	10.9%	1.13 [0.74, 1.73]	+	+	+	+	?	?
Ibarra (low) 2019	24	48	10	16	10.0%	0.80 [0.50, 1.28]	+	+	+	+	?	?
López Román 2008	10	15	4	17	4.7%	2.83 [1.12, 7.17]	?	−	−	+	−	−
Schoemaker (high) 2022	26	44	7	22	7.3%	1.86 [0.96, 3.59]	?	+	+	+	?	?
Schoemaker (low) 2022	18	45	7	21	6.8%	1.20 [0.59, 2.42]	?	+	+	+	?	?
Tomás-Ridocci 1992	10	10	5	10	8.0%	1.91 [1.04, 3.50]	?	+	−	−	−	−
Waitzberg 2012	9	28	10	32	6.3%	1.03 [0.49, 2.16]	?	−	−	−	?	−
Xu 2014	26	40	7	40	6.7%	3.71 [1.83, 7.56]	?	+	+	+	?	?
Yang 2021	23	30	13	30	10.3%	1.77 [1.12, 2.79]	+	−	−	−	?	−
**Total (95% CI)**		**473**		**329**	**100.0%**	**1.48 [1.17, 1.88]**						
Total events	311		134									

Heterogeneity: $Tau^2 = 0.09$; $Chi^2 = 25.87$, df = 11 (P = 0.007); $I^2 = 57\%$

Test for overall effect: Z = 3.24 (P = 0.001)

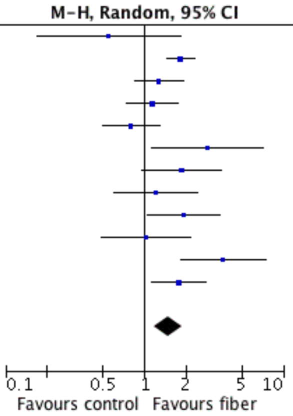

Supplementary Figure 1A Forest plot of response to treatment in randomized controlled trials comparing fiber with control in adults with chronic constipation (n=802). Values were calculated as risk ratio (95% CIs) using a random-effects model. Risk of bias key: A=bias arising from the randomization process; B=bias arising from deviations from intended interventions; C=bias due to missing outcome data; D=bias in measurement of the outcome; E=bias in selection of the reported result F=overall risk of bias. Abbreviations: M-H, Mantel-Haenszel; RR, risk ratio.

Study or Subgroup	Fiber Events	Fiber Total	Control Events	Control Total	Weight	Risk Ratio M-H, Random, 95% CI
**3.10.1 Prebiotics**						
Schoemaker (high) 2022	26	44	7	22	7.3%	1.86 [0.96, 3.59]
Schoemaker (low) 2022	18	45	7	21	6.8%	1.20 [0.59, 2.42]
**Subtotal (95% CI)**		**89**		**43**	**14.1%**	**1.51 [0.94, 2.45]**
Total events	44		14			
Heterogeneity: Tau^2 = 0.00; Chi^2 = 0.79, df = 1 (P = 0.37); I^2 = 0%						
Test for overall effect: Z = 1.69 (P = 0.09)						
**3.10.2 Mixture of prebiotics with non-prebiotics**						
López Román 2008	10	15	4	17	4.7%	2.83 [1.12, 7.17]
Waitzberg 2012	9	28	10	32	6.3%	1.03 [0.49, 2.16]
**Subtotal (95% CI)**		**43**		**49**	**11.0%**	**1.64 [0.61, 4.42]**
Total events	19		14			
Heterogeneity: Tau^2 = 0.33; Chi^2 = 2.79, df = 1 (P = 0.10); I^2 = 64%						
Test for overall effect: Z = 0.98 (P = 0.33)						
**3.10.3 Non-prebiotics**						
Badiali 1995	3	13	5	12	3.2%	0.55 [0.17, 1.83]
Fenn 1986	90	104	46	97	14.5%	1.82 [1.46, 2.28]
Ibarra (high) 2019	38	48	10	16	11.2%	1.27 [0.84, 1.90]
Ibarra (medium) 2019	34	48	10	16	10.9%	1.13 [0.74, 1.73]
Ibarra (low) 2019	24	48	10	16	10.0%	0.80 [0.50, 1.28]
Tomás-Ridocci 1992	10	10	5	10	8.0%	1.91 [1.04, 3.50]
Xu 2014	26	40	7	40	6.7%	3.71 [1.83, 7.56]
Yang 2021	23	30	13	30	10.3%	1.77 [1.12, 2.79]
**Subtotal (95% CI)**		**341**		**237**	**74.9%**	**1.46 [1.08, 1.97]**
Total events	248		106			
Heterogeneity: Tau^2 = 0.11; Chi^2 = 22.36, df = 7 (P = 0.002); I^2 = 69%						
Test for overall effect: Z = 2.49 (P = 0.01)						
**Total (95% CI)**		**473**		**329**	**100.0%**	**1.48 [1.17, 1.88]**
Total events	311		134			
Heterogeneity: Tau^2 = 0.09; Chi^2 = 25.87, df = 11 (P = 0.007); I^2 = 57%						
Test for overall effect: Z = 3.24 (P = 0.001)						
Test for subgroup differences: Chi^2 = 0.06, df = 2 (P = 0.97), I^2 = 0%						

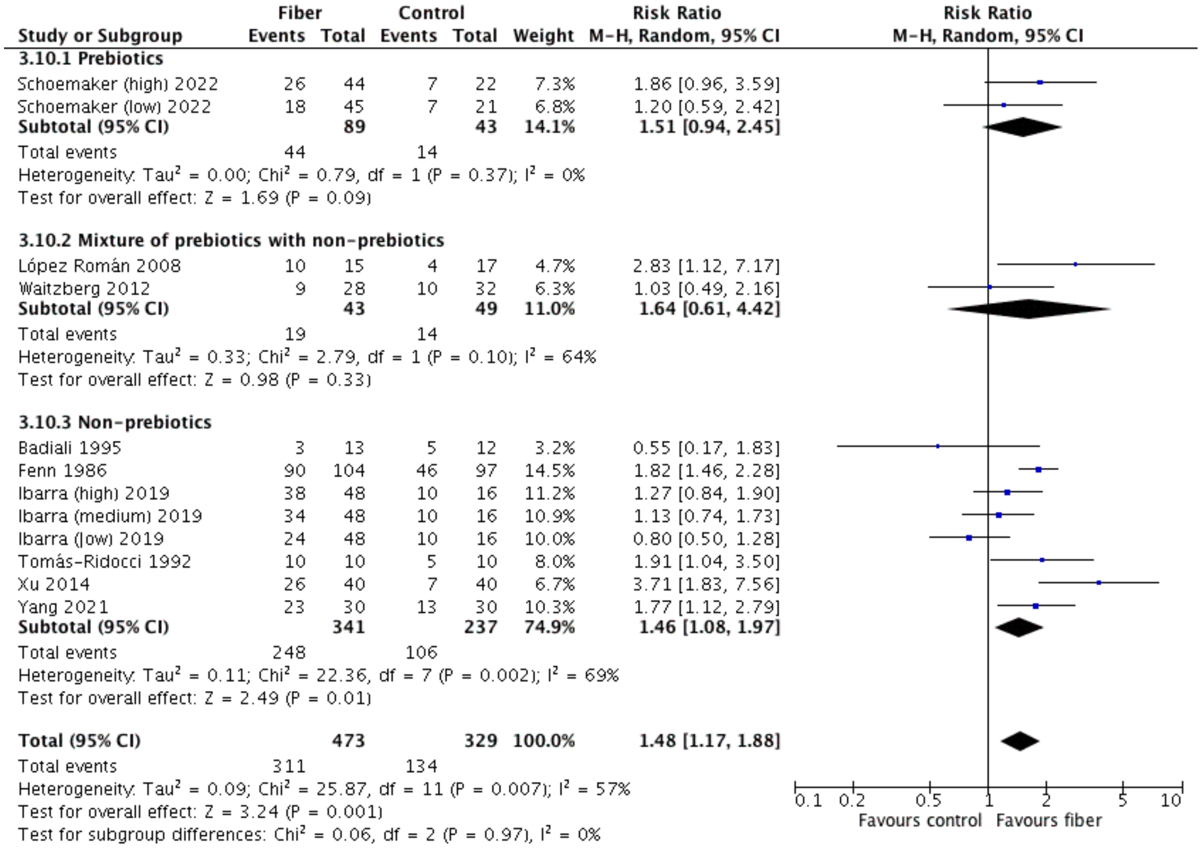

Supplementary Figure 1B Forest plot of subgroup analysis based on prebiotic status of the intervention for response to treatment in randomized controlled trials comparing fiber with control in adults with chronic constipation (n=802). Values are calculated as risk ratio (95% CIs) for response to treatment using a random-effects model. Abbreviations: M-H, Mantel-Haenszel; RR, risk ratio.

Study or Subgroup	Fiber Events	Fiber Total	Control Events	Control Total	Weight	Risk Ratio M-H, Random, 95% CI
**5.5.1 Low dose (≤10 g/d)**						
Ibarra (medium) 2019	34	48	10	16	10.9%	1.13 [0.74, 1.73]
Ibarra (low) 2019	24	48	10	16	10.0%	0.80 [0.50, 1.28]
Schoemaker (low) 2022	18	45	7	21	6.8%	1.20 [0.59, 2.42]
**Subtotal (95% CI)**		**141**		**53**	**27.7%**	**1.01 [0.76, 1.34]**
Total events	76		27			
Heterogeneity: Tau² = 0.00; Chi² = 1.46, df = 2 (P = 0.48); I² = 0%						
Test for overall effect: Z = 0.05 (P = 0.96)						
**5.5.2 High dose (>10 g/d)**						
Badiali 1995	3	13	5	12	3.2%	0.55 [0.17, 1.83]
Fenn 1986	90	104	46	97	14.5%	1.82 [1.46, 2.28]
Ibarra (high) 2019	38	48	10	16	11.2%	1.27 [0.84, 1.90]
López Román 2008	10	15	4	17	4.7%	2.83 [1.12, 7.17]
Schoemaker (high) 2022	26	44	7	22	7.3%	1.86 [0.96, 3.59]
Tomás-Ridocci 1992	10	10	5	10	8.0%	1.91 [1.04, 3.50]
Waitzberg 2012	9	28	10	32	6.3%	1.03 [0.49, 2.16]
Xu 2014	26	40	7	40	6.7%	3.71 [1.83, 7.56]
Yang 2021	23	30	13	30	10.3%	1.77 [1.12, 2.79]
**Subtotal (95% CI)**		**332**		**276**	**72.3%**	**1.72 [1.35, 2.18]**
Total events	235		107			
Heterogeneity: Tau² = 0.05; Chi² = 13.62, df = 8 (P = 0.09); I² = 41%						
Test for overall effect: Z = 4.41 (P < 0.0001)						
**Total (95% CI)**		**473**		**329**	**100.0%**	**1.48 [1.17, 1.88]**
Total events	311		134			
Heterogeneity: Tau² = 0.09; Chi² = 25.87, df = 11 (P = 0.007); I² = 57%						
Test for overall effect: Z = 3.24 (P = 0.001)						
Test for subgroup differences: Chi² = 7.81, df = 1 (P = 0.005), I² = 87.2%						

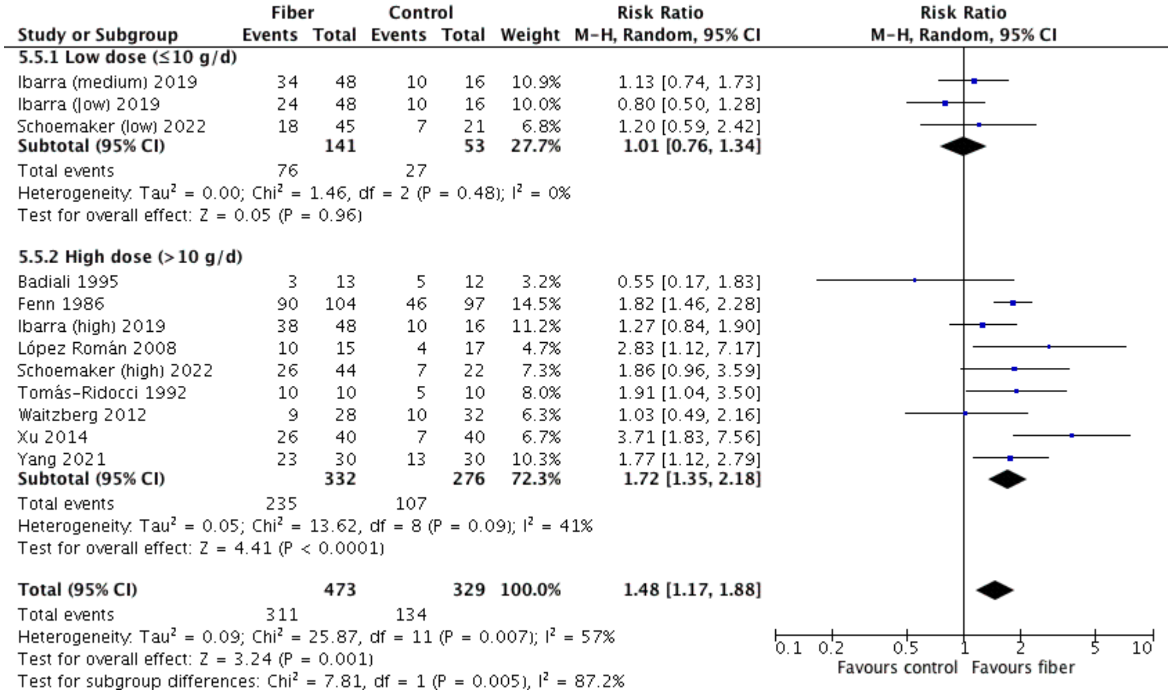

Supplementary Figure 1C Forest plot of subgroup analysis based on the dose administered for response to treatment in randomized controlled trials comparing fiber with control in adults with chronic constipation (n=802). Values are calculated as risk ratio (95% CIs) for response to treatment using a random-effects model. Abbreviations: M-H, Mantel-Haenszel; RR, risk ratio.

Study or Subgroup	Fiber Events	Fiber Total	Control Events	Control Total	Weight	Risk Ratio M-H, Random, 95% CI
**4.8.1 Duration of treatment < 4 weeks**						
Fenn 1986	90	104	46	97	14.5%	1.82 [1.46, 2.28]
Ibarra (high) 2019	38	48	10	16	11.2%	1.27 [0.84, 1.90]
Ibarra (medium) 2019	34	48	10	16	10.9%	1.13 [0.74, 1.73]
Ibarra (low) 2019	24	48	10	16	10.0%	0.80 [0.50, 1.28]
López Román 2008	10	15	4	17	4.7%	2.83 [1.12, 7.17]
Schoemaker (high) 2022	26	44	7	22	7.3%	1.86 [0.96, 3.59]
Schoemaker (low) 2022	18	45	7	21	6.8%	1.20 [0.59, 2.42]
Waitzberg 2012	9	28	10	32	6.3%	1.03 [0.49, 2.16]
**Subtotal (95% CI)**		**380**		**237**	**71.8%**	**1.35 [1.04, 1.75]**
Total events	249		104			
Heterogeneity: Tau^2 = 0.07; Chi^2 = 15.57, df = 7 (P = 0.03); I^2 = 55%						
Test for overall effect: Z = 2.23 (P = 0.03)						
**4.8.2 Duration of treatment ≥ 4 weeks**						
Badiali 1995	3	13	5	12	3.2%	0.55 [0.17, 1.83]
Tomás-Ridocci 1992	10	10	5	10	8.0%	1.91 [1.04, 3.50]
Xu 2014	26	40	7	40	6.7%	3.71 [1.83, 7.56]
Yang 2021	23	30	13	30	10.3%	1.77 [1.12, 2.79]
**Subtotal (95% CI)**		**93**		**92**	**28.2%**	**1.85 [1.07, 3.18]**
Total events	62		30			
Heterogeneity: Tau^2 = 0.18; Chi^2 = 7.70, df = 3 (P = 0.05); I^2 = 61%						
Test for overall effect: Z = 2.21 (P = 0.03)						
**Total (95% CI)**		**473**		**329**	**100.0%**	**1.48 [1.17, 1.88]**
Total events	311		134			
Heterogeneity: Tau^2 = 0.09; Chi^2 = 25.87, df = 11 (P = 0.007); I^2 = 57%						
Test for overall effect: Z = 3.24 (P = 0.001)						
Test for subgroup differences: Chi^2 = 1.05, df = 1 (P = 0.31), I^2 = 4.7%						

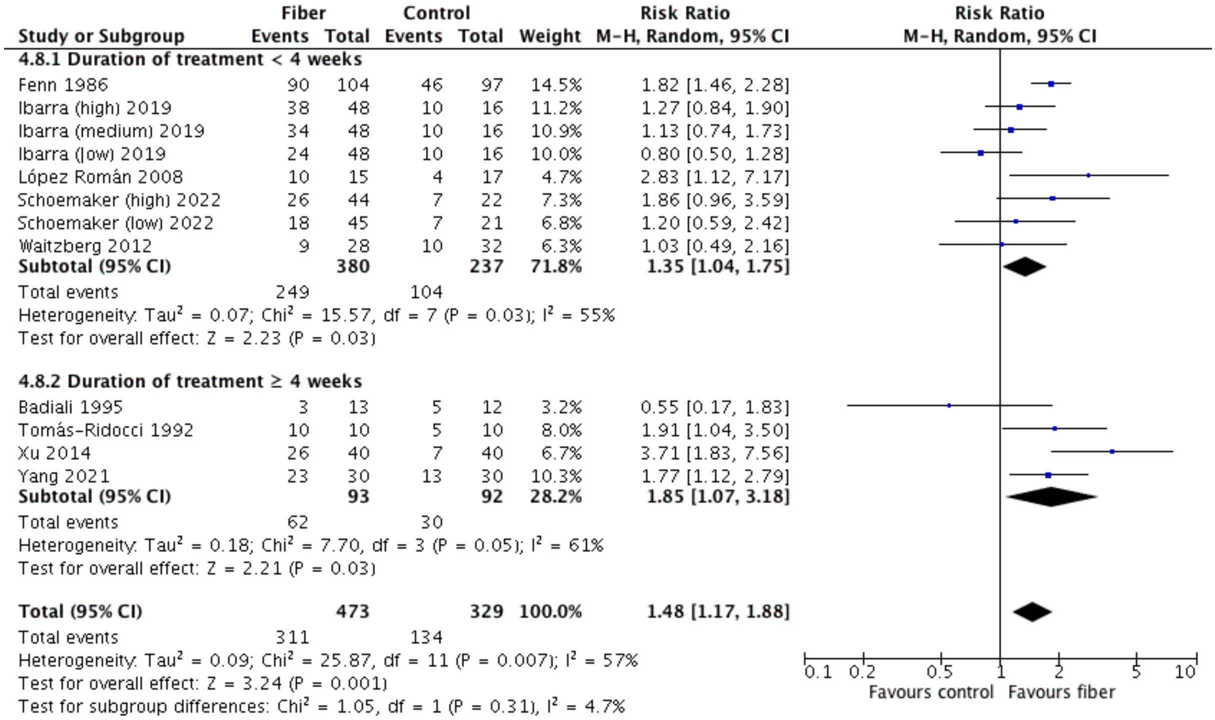

Supplementary Figure 1D Forest plot of subgroup analysis based on treatment duration for response to treatment in randomized controlled trials comparing fiber with control in adults with chronic constipation (n=802). Values are calculated as risk ratio (95% CIs) for response to treatment using a random-effects model. Abbreviations: Mantel-Haenszel; RR, risk ratio.

Stool frequency

Study or Subgroup	Std. Mean Difference	SE	Weight	Std. Mean Difference IV, Random, 95% CI	Risk of Bias A	B	C	D	E	F
Ashraf 1995	0.6325	0.44	4.9%	0.63 [−0.23, 1.49]	?	?	−	+	?	−
Badiali 1995	2.0066	0.5	4.5%	2.01 [1.03, 2.99]	?	−	−	+	?	−
Duncan (high) 2018	0.0566	0.29	5.8%	0.06 [−0.51, 0.62]	?	+	+	+	?	?
Duncan (low) 2018	0.054	0.29	5.8%	0.05 [−0.51, 0.62]	?	+	+	+	?	?
Fenn 1986	0.8843	0.16	6.4%	0.88 [0.57, 1.20]	?	−	−	+	?	−
Glibowski 2020	1.3559	0.5	4.5%	1.36 [0.38, 2.34]	?	?	−	+	?	−
Huh 2007	1.0475	0.33	5.6%	1.05 [0.40, 1.69]	?	−	−	+	?	−
Ibarra (high) 2019	0.1553	0.29	5.8%	0.16 [−0.41, 0.72]	+	+	+	+	?	?
Ibarra (medium) 2019	−0.0469	0.29	5.8%	−0.05 [−0.62, 0.52]	+	+	+	+	?	?
Ibarra (low) 2019	−0.3454	0.29	5.8%	−0.35 [−0.91, 0.22]	+	+	+	+	?	?
Li 2017	1.27	0.22	6.2%	1.27 [0.84, 1.70]	?	+	+	+	?	?
López Román 2008	1.0196	0.38	5.3%	1.02 [0.27, 1.76]	?	−	−	−	−	−
Micka 2017	0.3694	0.22	6.2%	0.37 [−0.06, 0.80]	+	−	?	+	?	−
Schoemaker (high) 2022	0.488	0.26	6.0%	0.49 [−0.02, 1.00]	?	+	+	+	+	?
Schoemaker (low) 2022	0.0625	0.26	6.0%	0.06 [−0.45, 0.57]	?	+	+	+	+	?
Tomás-Ridocci 1992	2.2886	0.58	4.1%	2.29 [1.15, 3.43]	?	+	−	+	?	−
Waitzberg 2012	−0.2297	0.3	5.7%	−0.23 [−0.82, 0.36]	?	−	−	+	?	−
Xu 2014	2.8387	0.32	5.6%	2.84 [2.21, 3.47]	?	+	+	+	?	?
**Total (95% CI)**			**100.0%**	**0.72 [0.36, 1.08]**						

Heterogeneity: Tau² = 0.51; Chi² = 121.12, df = 17 (P < 0.00001); I² = 86%
Test for overall effect: Z = 3.88 (P = 0.0001)

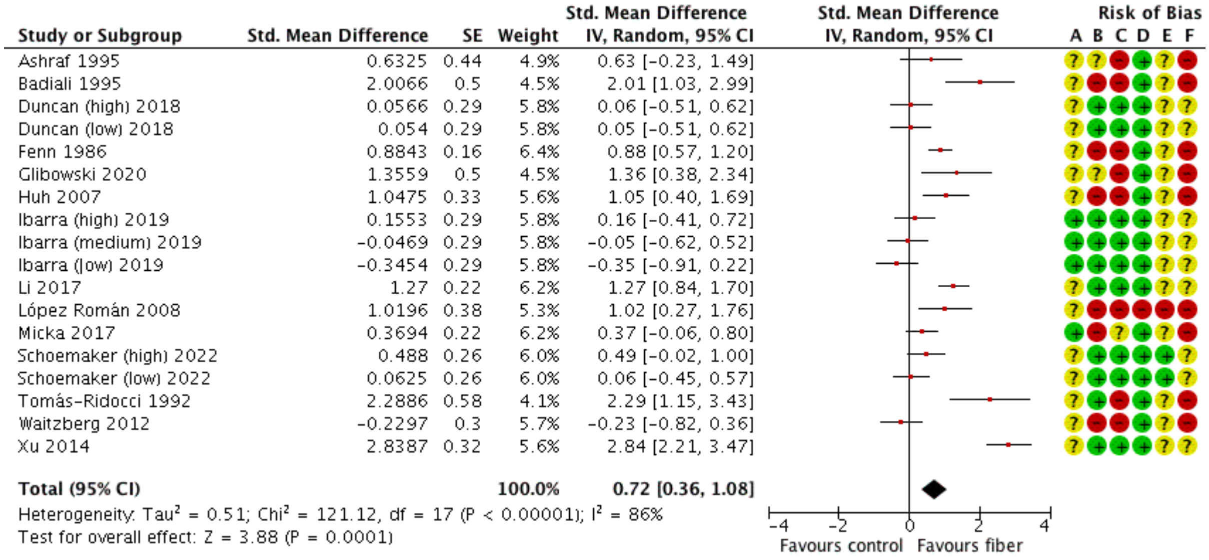

Supplementary Figure 2A Forest plot of stool frequency in randomized controlled trials comparing fiber with control in adults with chronic constipation (n=1040). Values were calculated as standardized mean difference (95% CIs) using a random-effects model. Risk of bias key: A=bias arising from the randomization process; B=bias arising from deviations from intended interventions; C=bias due to missing outcome data; D=bias in measurement of the outcome; E=bias in selection of the reported result; F=overall risk of bias. Abbreviation: IV, inverse variance.

Study or Subgroup	Std. Mean Difference	SE	Weight	Std. Mean Difference IV, Random, 95% CI
**3.3.1 Prebiotics**				
Glibowski 2020	1.3559	0.5	4.5%	1.36 [0.38, 2.34]
Li 2017	1.27	0.22	6.2%	1.27 [0.84, 1.70]
Micka 2017	0.3694	0.22	6.2%	0.37 [−0.06, 0.80]
Schoemaker (high) 2022	0.488	0.26	6.0%	0.49 [−0.02, 1.00]
Schoemaker (low) 2022	0.0625	0.26	6.0%	0.06 [−0.45, 0.57]
**Subtotal (95% CI)**			**28.8%**	**0.66 [0.17, 1.15]**
Heterogeneity: $Tau^2 = 0.23$; $Chi^2 = 17.04$, df = 4 (P = 0.002); $I^2 = 77\%$				
Test for overall effect: Z = 2.65 (P = 0.008)				
**3.3.2 Mixture of prebiotics with non-prebiotics**				
López Román 2008	1.0196	0.38	5.3%	1.02 [0.27, 1.76]
Waitzberg 2012	−0.2297	0.3	5.7%	−0.23 [−0.82, 0.36]
**Subtotal (95% CI)**			**11.0%**	**0.37 [−0.85, 1.60]**
Heterogeneity: $Tau^2 = 0.66$; $Chi^2 = 6.66$, df = 1 (P = 0.010); $I^2 = 85\%$				
Test for overall effect: Z = 0.60 (P = 0.55)				
**3.3.3 Non-prebiotics**				
Ashraf 1995	0.6325	0.44	4.9%	0.63 [−0.23, 1.49]
Badiali 1995	2.0066	0.5	4.5%	2.01 [1.03, 2.99]
Duncan (high) 2018	0.0566	0.29	5.8%	0.06 [−0.51, 0.62]
Duncan (low) 2018	0.054	0.29	5.8%	0.05 [−0.51, 0.62]
Fenn 1986	0.8843	0.16	6.4%	0.88 [0.57, 1.20]
Ibarra (high) 2019	0.1553	0.29	5.8%	0.16 [−0.41, 0.72]
Ibarra (medium) 2019	−0.0469	0.29	5.8%	−0.05 [−0.62, 0.52]
Ibarra (low) 2019	−0.3454	0.29	5.8%	−0.35 [−0.91, 0.22]
Tomás-Ridocci 1992	2.2886	0.58	4.1%	2.29 [1.15, 3.43]
Xu 2014	2.8387	0.32	5.6%	2.84 [2.21, 3.47]
**Subtotal (95% CI)**			**54.6%**	**0.80 [0.19, 1.41]**
Heterogeneity: $Tau^2 = 0.84$; $Chi^2 = 93.14$, df = 9 (P < 0.00001); $I^2 = 90\%$				
Test for overall effect: Z = 2.58 (P = 0.010)				
**3.3.4 Unspecified**				
Huh 2007	1.0475	0.33	5.6%	1.05 [0.40, 1.69]
**Subtotal (95% CI)**			**5.6%**	**1.05 [0.40, 1.69]**
Heterogeneity: Not applicable				
Test for overall effect: Z = 3.17 (P = 0.002)				
**Total (95% CI)**			**100.0%**	**0.72 [0.36, 1.08]**
Heterogeneity: $Tau^2 = 0.51$; $Chi^2 = 121.12$, df = 17 (P < 0.00001); $I^2 = 86\%$				
Test for overall effect: Z = 3.88 (P = 0.0001)				
Test for subgroup differences: $Chi^2 = 1.31$, df = 3 (P = 0.73), $I^2 = 0\%$				

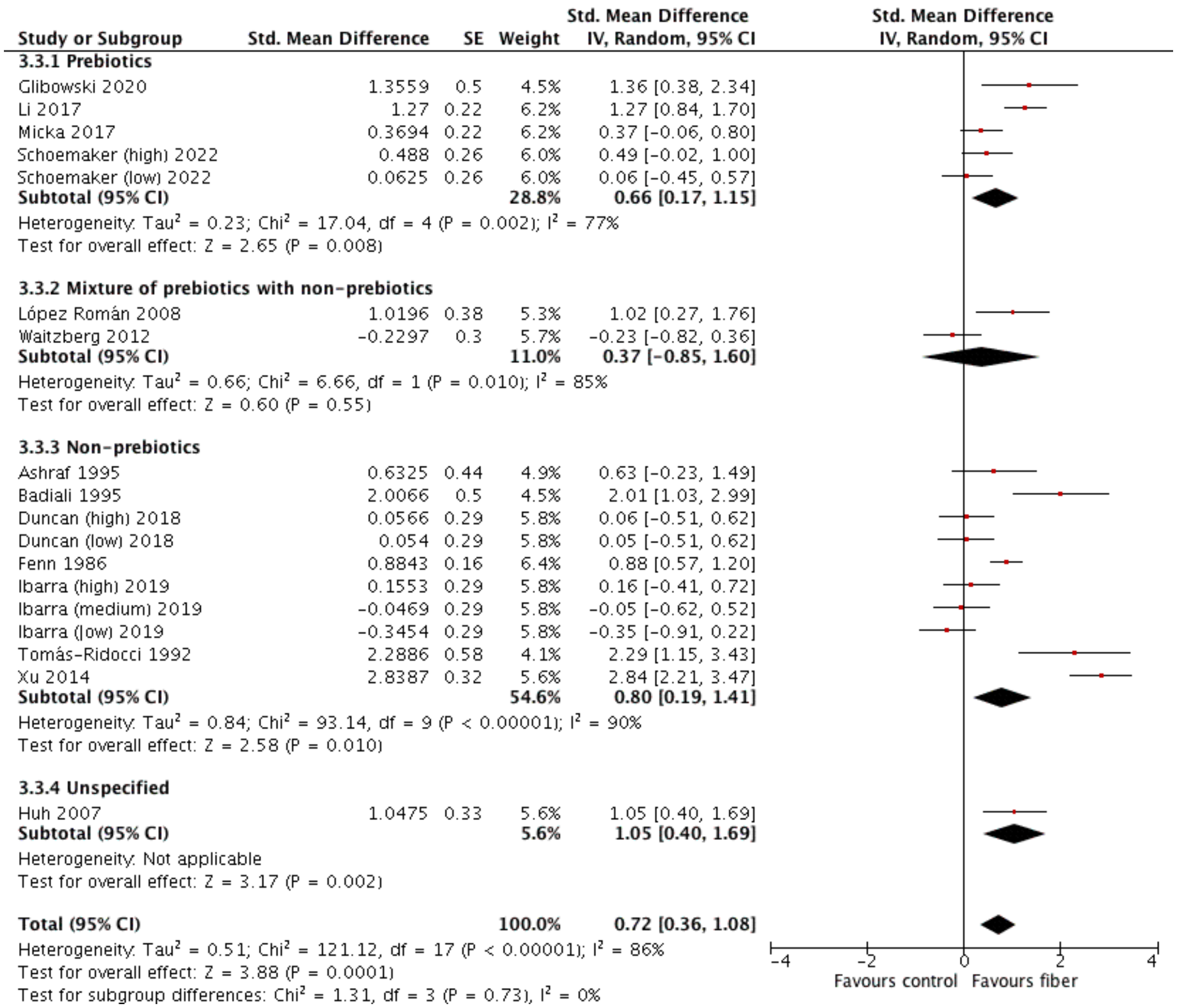

Supplementary Figure 2B Forest plot of subgroup analysis based on prebiotic status of the intervention for stool frequency in randomized controlled trials comparing fiber with control in adults with chronic constipation (n=1040). Values were calculated as standardized mean difference (95% CIs) using a random-effects model. Abbreviation: IV, inverse variance.

Study or Subgroup	Std. Mean Difference	SE	Weight	Std. Mean Difference IV, Random, 95% CI
**5.3.1 Low dose (≤10 g/d)**				
Duncan (low) 2018	0.054	0.29	5.8%	0.05 [−0.51, 0.62]
Ibarra (medium) 2019	−0.0469	0.29	5.8%	−0.05 [−0.62, 0.52]
Ibarra (low) 2019	−0.3454	0.29	5.8%	−0.35 [−0.91, 0.22]
Li 2017	1.27	0.22	6.2%	1.27 [0.84, 1.70]
Schoemaker (low) 2022	0.0625	0.26	6.0%	0.06 [−0.45, 0.57]
**Subtotal (95% CI)**			**29.6%**	**0.21 [−0.40, 0.83]**
Heterogeneity: Tau² = 0.42; Chi² = 27.33, df = 4 (P < 0.0001); I² = 85%				
Test for overall effect: Z = 0.68 (P = 0.49)				
**5.3.2 High dose (>10 g/d)**				
Ashraf 1995	0.6325	0.44	4.9%	0.63 [−0.23, 1.49]
Badiali 1995	2.0066	0.5	4.5%	2.01 [1.03, 2.99]
Duncan (high) 2018	0.0566	0.29	5.8%	0.06 [−0.51, 0.62]
Fenn 1986	0.8843	0.16	6.4%	0.88 [0.57, 1.20]
Glibowski 2020	1.3559	0.5	4.5%	1.36 [0.38, 2.34]
Huh 2007	1.0475	0.33	5.6%	1.05 [0.40, 1.69]
Ibarra (high) 2019	0.1553	0.29	5.8%	0.16 [−0.41, 0.72]
López Román 2008	1.0196	0.38	5.3%	1.02 [0.27, 1.76]
Micka 2017	0.3694	0.22	6.2%	0.37 [−0.06, 0.80]
Schoemaker (high) 2022	0.488	0.26	6.0%	0.49 [−0.02, 1.00]
Tomás-Ridocci 1992	2.2886	0.58	4.1%	2.29 [1.15, 3.43]
Waitzberg 2012	−0.2297	0.3	5.7%	−0.23 [−0.82, 0.36]
Xu 2014	2.8387	0.32	5.6%	2.84 [2.21, 3.47]
**Subtotal (95% CI)**			**70.4%**	**0.93 [0.49, 1.38]**
Heterogeneity: Tau² = 0.54; Chi² = 84.02, df = 12 (P < 0.00001); I² = 86%				
Test for overall effect: Z = 4.12 (P < 0.0001)				
**Total (95% CI)**			**100.0%**	**0.72 [0.36, 1.08]**
Heterogeneity: Tau² = 0.51; Chi² = 121.12, df = 17 (P < 0.00001); I² = 86%				
Test for overall effect: Z = 3.88 (P = 0.0001)				
Test for subgroup differences: Chi² = 3.48, df = 1 (P = 0.06), I² = 71.2%				

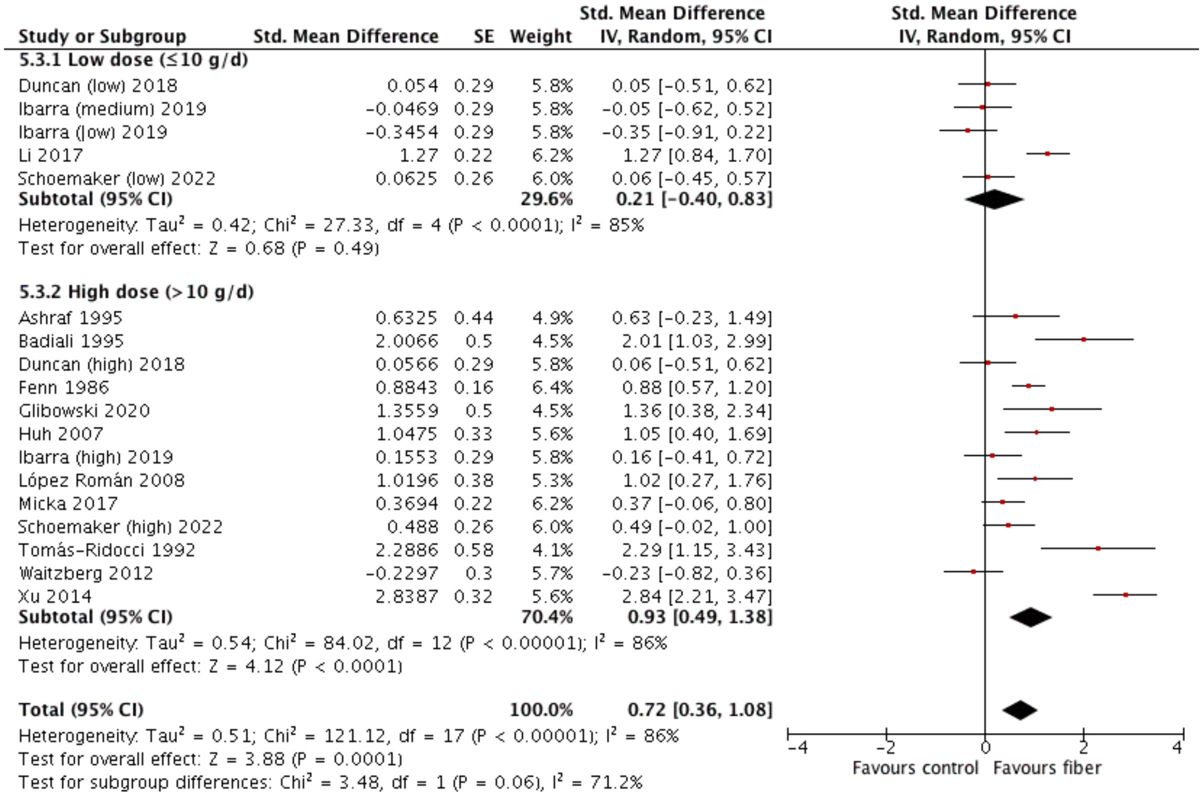

Supplementary Figure 2C Forest plot of subgroup analysis based on the dose administered for stool frequency in randomized controlled trials comparing fiber with control in adults with chronic constipation (n=1040). Values were calculated as standardized mean difference (95% CIs) using a random-effects model. Abbreviation: IV, inverse variance.

Study or Subgroup	Std. Mean Difference	SE	Weight	Std. Mean Difference IV, Random, 95% CI
**4.3.1 Duration of treatment < 4 weeks**				
Fenn 1986	0.8843	0.16	6.4%	0.88 [0.57, 1.20]
Glibowski 2020	1.3559	0.5	4.5%	1.36 [0.38, 2.34]
Ibarra (high) 2019	0.1553	0.29	5.8%	0.16 [−0.41, 0.72]
Ibarra (medium) 2019	−0.0469	0.29	5.8%	−0.05 [−0.62, 0.52]
Ibarra (low) 2019	−0.3454	0.29	5.8%	−0.35 [−0.91, 0.22]
López Román 2008	1.0196	0.38	5.3%	1.02 [0.27, 1.76]
Schoemaker (high) 2022	0.488	0.26	6.0%	0.49 [−0.02, 1.00]
Schoemaker (low) 2022	0.0625	0.26	6.0%	0.06 [−0.45, 0.57]
Waitzberg 2012	−0.2297	0.3	5.7%	−0.23 [−0.82, 0.36]
**Subtotal (95% CI)**			**51.3%**	**0.33 [−0.03, 0.70]**
Heterogeneity: $Tau^2 = 0.22$; $Chi^2 = 31.37$, df = 8 (P = 0.0001); $I^2 = 74\%$				
Test for overall effect: Z = 1.80 (P = 0.07)				
**4.3.2 Duration of treatment ≥ 4 weeks**				
Ashraf 1995	0.6325	0.44	4.9%	0.63 [−0.23, 1.49]
Badiali 1995	2.0066	0.5	4.5%	2.01 [1.03, 2.99]
Duncan (high) 2018	0.0566	0.29	5.8%	0.06 [−0.51, 0.62]
Duncan (low) 2018	0.054	0.29	5.8%	0.05 [−0.51, 0.62]
Huh 2007	1.0475	0.33	5.6%	1.05 [0.40, 1.69]
Li 2017	1.27	0.22	6.2%	1.27 [0.84, 1.70]
Micka 2017	0.3694	0.22	6.2%	0.37 [−0.06, 0.80]
Tomás-Ridocci 1992	2.2886	0.58	4.1%	2.29 [1.15, 3.43]
Xu 2014	2.8387	0.32	5.6%	2.84 [2.21, 3.47]
**Subtotal (95% CI)**			**48.7%**	**1.13 [0.50, 1.76]**
Heterogeneity: $Tau^2 = 0.80$; $Chi^2 = 73.35$, df = 8 (P < 0.00001); $I^2 = 89\%$				
Test for overall effect: Z = 3.52 (P = 0.0004)				
**Total (95% CI)**			**100.0%**	**0.72 [0.36, 1.08]**
Heterogeneity: $Tau^2 = 0.51$; $Chi^2 = 121.12$, df = 17 (P < 0.00001); $I^2 = 86\%$				
Test for overall effect: Z = 3.88 (P = 0.0001)				
Test for subgroup differences: $Chi^2 = 4.59$, df = 1 (P = 0.03), $I^2 = 78.2\%$				

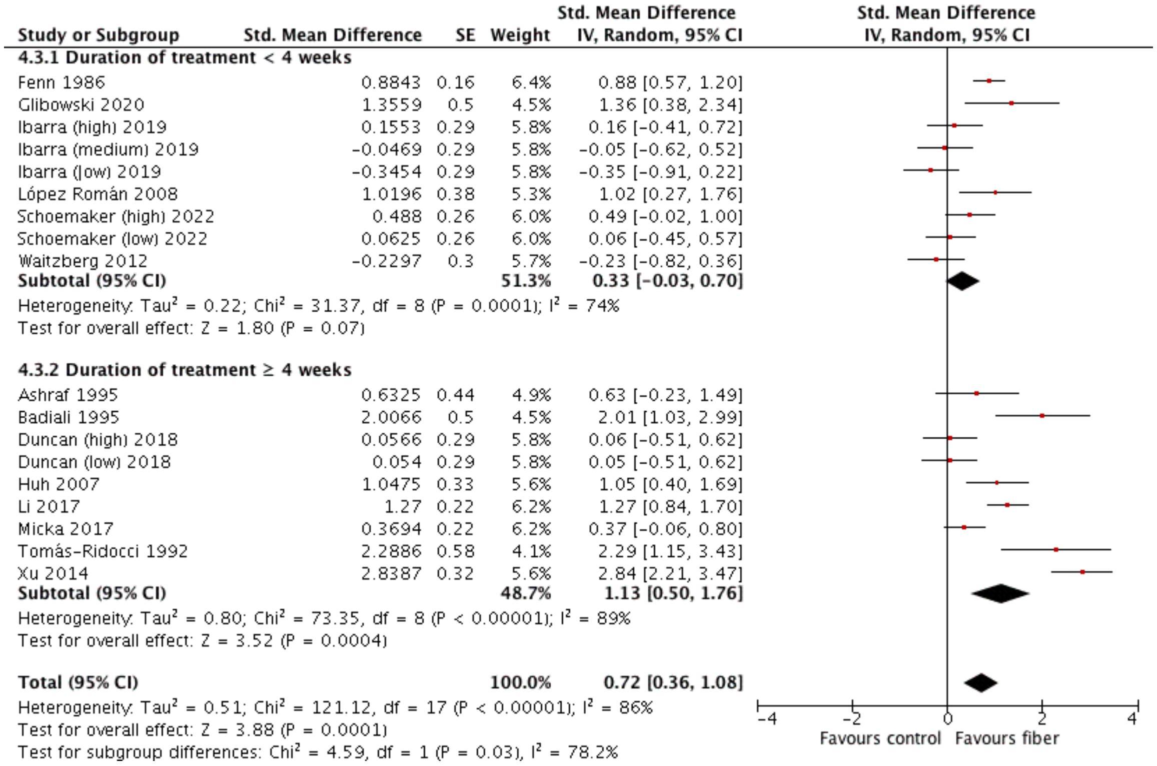

Supplementary Figure 2D Forest plot of subgroup analysis based on the treatment duration for stool frequency in randomized controlled trials comparing fiber with control in adults with chronic constipation (n=1040). Values were calculated as standardized mean difference (95% CIs) using a random-effects model. Abbreviation: IV, inverse variance.

Study or Subgroup	Fiber Mean	Fiber SD	Fiber Total	Control Mean	Control SD	Control Total	Weight	Mean Difference IV, Random, 95% CI
**2.3.1 Polydextrose**								
Duncan (high) 2018	−0.6	3.3	34	0.1	2.7	18	5.6%	−0.70 [−2.37, 0.97]
Duncan (low) 2018	0.8	2.6	34	0.1	2.7	18	6.0%	0.70 [−0.82, 2.22]
Ibarra (high) 2019	9.6	5.2	48	8.8	5	16	3.0%	0.80 [−2.06, 3.66]
Ibarra (medium) 2019	8.6	4	48	8.8	5	16	3.3%	−0.20 [−2.90, 2.50]
Ibarra (low) 2019	7.4	3.7	48	8.8	5	16	3.3%	−1.40 [−4.06, 1.26]
**Subtotal (95% CI)**			**212**			**84**	**21.2%**	**−0.06 [−0.98, 0.85]**
Heterogeneity: Tau^2 = 0.00; Chi^2 = 2.85, df = 4 (P = 0.58); I^2 = 0%								
Test for overall effect: Z = 0.14 (P = 0.89)								
**2.3.2 Psyllium**								
Ashraf 1995	3.8	1.33	11	3	1.33	11	7.4%	0.80 [−0.31, 1.91]
Fenn 1986	10.38	5.58	91	6.38	2.98	84	6.7%	4.00 [2.69, 5.31]
Tomás-Ridocci 1992	8	2.2	10	3.4	1.8	10	5.3%	4.60 [2.84, 6.36]
**Subtotal (95% CI)**			**112**			**105**	**19.4%**	**3.08 [0.61, 5.54]**
Heterogeneity: Tau^2 = 4.22; Chi^2 = 19.40, df = 2 (P < 0.0001); I^2 = 90%								
Test for overall effect: Z = 2.45 (P = 0.01)								
**2.3.3 Inulin-type fructans**								
Glibowski 2020	12.88	1.62	10	9.13	3.56	10	3.8%	3.75 [1.33, 6.17]
Micka 2017	3.67	1.53	44	3.17	1.15	44	9.1%	0.50 [−0.07, 1.07]
**Subtotal (95% CI)**			**54**			**54**	**12.9%**	**1.90 [−1.25, 5.06]**
Heterogeneity: Tau^2 = 4.47; Chi^2 = 6.55, df = 1 (P = 0.01); I^2 = 85%								
Test for overall effect: Z = 1.18 (P = 0.24)								
**2.3.4 Mixture of inulin with partially hydrolysed guar gum**								
Waitzberg 2012	5.95	2.5	21	6.7	3.83	23	5.0%	−0.75 [−2.65, 1.15]
**Subtotal (95% CI)**			**21**			**23**	**5.0%**	**−0.75 [−2.65, 1.15]**
Heterogeneity: Not applicable								
Test for overall effect: Z = 0.78 (P = 0.44)								
**2.3.5 Galacto-oligosaccharides**								
Li 2017	3.02	1.15	52	1.86	0.58	51	9.6%	1.16 [0.81, 1.51]
Schoemaker (high) 2022	4.3	1	44	3.7	1.6	22	8.6%	0.60 [−0.13, 1.33]
Schoemaker (low) 2022	3.8	1.6	45	3.7	1.6	21	8.3%	0.10 [−0.73, 0.93]
**Subtotal (95% CI)**			**141**			**94**	**26.5%**	**0.71 [0.07, 1.34]**
Heterogeneity: Tau^2 = 0.21; Chi^2 = 6.29, df = 2 (P = 0.04); I^2 = 68%								
Test for overall effect: Z = 2.17 (P = 0.03)								
**2.3.7 Wheat bran**								
Badiali 1995	7.2	2.2	12	3.5	1.4	12	6.2%	3.70 [2.22, 5.18]
**Subtotal (95% CI)**			**12**			**12**	**6.2%**	**3.70 [2.22, 5.18]**
Heterogeneity: Not applicable								
Test for overall effect: Z = 4.92 (P < 0.00001)								
**2.3.8 Unspecified**								
Huh 2007	5.18	0.56	22	4.13	1.33	20	8.9%	1.05 [0.42, 1.68]
**Subtotal (95% CI)**			**22**			**20**	**8.9%**	**1.05 [0.42, 1.68]**
Heterogeneity: Not applicable								
Test for overall effect: Z = 3.28 (P = 0.001)								
**Total (95% CI)**			**574**			**392**	**100.0%**	**1.19 [0.59, 1.78]**
Heterogeneity: Tau^2 = 0.94; Chi^2 = 74.83, df = 15 (P < 0.00001); I^2 = 80%								
Test for overall effect: Z = 3.90 (P < 0.0001)								
Test for subgroup differences: Chi^2 = 24.95, df = 6 (P = 0.0003), I^2 = 76.0%								

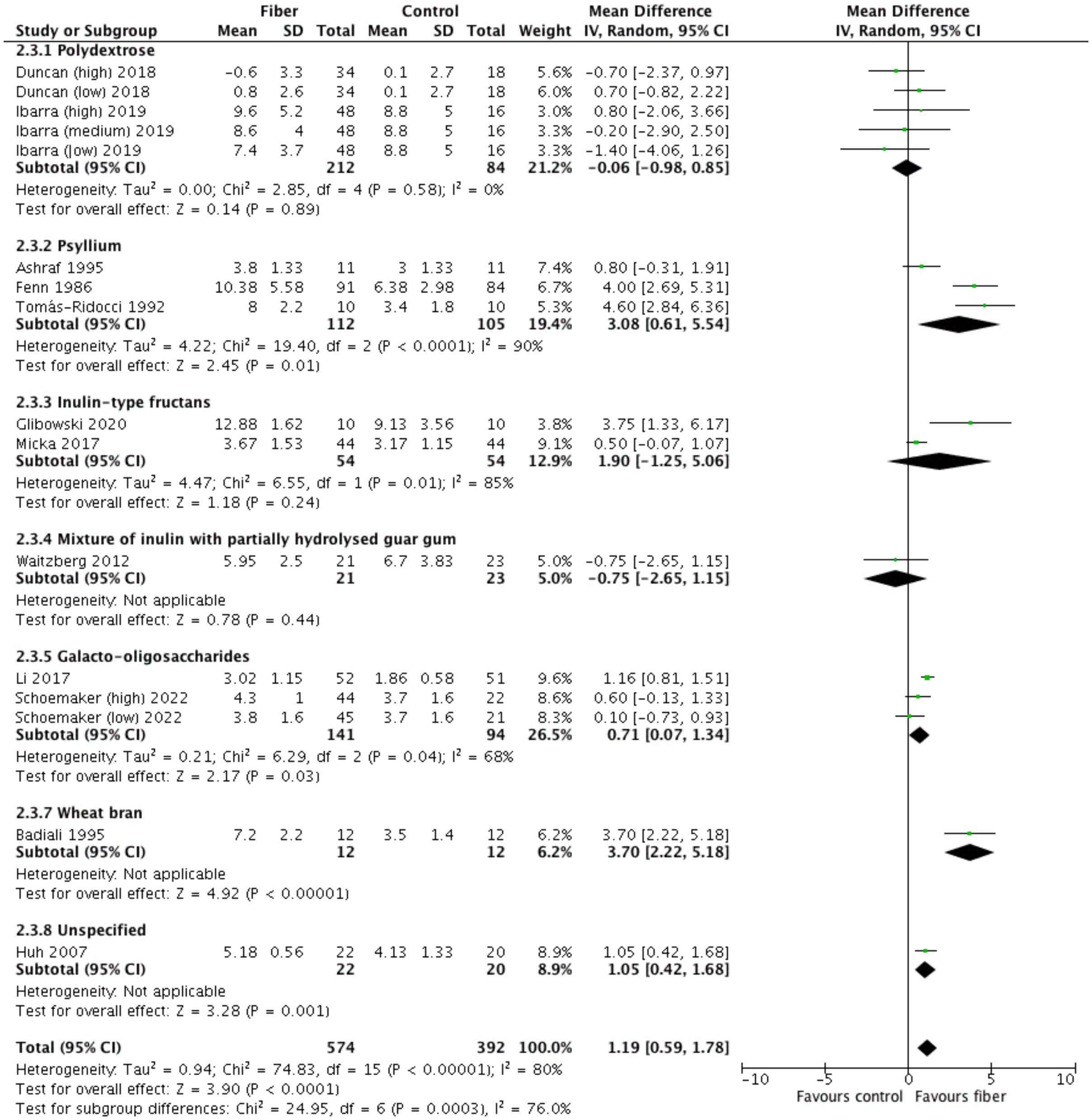

Supplementary Figure 3A Forest plot of subgroup analysis based on type of fiber for stool frequency (bowel movements/week) in randomized controlled trials comparing fiber with control in adults with chronic constipation (n=966). Values were calculated as mean difference (95% CIs) using a random-effects model. Abbreviation: IV, inverse variance.

Study or Subgroup	Fiber Mean	Fiber SD	Fiber Total	Control Mean	Control SD	Control Total	Weight	Mean Difference IV, Random, 95% CI
**3.2.1 Prebiotics**								
Glibowski 2020	12.88	1.62	10	9.13	3.56	10	3.8%	3.75 [1.33, 6.17]
Li 2017	3.02	1.15	52	1.86	0.58	51	9.6%	1.16 [0.81, 1.51]
Micka 2017	3.67	1.53	44	3.17	1.15	44	9.1%	0.50 [−0.07, 1.07]
Schoemaker (high) 2022	4.3	1	44	3.7	1.6	22	8.6%	0.60 [−0.13, 1.33]
Schoemaker (low) 2022	3.8	1.6	45	3.7	1.6	21	8.3%	0.10 [−0.73, 0.93]
**Subtotal (95% CI)**			**195**			**148**	**39.4%**	**0.79 [0.21, 1.37]**
Heterogeneity: Tau² = 0.27; Chi² = 13.53, df = 4 (P = 0.009); I² = 70%								
Test for overall effect: Z = 2.68 (P = 0.007)								
**3.2.2 Mixture of prebiotics with non−prebiotics**								
Waitzberg 2012	5.95	2.5	21	6.7	3.83	23	5.0%	−0.75 [−2.65, 1.15]
**Subtotal (95% CI)**			**21**			**23**	**5.0%**	**−0.75 [−2.65, 1.15]**
Heterogeneity: Not applicable								
Test for overall effect: Z = 0.78 (P = 0.44)								
**3.2.3 Non−prebiotics**								
Ashraf 1995	3.8	1.33	11	3	1.33	11	7.4%	0.80 [−0.31, 1.91]
Badiali 1995	7.2	2.2	12	3.5	1.4	12	6.2%	3.70 [2.22, 5.18]
Duncan (high) 2018	−0.6	3.3	34	0.1	2.7	18	5.6%	−0.70 [−2.37, 0.97]
Duncan (low) 2018	0.8	2.6	34	0.1	2.7	18	6.0%	0.70 [−0.82, 2.22]
Fenn 1986	10.38	5.58	91	6.38	2.98	84	6.7%	4.00 [2.69, 5.31]
Ibarra (high) 2019	9.6	5.2	48	8.8	5	16	3.0%	0.80 [−2.06, 3.66]
Ibarra (medium) 2019	8.6	4	48	8.8	5	16	3.3%	−0.20 [−2.90, 2.50]
Ibarra (low) 2019	7.4	3.7	48	8.8	5	16	3.3%	−1.40 [−4.06, 1.26]
Tomás−Ridocci 1992	8	2.2	10	3.4	1.8	10	5.3%	4.60 [2.84, 6.36]
**Subtotal (95% CI)**			**336**			**201**	**46.8%**	**1.50 [0.09, 2.91]**
Heterogeneity: Tau² = 3.70; Chi² = 48.73, df = 8 (P < 0.00001); I² = 84%								
Test for overall effect: Z = 2.09 (P = 0.04)								
**3.2.4 Unspecified**								
Huh 2007	5.18	0.56	22	4.13	1.33	20	8.9%	1.05 [0.42, 1.68]
**Subtotal (95% CI)**			**22**			**20**	**8.9%**	**1.05 [0.42, 1.68]**
Heterogeneity: Not applicable								
Test for overall effect: Z = 3.28 (P = 0.001)								
**Total (95% CI)**			**574**			**392**	**100.0%**	**1.19 [0.59, 1.78]**
Heterogeneity: Tau² = 0.94; Chi² = 74.83, df = 15 (P < 0.00001); I² = 80%								
Test for overall effect: Z = 3.90 (P < 0.0001)								
Test for subgroup differences: Chi² = 3.95, df = 3 (P = 0.27), I² = 24.1%								

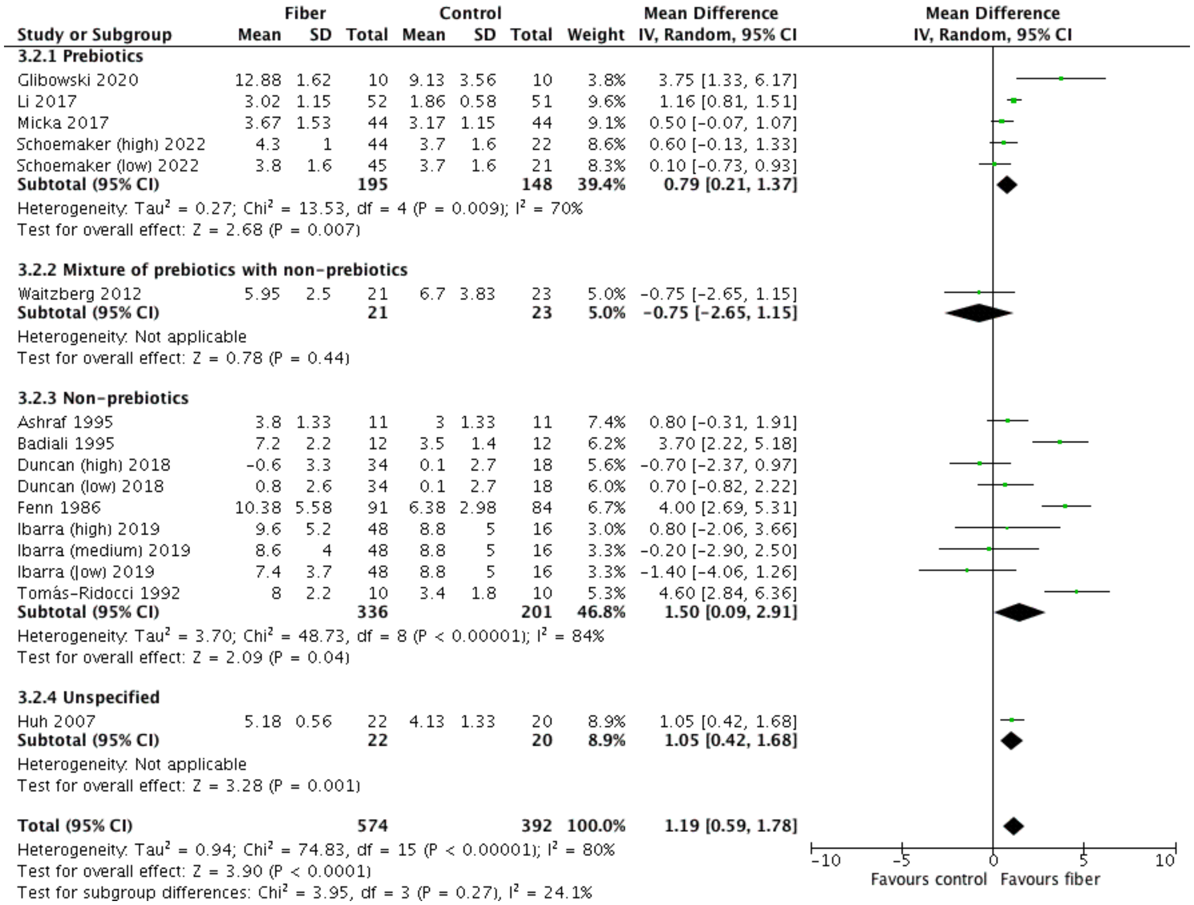

Supplementary Figure 3B Forest plot of subgroup analysis based on prebiotic status of the intervention for stool frequency (bowel movements/week) in randomized controlled trials comparing fiber with control in adults with chronic constipation (n=966). Values were calculated as mean difference (95% CIs) using a random-effects model. Abbreviation: IV, inverse variance.

Study or Subgroup	Fiber Mean	Fiber SD	Fiber Total	Control Mean	Control SD	Control Total	Weight	Mean Difference IV, Random, 95% CI
**5.9.1 Low dose (≤10 g/d)**								
Duncan (low) 2018	0.8	2.6	34	0.1	2.7	18	6.0%	0.70 [−0.82, 2.22]
Ibarra (medium) 2019	8.6	4	48	8.8	5	16	3.3%	−0.20 [−2.90, 2.50]
Ibarra (low) 2019	7.4	3.7	48	8.8	5	16	3.3%	−1.40 [−4.06, 1.26]
Li 2017	3.02	1.15	52	1.86	0.51	51	9.6%	1.16 [0.82, 1.50]
Schoemaker (low) 2022	3.8	1.6	45	3.7	1.6	21	8.3%	0.10 [−0.73, 0.93]
**Subtotal (95% CI)**			**227**			**122**	**30.5%**	**0.49 [−0.30, 1.28]**
Heterogeneity: $Tau^2 = 0.37$; $Chi^2 = 9.27$, df = 4 (P = 0.05); $I^2 = 57\%$								
Test for overall effect: Z = 1.22 (P = 0.22)								
**5.9.2 High dose (>10 g/d)**								
Ashraf 1995	3.8	1.33	11	3	1.33	11	7.4%	0.80 [−0.31, 1.91]
Badiali 1995	7.2	2.2	12	3.5	1.4	12	6.2%	3.70 [2.22, 5.18]
Duncan (high) 2018	−0.6	3.3	34	0.1	2.7	18	5.6%	−0.70 [−2.37, 0.97]
Fenn 1986	10.38	5.58	91	6.38	2.98	84	6.7%	4.00 [2.69, 5.31]
Glibowski 2020	12.88	1.62	10	9.13	3.56	10	3.7%	3.75 [1.33, 6.17]
Huh 2007	5.18	0.56	22	4.13	1.33	20	8.9%	1.05 [0.42, 1.68]
Ibarra (high) 2019	9.6	5.2	48	8.8	5	16	3.0%	0.80 [−2.06, 3.66]
Micka 2017	3.67	1.53	44	3.17	1.15	44	9.1%	0.50 [−0.07, 1.07]
Schoemaker (high) 2022	4.3	1	44	3.7	1.6	22	8.6%	0.60 [−0.13, 1.33]
Tomás-Ridocci 1992	8	2.2	10	3.4	1.8	10	5.3%	4.60 [2.84, 6.36]
Waitzberg 2012	5.95	2.5	21	6.7	3.83	23	4.9%	−0.75 [−2.65, 1.15]
**Subtotal (95% CI)**			**347**			**270**	**69.5%**	**1.60 [0.71, 2.48]**
Heterogeneity: $Tau^2 = 1.66$; $Chi^2 = 65.05$, df = 10 (P < 0.00001); $I^2 = 85\%$								
Test for overall effect: Z = 3.54 (P = 0.0004)								
**Total (95% CI)**			**574**			**392**	**100.0%**	**1.19 [0.59, 1.78]**
Heterogeneity: $Tau^2 = 0.93$; $Chi^2 = 74.86$, df = 15 (P < 0.00001); $I^2 = 80\%$								
Test for overall effect: Z = 3.91 (P < 0.0001)								
Test for subgroup differences: $Chi^2 = 3.35$, df = 1 (P = 0.07), $I^2 = 70.2\%$								

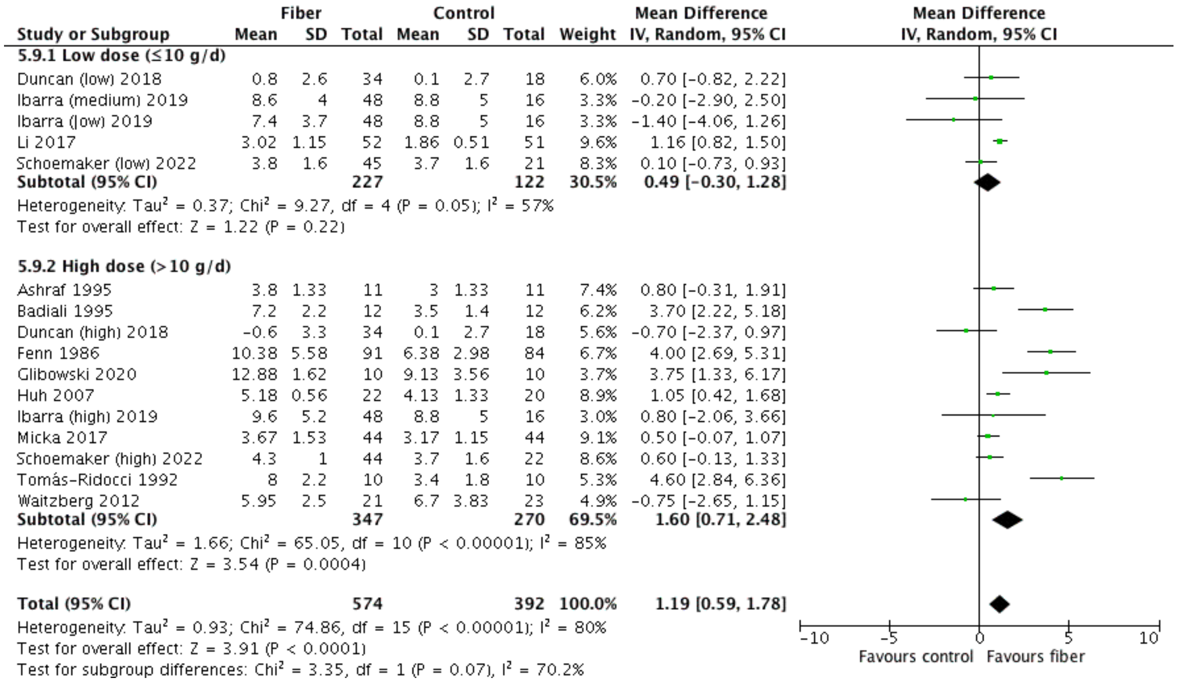

Supplementary Figure 3C Forest plot of subgroup analysis based on the dose administered for stool frequency (bowel movements/week) in randomized controlled trials comparing fiber with control in adults with chronic constipation (n=966). Values were calculated as mean difference (95% CIs) using a random-effects model. Abbreviation: IV, inverse variance.

Study or Subgroup	Fiber Mean	Fiber SD	Fiber Total	Control Mean	Control SD	Control Total	Weight	Mean Difference IV, Random, 95% CI
**4.2.1 Duration of treatment < 4 weeks**								
Fenn 1986	10.38	5.58	91	6.38	2.98	84	6.7%	4.00 [2.69, 5.31]
Glibowski 2020	12.88	1.62	10	9.13	3.56	10	3.8%	3.75 [1.33, 6.17]
Ibarra (high) 2019	9.6	5.2	48	8.8	5	16	3.0%	0.80 [−2.06, 3.66]
Ibarra (medium) 2019	8.6	4	48	8.8	5	16	3.3%	−0.20 [−2.90, 2.50]
Ibarra (low) 2019	7.4	3.7	48	8.8	5	16	3.3%	−1.40 [−4.06, 1.26]
Schoemaker (high) 2022	4.3	1	44	3.7	1.6	22	8.6%	0.60 [−0.13, 1.33]
Schoemaker (low) 2022	3.8	1.6	45	3.7	1.6	21	8.3%	0.10 [−0.73, 0.93]
Waitzberg 2012	5.95	2.5	21	6.7	3.83	23	5.0%	−0.75 [−2.65, 1.15]
**Subtotal (95% CI)**			**355**			**208**	**42.0%**	**0.93 [−0.31, 2.18]**
Heterogeneity: Tau² = 2.27; Chi² = 37.29, df = 7 (P < 0.00001); I² = 81%								
Test for overall effect: Z = 1.47 (P = 0.14)								
**4.2.2 Duration of treatment ≥ 4 weeks**								
Ashraf 1995	3.8	1.33	11	3	1.33	11	7.4%	0.80 [−0.31, 1.91]
Badiali 1995	7.2	2.2	12	3.5	1.4	12	6.2%	3.70 [2.22, 5.18]
Duncan (high) 2018	−0.6	3.3	34	0.1	2.7	18	5.6%	−0.70 [−2.37, 0.97]
Duncan (low) 2018	0.8	2.6	34	0.1	2.7	18	6.0%	0.70 [−0.82, 2.22]
Huh 2007	5.18	0.56	22	4.13	1.33	20	8.9%	1.05 [0.42, 1.68]
Li 2017	3.02	1.15	52	1.86	0.58	51	9.6%	1.16 [0.81, 1.51]
Micka 2017	3.67	1.53	44	3.17	1.15	44	9.1%	0.50 [−0.07, 1.07]
Tomás-Ridocci 1992	8	2.2	10	3.4	1.8	10	5.3%	4.60 [2.84, 6.36]
**Subtotal (95% CI)**			**219**			**184**	**58.0%**	**1.33 [0.62, 2.03]**
Heterogeneity: Tau² = 0.70; Chi² = 36.55, df = 7 (P < 0.00001); I² = 81%								
Test for overall effect: Z = 3.70 (P = 0.0002)								
**Total (95% CI)**			**574**			**392**	**100.0%**	**1.19 [0.59, 1.78]**
Heterogeneity: Tau² = 0.94; Chi² = 74.83, df = 15 (P < 0.00001); I² = 80%								
Test for overall effect: Z = 3.90 (P < 0.0001)								
Test for subgroup differences: Chi² = 0.29, df = 1 (P = 0.59), I² = 0%								

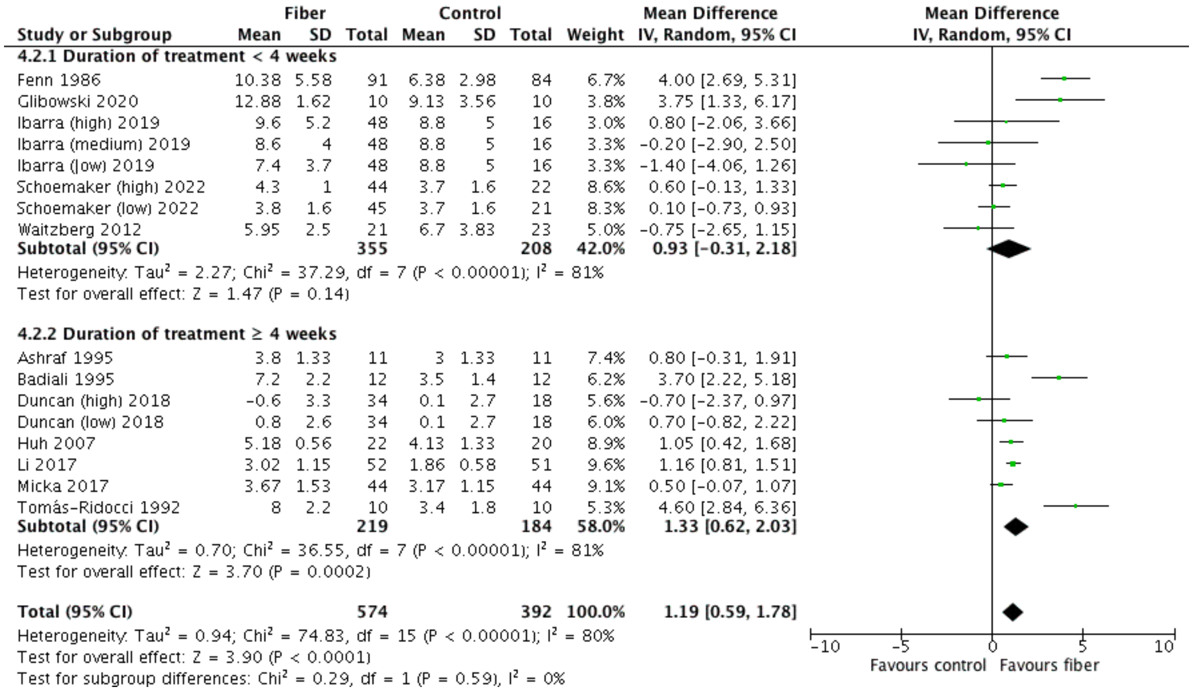

Supplementary Figure 3D Forest plot of subgroup analysis based on the treatment duration for stool frequency (bowel movements/week) in randomized controlled trials comparing fiber with control in adults with chronic constipation (n=966). Values were calculated as mean difference (95% CIs) using a random-effects model. Abbreviation: IV, inverse variance.

Stool consistency

Study or Subgroup	Std. Mean Difference	SE	Weight	Std. Mean Difference IV, Random, 95% CI	A	B	C	D	E	F
Ashraf 1995	0.6202	0.44	2.8%	0.62 [−0.24, 1.48]	?	?	−	?	?	−
Duncan (high) 2018	0.4231	0.29	6.4%	0.42 [−0.15, 0.99]	?	+	+	+	?	?
Duncan (low) 2018	0.1923	0.29	6.4%	0.19 [−0.38, 0.76]	?	+	+	+	?	?
Fenn 1986	0.4503	0.15	23.8%	0.45 [0.16, 0.74]	?	−	−	+	?	−
Ibarra (high) 2019	0.3803	0.29	6.4%	0.38 [−0.19, 0.95]	+	+	+	+	?	?
Ibarra (medium) 2019	−0.0099	0.29	6.4%	−0.01 [−0.58, 0.56]	+	+	+	+	?	?
Ibarra (low) 2019	−0.0287	0.29	6.4%	−0.03 [−0.60, 0.54]	+	+	+	+	?	?
Li 2017	1.5354	0.88	0.7%	1.54 [−0.19, 3.26]	?	+	+	?	?	?
López Román 2008	0.5993	0.36	4.1%	0.60 [−0.11, 1.30]	?	−	−	+	−	−
Marteau 2011	0.5687	0.29	6.4%	0.57 [0.00, 1.14]	?	−	−	?	−	−
Micka 2017	0.257	0.21	12.2%	0.26 [−0.15, 0.67]	+	−	?	+	?	−
Schoemaker (high) 2022	0.171	0.26	7.9%	0.17 [−0.34, 0.68]	?	+	+	+	+	?
Schoemaker (low) 2022	−0.0769	0.26	7.9%	−0.08 [−0.59, 0.43]	?	+	+	+	+	?
Tomás-Ridocci 1992	1.0659	0.48	2.3%	1.07 [0.13, 2.01]	?	+	−	−	?	−
**Total (95% CI)**			**100.0%**	**0.32 [0.18, 0.46]**						

Heterogeneity: Tau² = 0.00; Chi² = 12.73, df = 13 (P = 0.47); I² = 0%
Test for overall effect: Z = 4.38 (P < 0.0001)

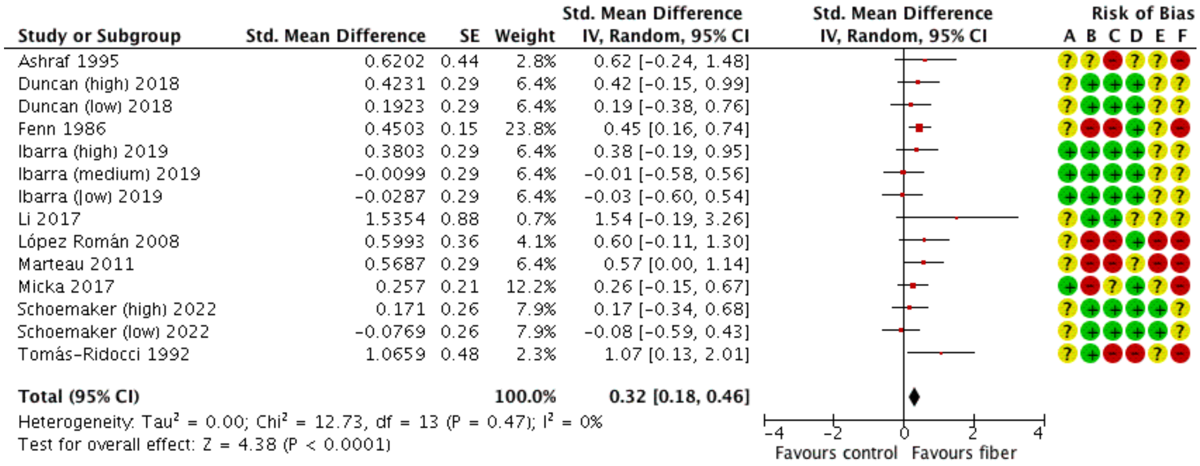

Supplementary Figure 4A Forest plot of stool consistency in randomized controlled trials comparing fiber with control in adults with chronic constipation (n=918). Values were calculated as standardized mean difference (95% CIs) using a random-effects model. Risk of bias key: A = bias arising from the randomization process; B=bias arising from deviations from intended interventions; C=bias due to missing outcome data; D=bias in measurement of the outcome; E=bias in selection of the reported result; F=overall risk of bias. Abbreviation: IV, inverse variance.

Study or Subgroup	Std. Mean Difference	SE	Weight	Std. Mean Difference IV, Random, 95% CI
**3.4.1 Prebiotics**				
Li 2017	1.5354	0.88	0.7%	1.54 [−0.19, 3.26]
Marteau 2011	0.5687	0.29	6.4%	0.57 [0.00, 1.14]
Micka 2017	0.257	0.21	12.2%	0.26 [−0.15, 0.67]
Schoemaker (high) 2022	0.171	0.26	7.9%	0.17 [−0.34, 0.68]
Schoemaker (low) 2022	−0.0769	0.26	7.9%	−0.08 [−0.59, 0.43]
**Subtotal (95% CI)**			**35.1%**	**0.25 [−0.03, 0.53]**
Heterogeneity: $Tau^2 = 0.02$; $Chi^2 = 5.01$, df = 4 (P = 0.29); $I^2 = 20\%$				
Test for overall effect: Z = 1.77 (P = 0.08)				
**3.4.2 Mixture of prebiotics with non-prebiotics**				
López Román 2008	0.5993	0.36	4.1%	0.60 [−0.11, 1.30]
**Subtotal (95% CI)**			**4.1%**	**0.60 [−0.11, 1.30]**
Heterogeneity: Not applicable				
Test for overall effect: Z = 1.66 (P = 0.10)				
**3.4.3 Non-prebiotics**				
Ashraf 1995	0.6202	0.44	2.8%	0.62 [−0.24, 1.48]
Duncan (high) 2018	0.4231	0.29	6.4%	0.42 [−0.15, 0.99]
Duncan (low) 2018	0.1923	0.29	6.4%	0.19 [−0.38, 0.76]
Fenn 1986	0.4503	0.15	23.8%	0.45 [0.16, 0.74]
Ibarra (high) 2019	0.3803	0.29	6.4%	0.38 [−0.19, 0.95]
Ibarra (medium) 2019	−0.0099	0.29	6.4%	−0.01 [−0.58, 0.56]
Ibarra (low) 2019	−0.0287	0.29	6.4%	−0.03 [−0.60, 0.54]
Tomás-Ridocci 1992	1.0659	0.48	2.3%	1.07 [0.13, 2.01]
**Subtotal (95% CI)**			**60.8%**	**0.35 [0.16, 0.53]**
Heterogeneity: $Tau^2 = 0.00$; $Chi^2 = 6.66$, df = 7 (P = 0.46); $I^2 = 0\%$				
Test for overall effect: Z = 3.68 (P = 0.0002)				
**Total (95% CI)**			**100.0%**	**0.32 [0.18, 0.46]**
Heterogeneity: $Tau^2 = 0.00$; $Chi^2 = 12.73$, df = 13 (P = 0.47); $I^2 = 0\%$				
Test for overall effect: Z = 4.38 (P < 0.0001)				
Test for subgroup differences: $Chi^2 = 0.87$, df = 2 (P = 0.65), $I^2 = 0\%$				

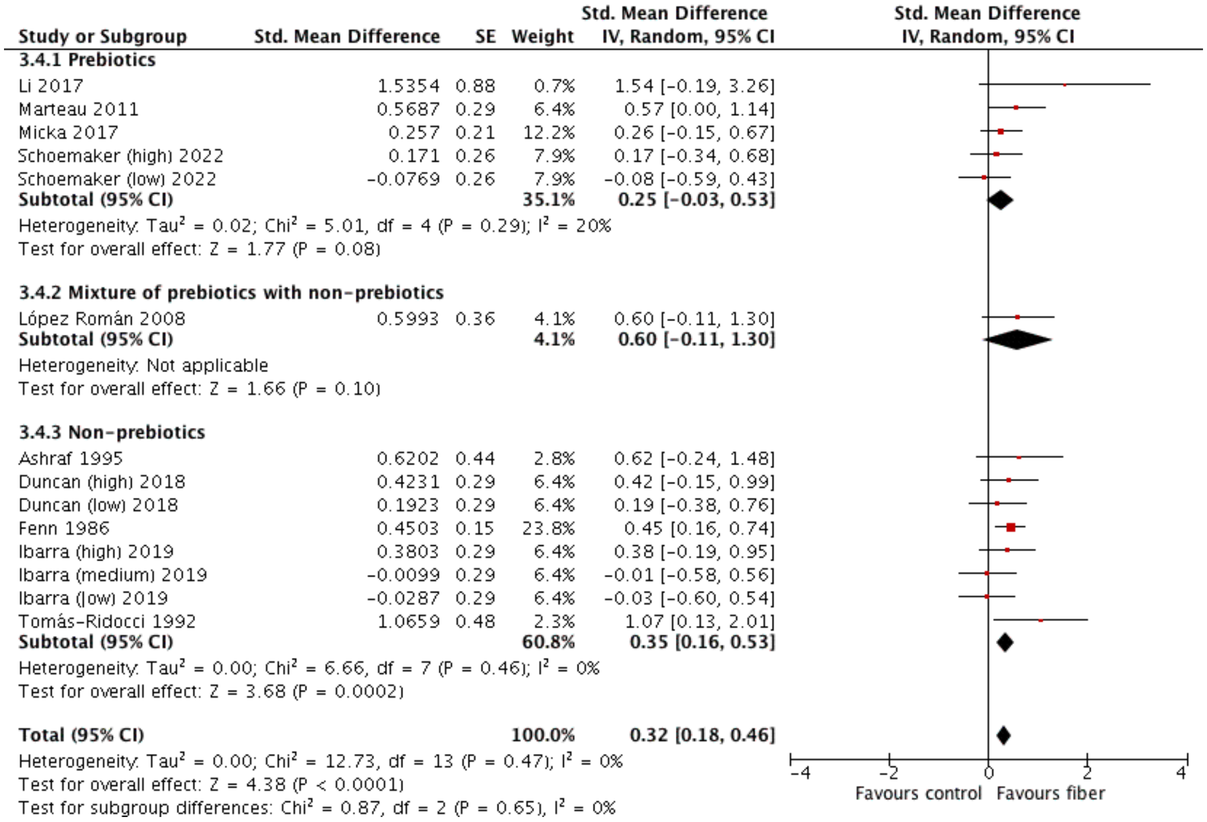

Supplementary Figure 4B Forest plot of subgroup analysis based on prebiotic status of the intervention for stool consistency in randomized controlled trials comparing fiber with control in adults with chronic constipation (n=918). Values were calculated as standardized mean difference (95% CIs) using a random-effects model. Abbreviation: IV, inverse variance.

Study or Subgroup	Std. Mean Difference	SE	Weight	Std. Mean Difference IV, Random, 95% CI
**5.4.1 Low dose (≤10 g/d)**				
Duncan (low) 2018	0.1923	0.29	6.4%	0.19 [−0.38, 0.76]
Ibarra (medium) 2019	−0.0099	0.29	6.4%	−0.01 [−0.58, 0.56]
Ibarra (low) 2019	−0.0287	0.29	6.4%	−0.03 [−0.60, 0.54]
Li 2017	1.5354	0.88	0.7%	1.54 [−0.19, 3.26]
Schoemaker (low) 2022	−0.0769	0.26	7.9%	−0.08 [−0.59, 0.43]
**Subtotal (95% CI)**			**27.7%**	**0.05 [−0.22, 0.32]**
Heterogeneity: Tau² = 0.00; Chi² = 3.44, df = 4 (P = 0.49); I² = 0%				
Test for overall effect: Z = 0.37 (P = 0.71)				
**5.4.2 High dose (>10 g/d)**				
Ashraf 1995	0.6202	0.44	2.8%	0.62 [−0.24, 1.48]
Duncan (high) 2018	0.4231	0.29	6.4%	0.42 [−0.15, 0.99]
Fenn 1986	0.4503	0.15	23.8%	0.45 [0.16, 0.74]
Ibarra (high) 2019	0.3803	0.29	6.4%	0.38 [−0.19, 0.95]
López Román 2008	0.5993	0.36	4.1%	0.60 [−0.11, 1.30]
Marteau 2011	0.5687	0.29	6.4%	0.57 [0.00, 1.14]
Micka 2017	0.257	0.21	12.2%	0.26 [−0.15, 0.67]
Schoemaker (high) 2022	0.171	0.26	7.9%	0.17 [−0.34, 0.68]
Tomás-Ridocci 1992	1.0659	0.48	2.3%	1.07 [0.13, 2.01]
**Subtotal (95% CI)**			**72.3%**	**0.42 [0.26, 0.59]**
Heterogeneity: Tau² = 0.00; Chi² = 4.11, df = 8 (P = 0.85); I² = 0%				
Test for overall effect: Z = 4.92 (P < 0.00001)				
**Total (95% CI)**			**100.0%**	**0.32 [0.18, 0.46]**
Heterogeneity: Tau² = 0.00; Chi² = 12.73, df = 13 (P = 0.47); I² = 0%				
Test for overall effect: Z = 4.38 (P < 0.0001)				
Test for subgroup differences: Chi² = 5.18, df = 1 (P = 0.02), I² = 80.7%				

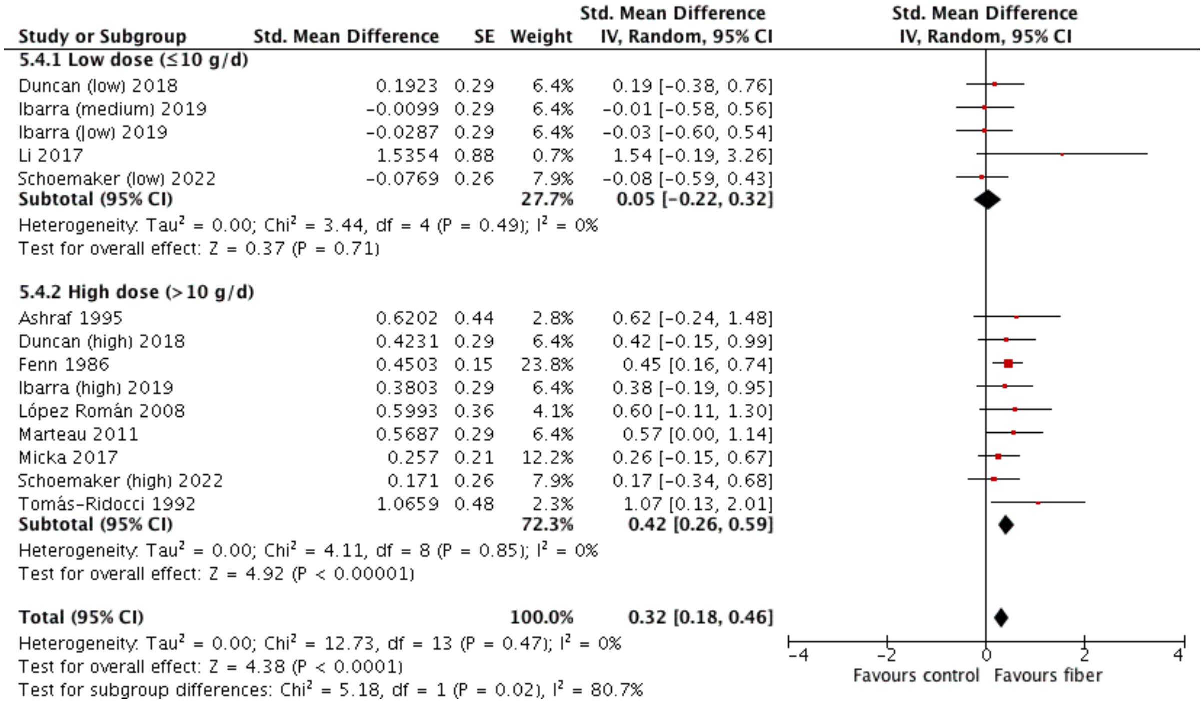

Supplementary Figure 4C Forest plot of subgroup analysis based on the dose administered for stool consistency in randomized controlled trials comparing fiber with control in adults with chronic constipation (n=918). Values were calculated as standardized mean difference (95% CIs) using a random-effects model. Abbreviation: IV, inverse variance.

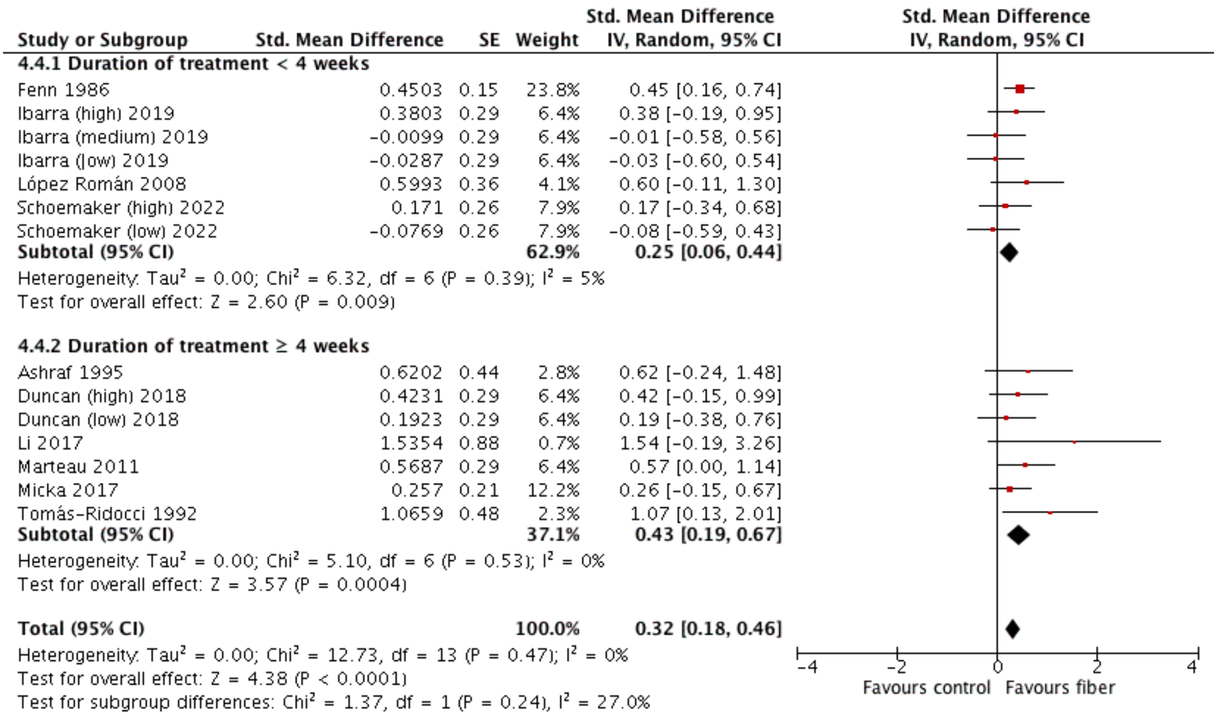

Supplementary Figure 4D Forest plot of subgroup analysis based on the treatment duration for stool consistency in randomized controlled trials comparing fiber with control in adults with chronic constipation (n=918). Values were calculated as standardized mean difference (95% CIs) using a random-effects model. Abbreviation: IV, inverse variance.

<u>Stool weight</u>

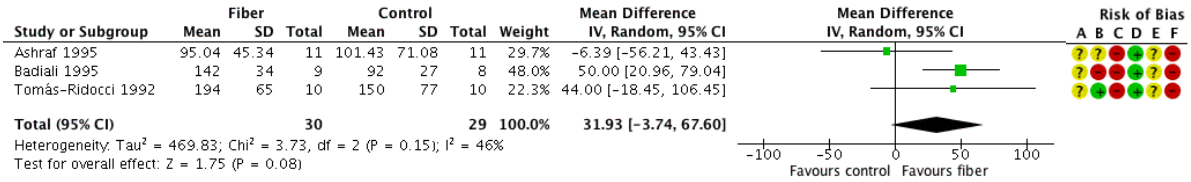

Supplementary Figure 5A Forest plot of stool weight in randomized controlled trials comparing fiber with control in adults with chronic constipation (n=59). Values were calculated as mean difference (95% CIs) using a random-effects model. Risk of bias key. A=bias arising from the randomization process; B=bias arising from deviations from intended interventions; C=bias due to missing outcome data; D=bias in measurement of the outcome; E=bias in selection of the reported result F=overall risk of bias. Abbreviation: IV, inverse variance.

Study or Subgroup	Fiber Mean	Fiber SD	Fiber Total	Control Mean	Control SD	Control Total	Weight	Mean Difference IV, Random, 95% CI
**2.7.1 Psyllium**								
Ashraf 1995	95.04	45.34	11	101.43	71.08	11	29.7%	-6.39 [-56.21, 43.43]
Tomás-Ridocci 1992	194	65	10	150	77	10	22.3%	44.00 [-18.45, 106.45]
**Subtotal (95% CI)**			**21**			**21**	**52.0%**	**15.14 [-33.71, 64.00]**
Heterogeneity: $Tau^2 = 438.78$; $Chi^2 = 1.53$, df = 1 (P = 0.22); $I^2 = 35\%$								
Test for overall effect: Z = 0.61 (P = 0.54)								
**2.7.2 Wheat bran**								
Badiali 1995	142	34	9	92	27	8	48.0%	50.00 [20.96, 79.04]
**Subtotal (95% CI)**			**9**			**8**	**48.0%**	**50.00 [20.96, 79.04]**
Heterogeneity: Not applicable								
Test for overall effect: Z = 3.37 (P = 0.0007)								
**Total (95% CI)**			**30**			**29**	**100.0%**	**31.93 [-3.74, 67.60]**
Heterogeneity: $Tau^2 = 469.83$; $Chi^2 = 3.73$, df = 2 (P = 0.15); $I^2 = 46\%$								
Test for overall effect: Z = 1.75 (P = 0.08)								
Test for subgroup differences: $Chi^2 = 1.44$, df = 1 (P = 0.23), $I^2 = 30.8\%$								

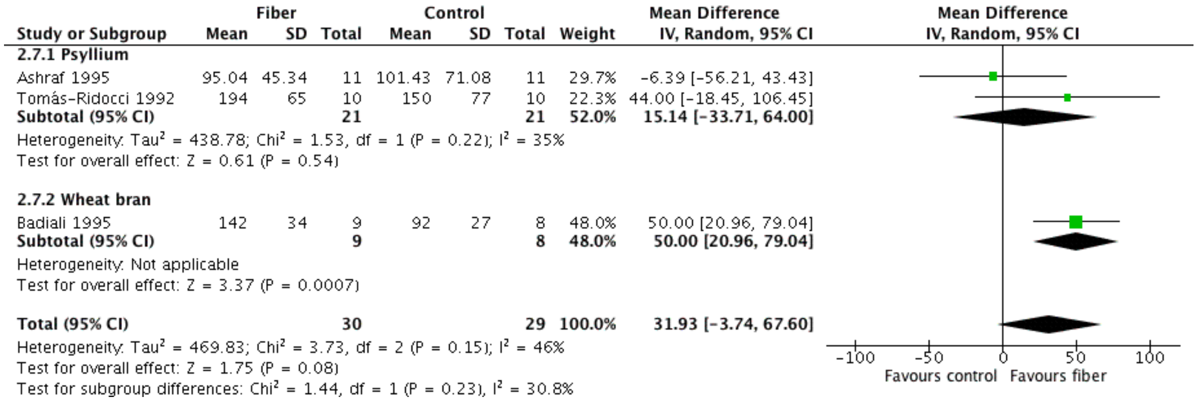

Supplementary Figure 5B Forest plot of subgroup analysis based on type of fiber for stool weight in randomized controlled trials comparing fiber with control in adults with chronic constipation (n=59). Values were calculated as mean difference (95% CIs) using a random-effects model. Abbreviation: IV, inverse variance.

Whole gut transit time

Study or Subgroup	Fiber Mean	Fiber SD	Fiber Total	Control Mean	Control SD	Control Total	Weight	Mean Difference IV, Random, 95% CI	Risk of Bias A	B	C	D	E	F
Ashraf 1995	54.2	16.58	11	54	19.9	11	10.6%	0.20 [−15.11, 15.51]	?	?	-	+	?	-
Badiali 1995	88	42	12	168	48	12	5.3%	−80.00 [−116.09, −43.91]	?	-	-	+	?	-
Duncan (high) 2018	6.8	26.1	35	−9.4	27	17	10.5%	16.20 [0.72, 31.68]	?	+	+	+	?	?
Duncan (low) 2018	−0.9	32.8	35	−9.4	27	17	10.1%	8.50 [−8.32, 25.32]	?	+	+	+	?	?
Huh 2007	25.2	20.7	22	41.9	25.2	20	11.0%	−16.70 [−30.73, −2.67]	?	-	-	+	?	-
Ibarra (high) 2019	38	28	48	45	31	16	10.0%	−7.00 [−24.13, 10.13]	+	+	+	+	?	?
Ibarra (medium) 2019	44	35	48	45	31	16	9.7%	−1.00 [−19.13, 17.13]	+	+	+	+	?	?
Ibarra (low) 2019	50	43	48	45	31	16	9.3%	5.00 [−14.46, 24.46]	+	+	+	+	?	?
Tomás-Ridocci 1992	34	18	10	45	18	10	10.4%	−11.00 [−26.78, 4.78]	?	+	-	+	?	-
Xu 2014	60.2	11.2	40	79.4	11.7	40	13.2%	−19.20 [−24.22, −14.18]	?	+	+	+	?	?
**Total (95% CI)**			**309**			**175**	**100.0%**	**−7.46 [−18.05, 3.12]**						

Heterogeneity: $Tau^2 = 215.13$; $Chi^2 = 47.55$, $df = 9$ ($P < 0.00001$); $I^2 = 81\%$
Test for overall effect: $Z = 1.38$ ($P = 0.17$)

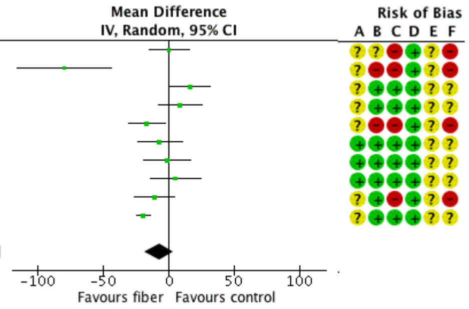

Supplementary Figure 6A Forest plot of whole gut transit time in randomized controlled trials comparing fiber with control in adults with chronic constipation (n=485). Values were calculated as mean difference (95% CIs) using a random-effects model. Risk of bias key: A=bias arising from the randomization process; B=bias arising from deviations from intended interventions; C=bias due to missing outcome data; D=bias in measurement of the outcome; E=bias in selection of the reported result; F=overall risk of bias. Abbreviation: IV, inverse variance.

Study or Subgroup	Fiber Mean	Fiber SD	Fiber Total	Control Mean	Control SD	Control Total	Weight	Mean Difference IV, Random, 95% CI
**2.5.1 Polydextrose**								
Duncan (high) 2018	6.8	26.1	35	-9.4	27	17	10.5%	16.20 [0.72, 31.68]
Duncan (low) 2018	-0.9	32.8	35	-9.4	27	17	10.1%	8.50 [-8.32, 25.32]
Ibarra (high) 2019	38	28	48	45	31	16	10.0%	-7.00 [-24.13, 10.13]
Ibarra (medium) 2019	44	35	48	45	31	16	9.7%	-1.00 [-19.13, 17.13]
Ibarra (low) 2019	50	43	48	45	31	16	9.3%	5.00 [-14.46, 24.46]
**Subtotal (95% CI)**			**214**			**82**	**49.6%**	**4.92 [-3.26, 13.10]**
Heterogeneity: $Tau^2 = 9.43$; $Chi^2 = 4.48$, df = 4 (P = 0.34); $I^2 = 11\%$								
Test for overall effect: Z = 1.18 (P = 0.24)								
**2.5.2 Psyllium**								
Ashraf 1995	54.2	16.58	11	54	19.9	11	10.6%	0.20 [-15.11, 15.51]
Tomás-Ridocci 1992	34	18	10	45	18	10	10.4%	-11.00 [-26.78, 4.78]
**Subtotal (95% CI)**			**21**			**21**	**21.0%**	**-5.23 [-16.22, 5.76]**
Heterogeneity: $Tau^2 = 0.00$; $Chi^2 = 1.00$, df = 1 (P = 0.32); $I^2 = 0\%$								
Test for overall effect: Z = 0.93 (P = 0.35)								
**2.5.3 Wheat bran**								
Badiali 1995	88	42	12	168	48	12	5.3%	-80.00 [-116.09, -43.91]
**Subtotal (95% CI)**			**12**			**12**	**5.3%**	**-80.00 [-116.09, -43.91]**
Heterogeneity: Not applicable								
Test for overall effect: Z = 4.35 (P < 0.0001)								
**2.5.6 Pectin**								
Xu 2014	60.2	11.2	40	79.4	11.7	40	13.2%	-19.20 [-24.22, -14.18]
**Subtotal (95% CI)**			**40**			**40**	**13.2%**	**-19.20 [-24.22, -14.18]**
Heterogeneity: Not applicable								
Test for overall effect: Z = 7.50 (P < 0.00001)								
**2.5.7 Unspecified**								
Huh 2007	25.2	20.7	22	41.9	25.2	20	11.0%	-16.70 [-30.73, -2.67]
**Subtotal (95% CI)**			**22**			**20**	**11.0%**	**-16.70 [-30.73, -2.67]**
Heterogeneity: Not applicable								
Test for overall effect: Z = 2.33 (P = 0.02)								
**Total (95% CI)**			**309**			**175**	**100.0%**	**-7.46 [-18.05, 3.12]**
Heterogeneity: $Tau^2 = 215.13$; $Chi^2 = 47.55$, df = 9 (P < 0.00001); $I^2 = 81\%$								
Test for overall effect: Z = 1.38 (P = 0.17)								
Test for subgroup differences: $Chi^2 = 39.71$, df = 4 (P < 0.00001), $I^2 = 89.9\%$								

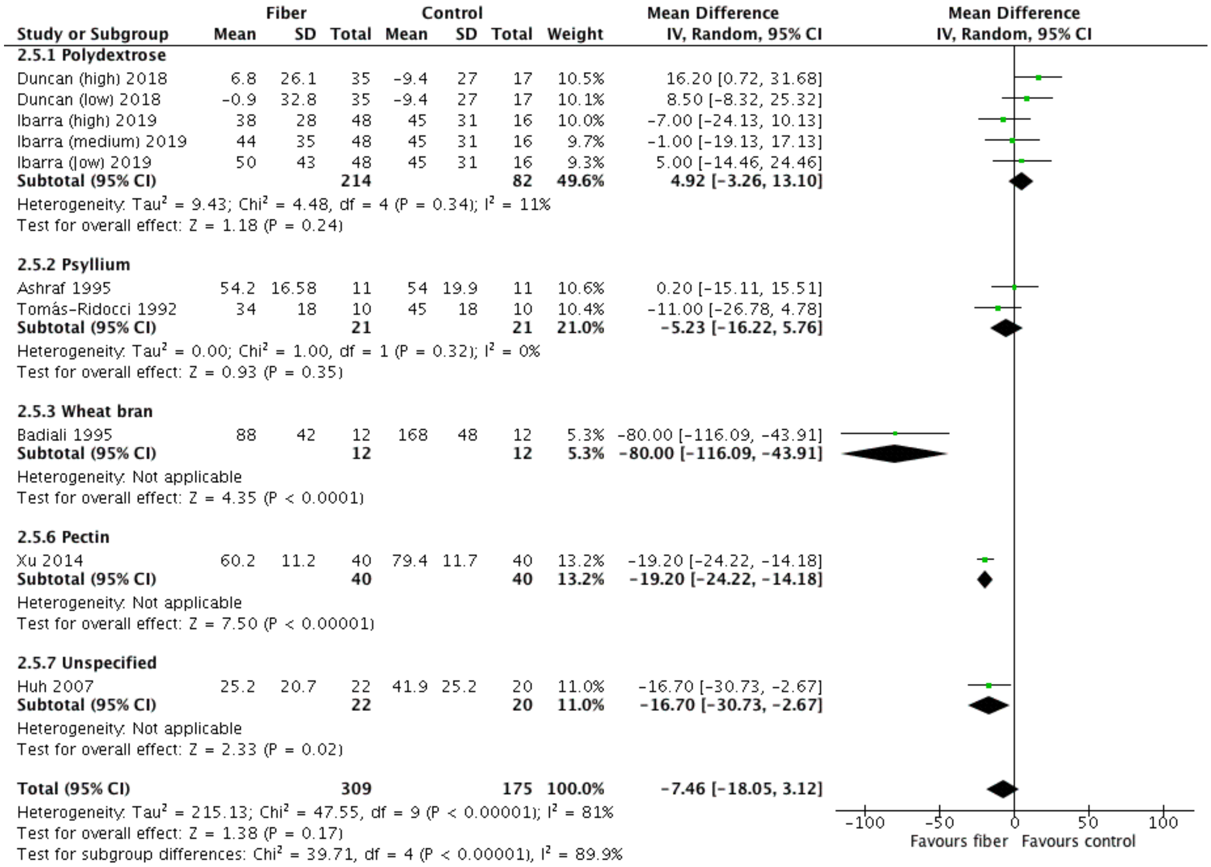

Supplementary Figure 6B Forest plot of subgroup analysis based on the type of fiber for whole gut transit time in randomized controlled trials comparing fiber with control in adults with chronic constipation (n=485). Values were calculated as mean difference (95% CIs) using a random-effects model. Abbreviation: IV, inverse variance.

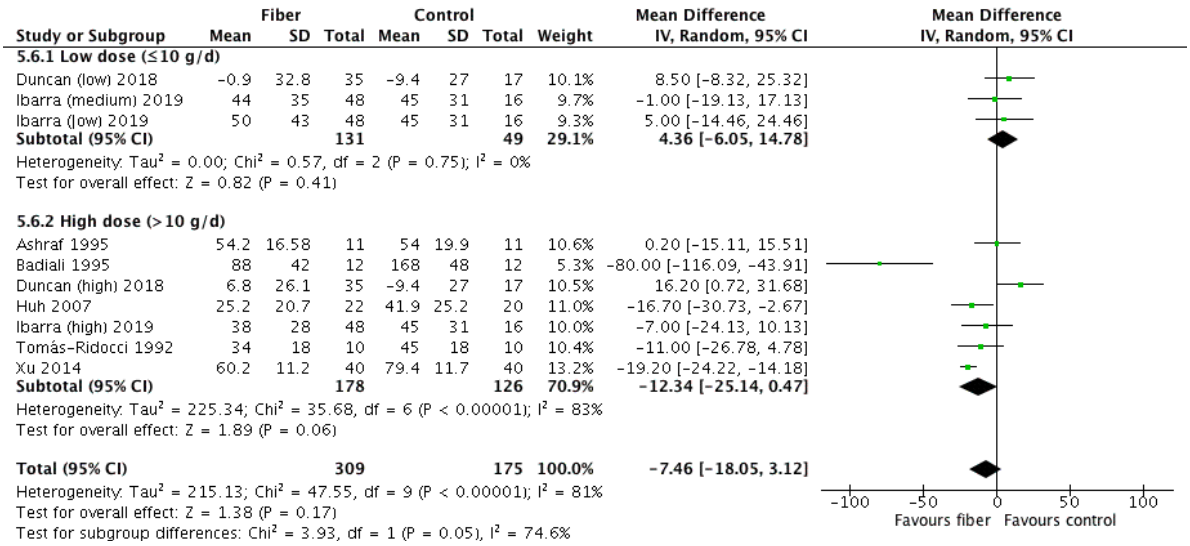

Supplementary Figure 6C Forest plot of subgroup analysis based on the dose administered for whole gut transit time in randomized controlled trials comparing fiber with control in adults with chronic constipation (n=485). Values were calculated as mean difference (95% CIs) using a random-effects model. Abbreviation: IV, inverse variance.

Study or Subgroup	Fiber Mean	Fiber SD	Fiber Total	Control Mean	Control SD	Control Total	Weight	Mean Difference IV, Random, 95% CI
**4.5.1 Duration of treatment < 4 weeks**								
Duncan (high) 2018	6.8	26.1	35	-9.4	27	17	10.5%	16.20 [0.72, 31.68]
Duncan (low) 2018	-0.9	32.8	35	-9.4	27	17	10.1%	8.50 [-8.32, 25.32]
Ibarra (high) 2019	38	28	48	45	31	16	10.0%	-7.00 [-24.13, 10.13]
Ibarra (medium) 2019	44	35	48	45	31	16	9.7%	-1.00 [-19.13, 17.13]
Ibarra (low) 2019	50	43	48	45	31	16	9.3%	5.00 [-14.46, 24.46]
**Subtotal (95% CI)**			**214**			**82**	**49.6%**	**4.92 [-3.26, 13.10]**
Heterogeneity: Tau² = 9.43; Chi² = 4.48, df = 4 (P = 0.34); I² = 11%								
Test for overall effect: Z = 1.18 (P = 0.24)								
**4.5.2 Duration of treatment ≥ 4 weeks**								
Ashraf 1995	54.2	16.58	11	54	19.9	11	10.6%	0.20 [-15.11, 15.51]
Badiali 1995	88	42	12	168	48	12	5.3%	-80.00 [-116.09, -43.91]
Huh 2007	25.2	20.7	22	41.9	25.2	20	11.0%	-16.70 [-30.73, -2.67]
Tomás-Ridocci 1992	34	18	10	45	18	10	10.4%	-11.00 [-26.78, 4.78]
Xu 2014	60.2	11.2	40	79.4	11.7	40	13.2%	-19.20 [-24.22, -14.18]
**Subtotal (95% CI)**			**95**			**93**	**50.4%**	**-18.44 [-31.52, -5.37]**
Heterogeneity: Tau² = 152.86; Chi² = 17.76, df = 4 (P = 0.001); I² = 77%								
Test for overall effect: Z = 2.76 (P = 0.006)								
**Total (95% CI)**			**309**			**175**	**100.0%**	**-7.46 [-18.05, 3.12]**
Heterogeneity: Tau² = 215.13; Chi² = 47.55, df = 9 (P < 0.00001); I² = 81%								
Test for overall effect: Z = 1.38 (P = 0.17)								
Test for subgroup differences: Chi² = 8.81, df = 1 (P = 0.003), I² = 88.7%								

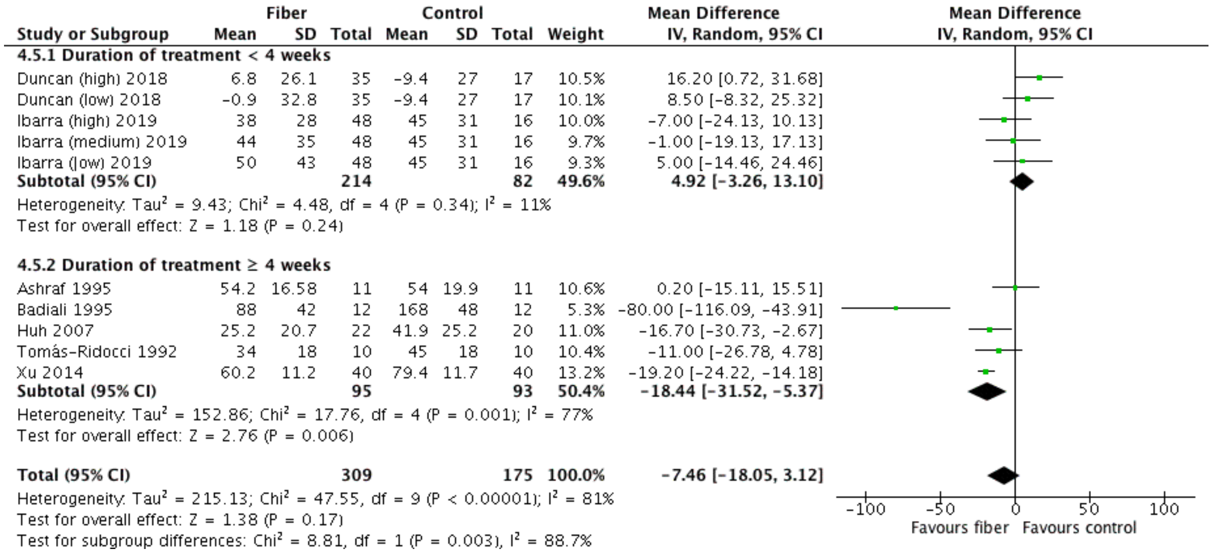

Supplementary Figure 6D Forest plot of subgroup analysis based on the treatment duration for whole gut transit time in randomized controlled trials comparing fiber with control in adults with chronic constipation (n=485). Values were calculated as mean difference (95% CIs) using a random-effects model. Abbreviation: IV, inverse variance.

Regional gut transit time

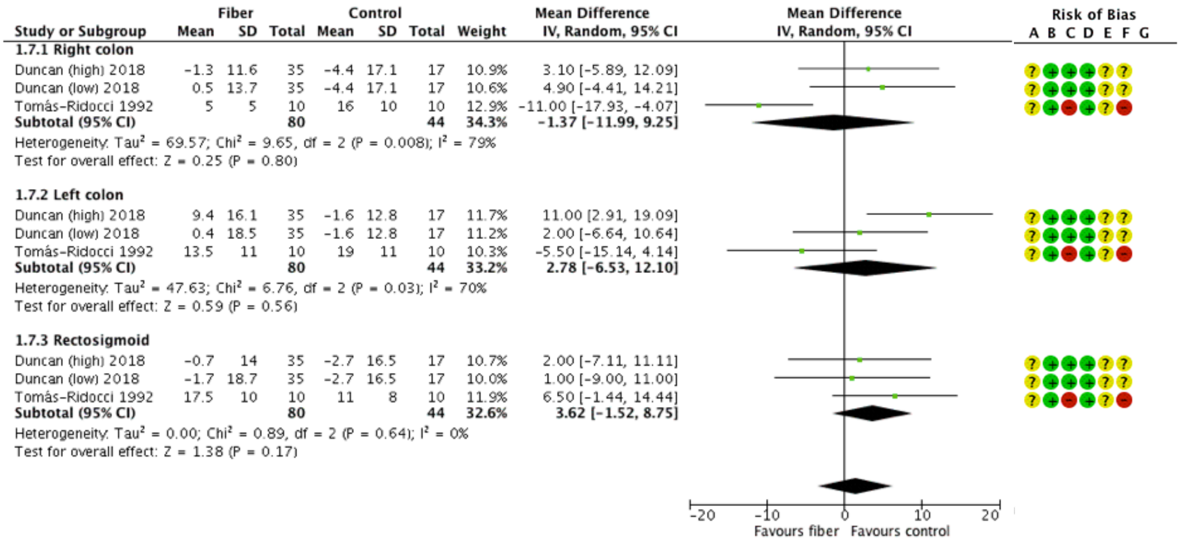

Supplementary Figure 7 Forest plot of right, left and rectosigmoid transit time in randomized controlled trials comparing fiber with control in adults with chronic constipation (n=125). Values were calculated as mean difference (95% CIs) using a random-effects model. Risk of bias key: A=bias arising from the randomization process; B=bias arising from deviations from intended interventions; C=bias due to missing outcome data; D=bias in measurement of the outcome; E=bias in selection of the reported result; F=overall risk of bias. Abbreviation: IV, inverse variance.

Integrative symptom score

Study or Subgroup	Std. Mean Difference	SE	Weight	Std. Mean Difference IV, Random, 95% CI	Std. Mean Difference IV, Random, 95% CI	Risk of Bias A	B	C	D	E	F
Badiali 1995	0.1889	0.42	6.4%	0.19 [-0.63, 1.01]		?	-	-	+	?	-
Duncan (high) 2018	-0.4452	0.29	10.9%	-0.45 [-1.01, 0.12]		?	+	+	+	?	?
Duncan (low) 2018	-0.2804	0.29	10.9%	-0.28 [-0.85, 0.29]		?	+	+	+	?	?
Ibarra (high) 2019	0.0518	0.29	10.9%	0.05 [-0.52, 0.62]		+	+	+	+	?	?
Ibarra (medium) 2019	-0.0615	0.29	10.9%	-0.06 [-0.63, 0.51]		+	+	+	+	?	?
Ibarra (low) 2019	0.3057	0.29	10.9%	0.31 [-0.26, 0.87]		+	+	+	+	?	?
Schoemaker (high) 2022	-0.1807	0.26	12.5%	-0.18 [-0.69, 0.33]		?	+	+	+	?	?
Schoemaker (low) 2022	0.091	0.26	12.5%	0.09 [-0.42, 0.60]		?	+	+	+	?	?
Xu 2014	-0.7557	0.23	14.3%	-0.76 [-1.21, -0.30]		?	+	+	+	?	?
**Total (95% CI)**			**100.0%**	**-0.15 [-0.39, 0.08]**							

Heterogeneity: $Tau^2 = 0.05$; $Chi^2 = 12.68$, df = 8 (P = 0.12); $I^2 = 37\%$
Test for overall effect: Z = 1.29 (P = 0.20)

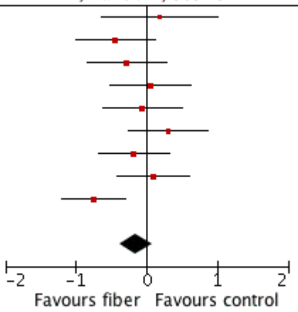

Supplementary Figure 8A Forest plot of integrative symptom score in randomized controlled trials comparing fiber with control in adults with chronic constipation (n=531). Values were calculated as standardized mean difference (95% CIs) using a random-effects model. Risk of bias key: A=bias arising from the randomization process; B=bias arising from deviations from intended interventions; C=bias due to missing outcome data; D=bias in measurement of the outcome; E=bias in selection of the reported result; F=overall risk of bias. Abbreviation: IV, inverse variance.

Study or Subgroup	Std. Mean Difference	SE	Weight	Std. Mean Difference IV, Random, 95% CI
**2.11.1 Polydextrose**				
Duncan (high) 2018	−0.4452	0.29	10.9%	−0.45 [−1.01, 0.12]
Duncan (low) 2018	−0.2804	0.29	10.9%	−0.28 [−0.85, 0.29]
Ibarra (high) 2019	0.0518	0.29	10.9%	0.05 [−0.52, 0.62]
Ibarra (medium) 2019	−0.0615	0.29	10.9%	−0.06 [−0.63, 0.51]
Ibarra (low) 2019	0.3057	0.29	10.9%	0.31 [−0.26, 0.87]
**Subtotal (95% CI)**			**54.4%**	**−0.09 [−0.34, 0.17]**
Heterogeneity: $Tau^2 = 0.00$; $Chi^2 = 4.04$, df = 4 (P = 0.40); $I^2 = 1\%$				
Test for overall effect: Z = 0.66 (P = 0.51)				
**2.11.2 Wheat bran**				
Badiali 1995	0.1889	0.42	6.4%	0.19 [−0.63, 1.01]
**Subtotal (95% CI)**			**6.4%**	**0.19 [−0.63, 1.01]**
Heterogeneity: Not applicable				
Test for overall effect: Z = 0.45 (P = 0.65)				
**2.11.3 Pectin**				
Xu 2014	−0.7557	0.23	14.3%	−0.76 [−1.21, −0.30]
**Subtotal (95% CI)**			**14.3%**	**−0.76 [−1.21, −0.30]**
Heterogeneity: Not applicable				
Test for overall effect: Z = 3.29 (P = 0.001)				
**2.11.4 Galacto-oligosaccharides**				
Schoemaker (high) 2022	−0.1807	0.26	12.5%	−0.18 [−0.69, 0.33]
Schoemaker (low) 2022	0.091	0.26	12.5%	0.09 [−0.42, 0.60]
**Subtotal (95% CI)**			**24.9%**	**−0.04 [−0.41, 0.32]**
Heterogeneity: $Tau^2 = 0.00$; $Chi^2 = 0.55$, df = 1 (P = 0.46); $I^2 = 0\%$				
Test for overall effect: Z = 0.24 (P = 0.81)				
**Total (95% CI)**			**100.0%**	**−0.15 [−0.39, 0.08]**
Heterogeneity: $Tau^2 = 0.05$; $Chi^2 = 12.68$, df = 8 (P = 0.12); $I^2 = 37\%$				
Test for overall effect: Z = 1.29 (P = 0.20)				
Test for subgroup differences: $Chi^2 = 8.09$, df = 3 (P = 0.04), $I^2 = 62.9\%$				

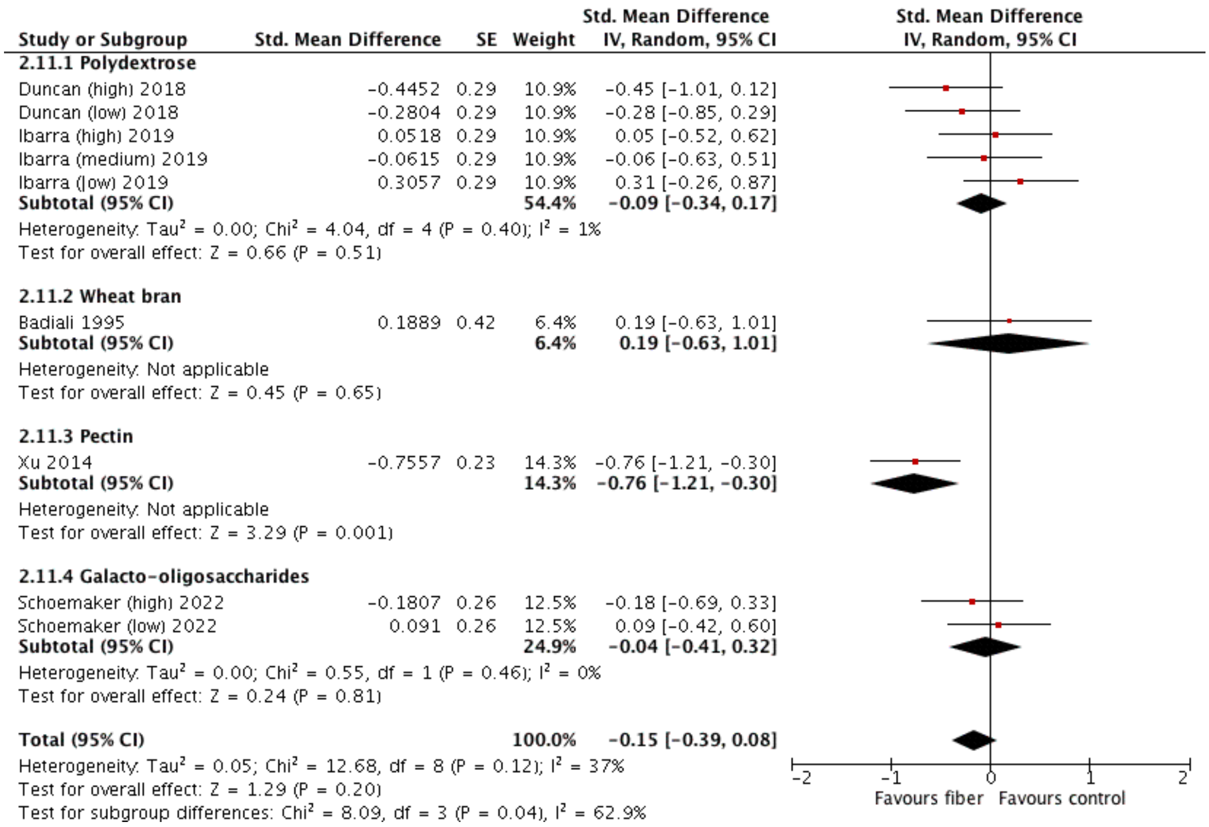

Supplementary Figure 8B Forest plot of subgroup analysis based on the type of fiber for integrative symptom score in randomized controlled trials comparing fiber with control in adults with chronic constipation (n=531). Values were calculated as standardized mean difference (95% CIs) using a random-effects model. Abbreviation: IV, inverse variance.

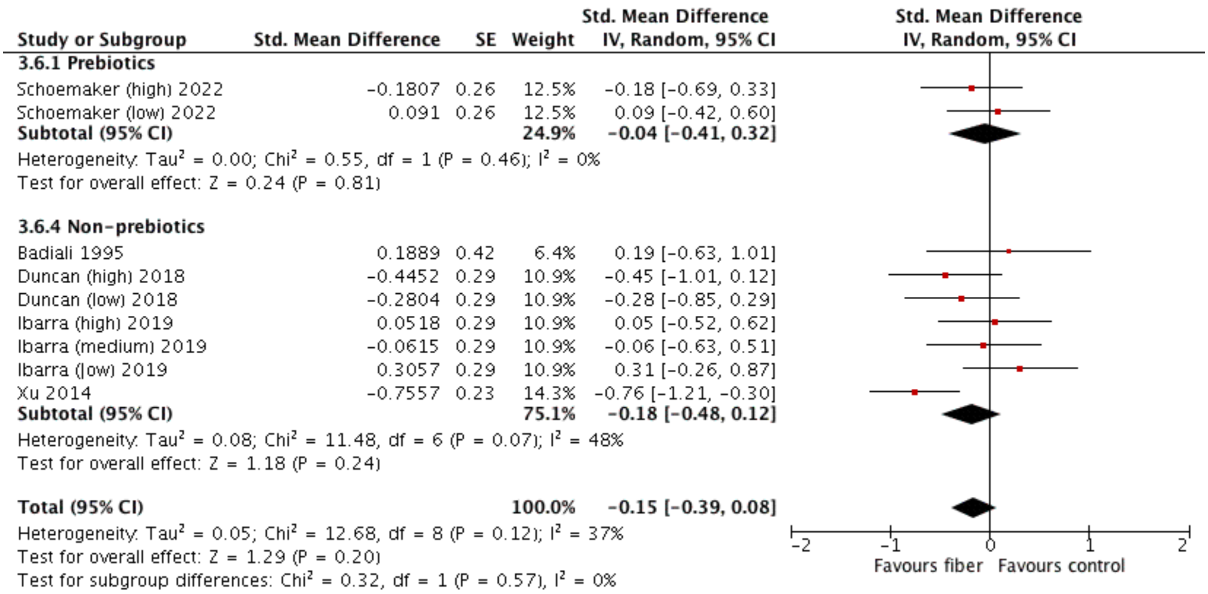

Supplementary Figure 8C Forest plot of subgroup analysis based on prebiotic status of the intervention for integrative symptom score in randomized controlled trials comparing fiber with control in adults with chronic constipation (n=531). Values were calculated as standardized mean difference (95% CIs) using a random-effects model. Abbreviation: IV, inverse variance.

Study or Subgroup	Std. Mean Difference	SE	Weight	Std. Mean Difference IV, Random, 95% CI
**5.11.1 Low dose (≤10 g/d)**				
Duncan (low) 2018	−0.2804	0.29	10.9%	−0.28 [−0.85, 0.29]
Ibarra (medium) 2019	−0.0615	0.29	10.9%	−0.06 [−0.63, 0.51]
Ibarra (low) 2019	0.3057	0.29	10.9%	0.31 [−0.26, 0.87]
Schoemaker (low) 2022	0.091	0.26	12.5%	0.09 [−0.42, 0.60]
**Subtotal (95% CI)**			**45.1%**	**0.02 [−0.26, 0.29]**
Heterogeneity: Tau² = 0.00; Chi² = 2.20, df = 3 (P = 0.53); I² = 0%				
Test for overall effect: Z = 0.13 (P = 0.90)				
**5.11.2 High dose (>10 g/d)**				
Badiali 1995	0.1889	0.42	6.4%	0.19 [−0.63, 1.01]
Duncan (high) 2018	−0.4452	0.29	10.9%	−0.45 [−1.01, 0.12]
Ibarra (high) 2019	0.0518	0.29	10.9%	0.05 [−0.52, 0.62]
Schoemaker (high) 2022	−0.1807	0.26	12.5%	−0.18 [−0.69, 0.33]
Xu 2014	−0.7557	0.23	14.3%	−0.76 [−1.21, −0.30]
**Subtotal (95% CI)**			**54.9%**	**−0.29 [−0.62, 0.05]**
Heterogeneity: Tau² = 0.06; Chi² = 7.17, df = 4 (P = 0.13); I² = 44%				
Test for overall effect: Z = 1.66 (P = 0.10)				
**Total (95% CI)**			**100.0%**	**−0.15 [−0.39, 0.08]**
Heterogeneity: Tau² = 0.05; Chi² = 12.68, df = 8 (P = 0.12); I² = 37%				
Test for overall effect: Z = 1.29 (P = 0.20)				
Test for subgroup differences: Chi² = 1.88, df = 1 (P = 0.17), I² = 46.8%				

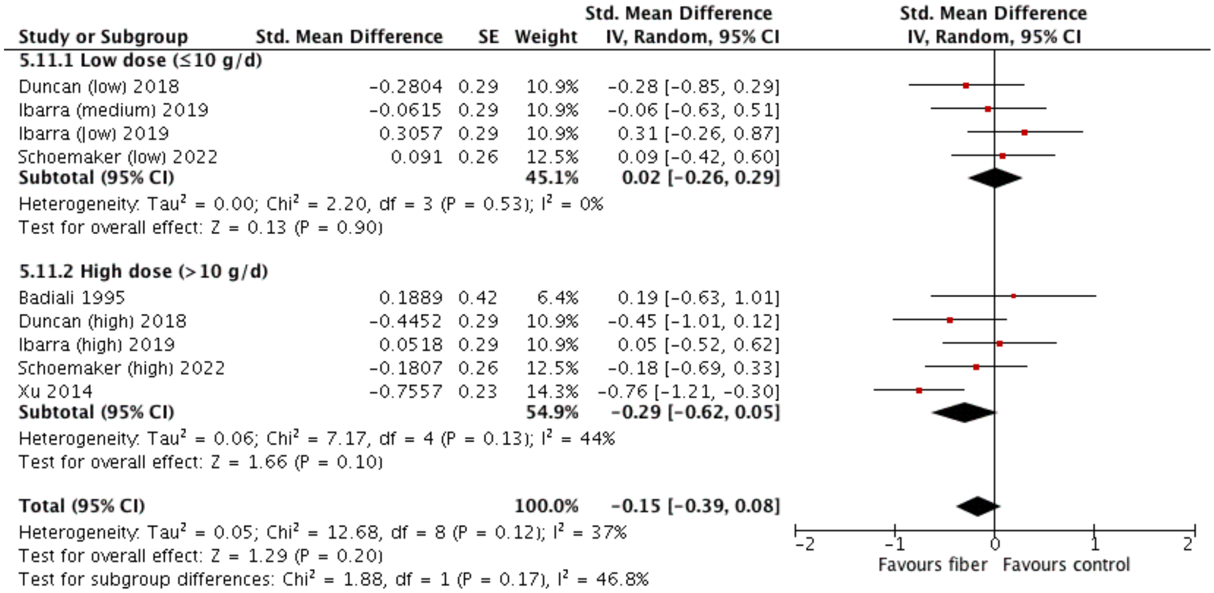

Supplementary Figure 8D Forest plot of subgroup analysis based on the dose administered for integrative symptom score in randomized controlled trials comparing fiber with control in adults with chronic constipation (n=531). Values were calculated as standardized mean difference (95% CIs) using a random-effects model. Abbreviation: IV, inverse variance.

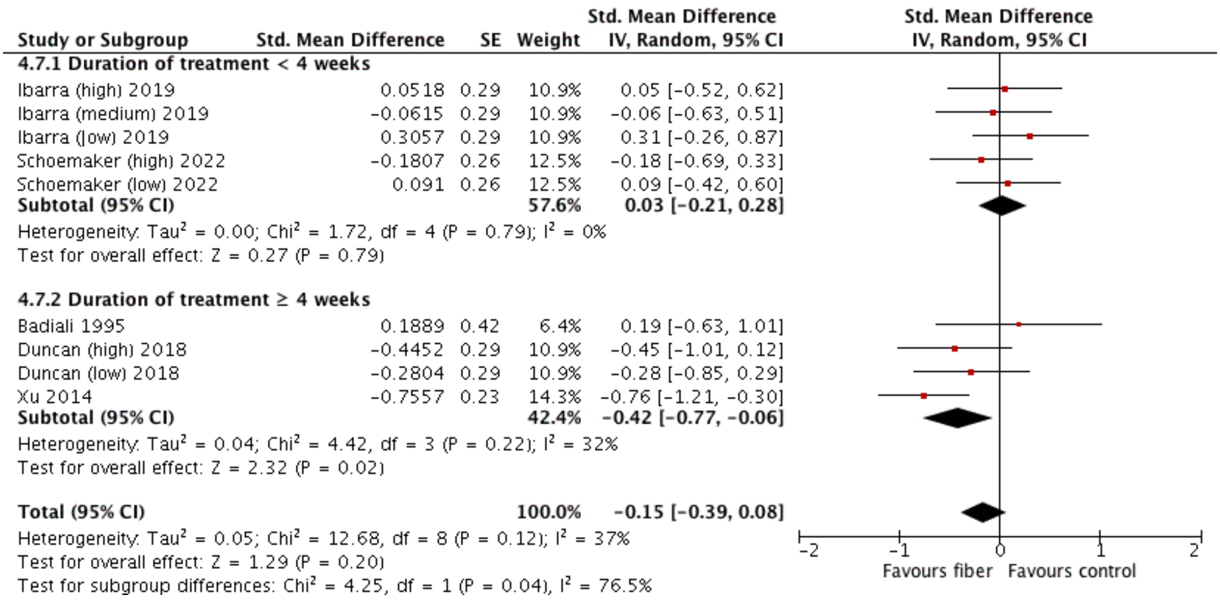

Supplementary Figure 8E Forest plot of subgroup analysis based on the treatment duration for integrative symptom score in randomized controlled trials comparing fiber with control in adults with chronic constipation (n=531). Values were calculated as standardized mean difference (95% CIs) using a random-effects model. Abbreviation: IV, inverse variance.

Patient Assessment of Constipation Symptoms (PAC-SYM) global and subscales (abdominal, rectal, stool)

Study or Subgroup	Fiber Mean	Fiber SD	Fiber Total	Control Mean	Control SD	Control Total	Weight	Mean Difference IV, Random, 95% CI
Duncan (high) 2018	−0.4	0.8	34	−0.2	0.5	18	17.5%	−0.20 [−0.55, 0.15]
Duncan (low) 2018	−0.7	0.6	34	−0.2	0.5	18	20.0%	−0.50 [−0.81, −0.19]
Ibarra (high) 2019	0.89	0.6	48	0.86	0.51	16	20.3%	0.03 [−0.27, 0.33]
Ibarra (medium) 2019	0.83	0.48	48	0.86	0.51	16	21.2%	−0.03 [−0.31, 0.25]
Ibarra (low) 2019	1.03	0.57	48	0.86	0.51	16	20.5%	0.17 [−0.13, 0.47]
Schoemaker (high) 2022	14.7	8.3	44	16	8.3	22	0.2%	−1.30 [−5.55, 2.95]
Schoemaker (low) 2022	17	9	45	16	8.3	21	0.2%	1.00 [−3.42, 5.42]
**Total (95% CI)**			**301**			**127**	**100.0%**	**−0.10 [−0.31, 0.11]**

Heterogeneity: $Tau^2 = 0.03$; $Chi^2 = 11.49$, df = 6 (P = 0.07); $I^2 = 48\%$
Test for overall effect: Z = 0.94 (P = 0.35)

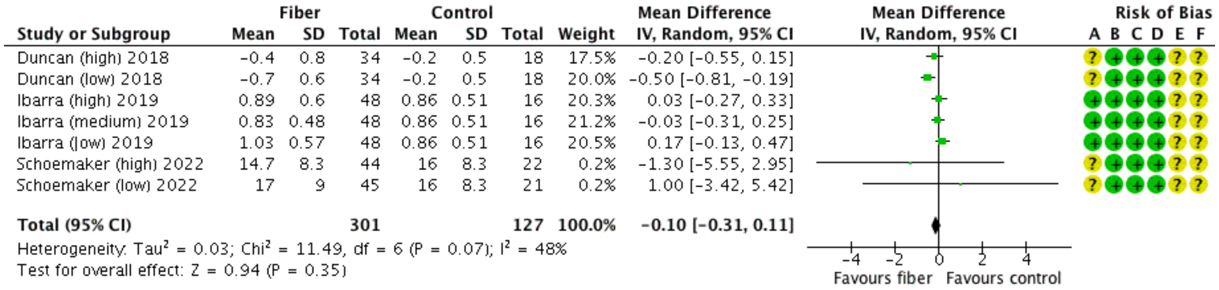

Supplementary Figure 9A Forest plot of PAC-SYM (global) in randomized controlled trials comparing fiber with control in adults with chronic constipation (n=428). Values were calculated as mean difference (95% CIs) using a random-effects model. Risk of bias key: A=bias arising from the randomization process; B=bias arising from deviations from intended interventions; C=bias due to missing outcome data; D=bias in measurement of the outcome; E=bias in selection of the reported result; F=overall risk of bias. Abbreviations: IV, inverse variance; PAC-SYM, Patient Assessment of Constipation Symptoms.

Study or Subgroup	Fiber Mean	Fiber SD	Fiber Total	Control Mean	Control SD	Control Total	Weight	Mean Difference IV, Random, 95% CI
**2.14.1 Polydextrose**								
Duncan (high) 2018	−0.4	0.8	34	−0.2	0.5	18	17.5%	−0.20 [−0.55, 0.15]
Duncan (low) 2018	−0.7	0.6	34	−0.2	0.5	18	20.0%	−0.50 [−0.81, −0.19]
Ibarra (high) 2019	0.89	0.6	48	0.86	0.51	16	20.3%	0.03 [−0.27, 0.33]
Ibarra (medium) 2019	0.83	0.48	48	0.86	0.51	16	21.2%	−0.03 [−0.31, 0.25]
Ibarra (low) 2019	1.03	0.57	48	0.86	0.51	16	20.5%	0.17 [−0.13, 0.47]
**Subtotal (95% CI)**			**212**			**84**	**99.5%**	**−0.10 [−0.33, 0.13]**
Heterogeneity: Tau² = 0.04; Chi² = 10.95, df = 4 (P = 0.03); I² = 63%								
Test for overall effect: Z = 0.87 (P = 0.38)								
**2.14.2 Galacto-oligosaccharides**								
Schoemaker (high) 2022	14.7	8.3	44	16	8.3	22	0.2%	−1.30 [−5.55, 2.95]
Schoemaker (low) 2022	17	9	45	16	8.3	21	0.2%	1.00 [−3.42, 5.42]
**Subtotal (95% CI)**			**89**			**43**	**0.5%**	**−0.20 [−3.26, 2.87]**
Heterogeneity: Tau² = 0.00; Chi² = 0.54, df = 1 (P = 0.46); I² = 0%								
Test for overall effect: Z = 0.12 (P = 0.90)								
**Total (95% CI)**			**301**			**127**	**100.0%**	**−0.10 [−0.31, 0.11]**
Heterogeneity: Tau² = 0.03; Chi² = 11.49, df = 6 (P = 0.07); I² = 48%								
Test for overall effect: Z = 0.94 (P = 0.35)								
Test for subgroup differences: Chi² = 0.00, df = 1 (P = 0.95), I² = 0%								

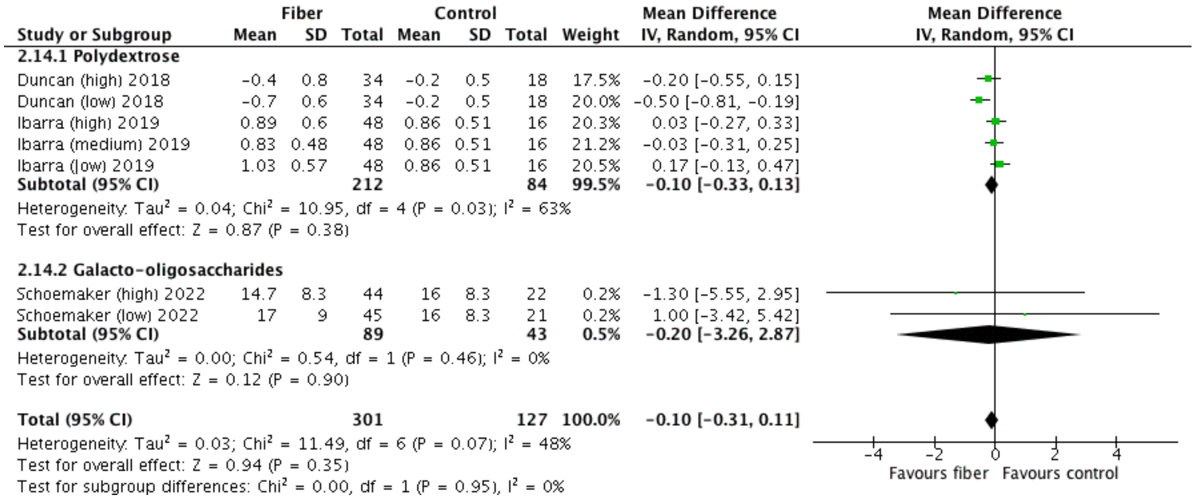

Supplementary Figure 9B Forest plot of subgroup analysis based on the type of fiber for PAC-SYM (global) in randomized controlled trials comparing fiber with control in adults with chronic constipation (n=428). Values were calculated as mean difference (95% CIs) using a random-effects model. Abbreviations: IV, inverse variance; PAC-SYM, Patient Assessment of Constipation Symptoms.

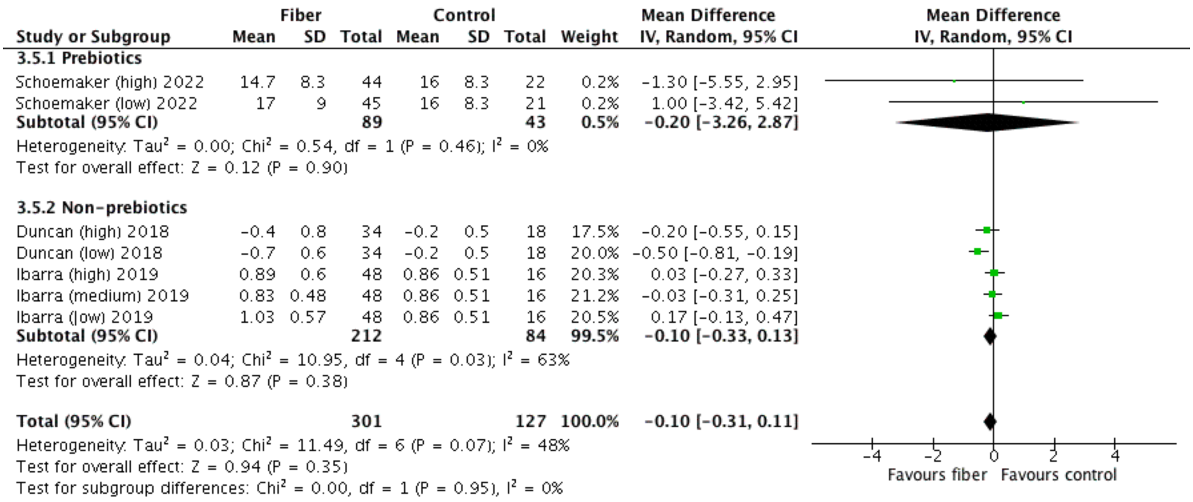

Supplementary Figure 9C Forest plot of subgroup analysis based on prebiotic status of the intervention for PAC-SYM (global) in randomized controlled trials comparing fiber with control in adults with chronic constipation (n=428). Values were calculated as mean difference (95% CIs) using a random-effects model. Abbreviations: IV, inverse variance; PAC-SYM, Patient Assessment of Constipation Symptoms.

Study or Subgroup	Fiber Mean	Fiber SD	Fiber Total	Control Mean	Control SD	Control Total	Weight	Mean Difference IV, Random, 95% CI
**5.19.1 Low dose (≤10 g/d)**								
Duncan (low) 2018	−0.7	0.6	34	−0.2	0.5	18	20.0%	−0.50 [−0.81, −0.19]
Ibarra (medium) 2019	0.83	0.48	48	0.86	0.51	16	21.2%	−0.03 [−0.31, 0.25]
Ibarra (low) 2019	1.03	0.57	48	0.86	0.51	16	20.5%	0.17 [−0.13, 0.47]
Schoemaker (low) 2022	17	9	45	16	8.3	21	0.2%	1.00 [−3.42, 5.42]
**Subtotal (95% CI)**			**175**			**71**	**62.0%**	**−0.11 [−0.47, 0.25]**
Heterogeneity: Tau² = 0.08; Chi² = 10.16, df = 3 (P = 0.02); I² = 70%								
Test for overall effect: Z = 0.59 (P = 0.55)								
**5.19.2 High dose (>10 g/d)**								
Duncan (high) 2018	−0.4	0.8	34	−0.2	0.5	18	17.5%	−0.20 [−0.55, 0.15]
Ibarra (high) 2019	0.89	0.6	48	0.86	0.51	16	20.3%	0.03 [−0.27, 0.33]
Schoemaker (high) 2022	14.7	8.3	44	16	8.3	22	0.2%	−1.30 [−5.55, 2.95]
**Subtotal (95% CI)**			**126**			**56**	**38.0%**	**−0.07 [−0.30, 0.16]**
Heterogeneity: Tau² = 0.00; Chi² = 1.26, df = 2 (P = 0.53); I² = 0%								
Test for overall effect: Z = 0.60 (P = 0.55)								
**Total (95% CI)**			**301**			**127**	**100.0%**	**−0.10 [−0.31, 0.11]**
Heterogeneity: Tau² = 0.03; Chi² = 11.49, df = 6 (P = 0.07); I² = 48%								
Test for overall effect: Z = 0.94 (P = 0.35)								
Test for subgroup differences: Chi² = 0.03, df = 1 (P = 0.86), I² = 0%								

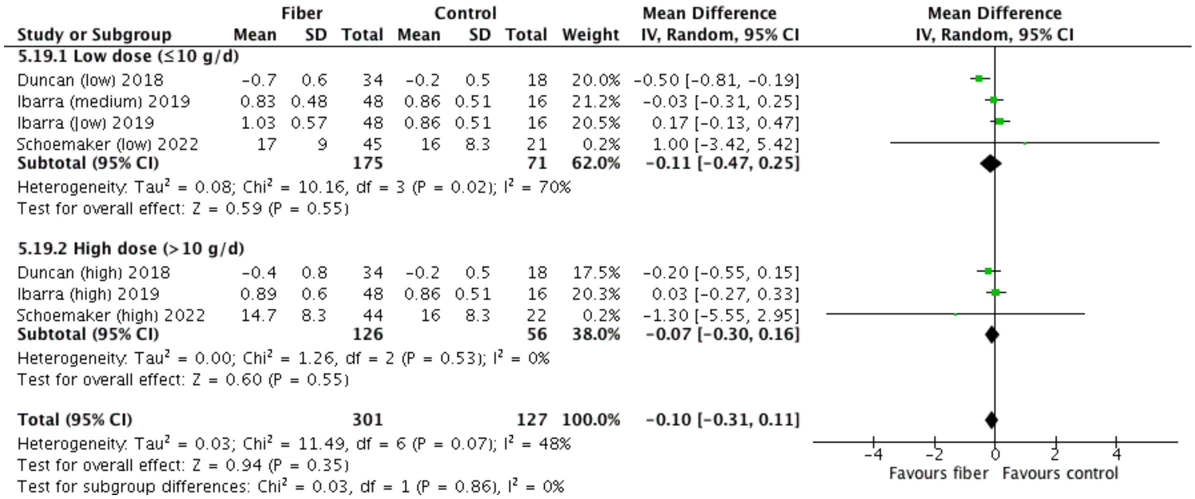

Supplementary Figure 9D Forest plot of subgroup analysis based on the dose administered for PAC-SYM (global) in randomized controlled trials comparing fiber with control in adults with chronic constipation (n=428). Values were calculated as mean difference (95% CIs) using a random-effects model. Abbreviations: IV, inverse variance; PAC-SYM, Patient Assessment of Constipation Symptoms.

Study or Subgroup	Fiber Mean	Fiber SD	Fiber Total	Control Mean	Control SD	Control Total	Weight	Mean Difference IV, Random, 95% CI
**4.16.1 Duration of treatment < 4 weeks**								
Ibarra (high) 2019	0.89	0.6	48	0.86	0.51	16	20.3%	0.03 [−0.27, 0.33]
Ibarra (medium) 2019	0.83	0.48	48	0.86	0.51	16	21.2%	−0.03 [−0.31, 0.25]
Ibarra (low) 2019	1.03	0.57	48	0.86	0.51	16	20.5%	0.17 [−0.13, 0.47]
Schoemaker (high) 2022	14.7	8.3	44	16	8.3	22	0.2%	−1.30 [−5.55, 2.95]
Schoemaker (low) 2022	17	9	45	16	8.3	21	0.2%	1.00 [−3.42, 5.42]
**Subtotal (95% CI)**			**233**			**91**	**62.5%**	**0.05 [−0.12, 0.22]**
Heterogeneity: Tau² = 0.00; Chi² = 1.51, df = 4 (P = 0.82); I² = 0%								
Test for overall effect: Z = 0.62 (P = 0.54)								
**4.16.2 Duration of treatment ≥ 4 weeks**								
Duncan (high) 2018	−0.4	0.8	34	−0.2	0.5	18	17.5%	−0.20 [−0.55, 0.15]
Duncan (low) 2018	−0.7	0.6	34	−0.2	0.5	18	20.0%	−0.50 [−0.81, −0.19]
**Subtotal (95% CI)**			**68**			**36**	**37.5%**	**−0.36 [−0.66, −0.07]**
Heterogeneity: Tau² = 0.02; Chi² = 1.57, df = 1 (P = 0.21); I² = 36%								
Test for overall effect: Z = 2.44 (P = 0.01)								
**Total (95% CI)**			**301**			**127**	**100.0%**	**−0.10 [−0.31, 0.11]**
Heterogeneity: Tau² = 0.03; Chi² = 11.49, df = 6 (P = 0.07); I² = 48%								
Test for overall effect: Z = 0.94 (P = 0.35)								
Test for subgroup differences: Chi² = 5.84, df = 1 (P = 0.02), I² = 82.9%								

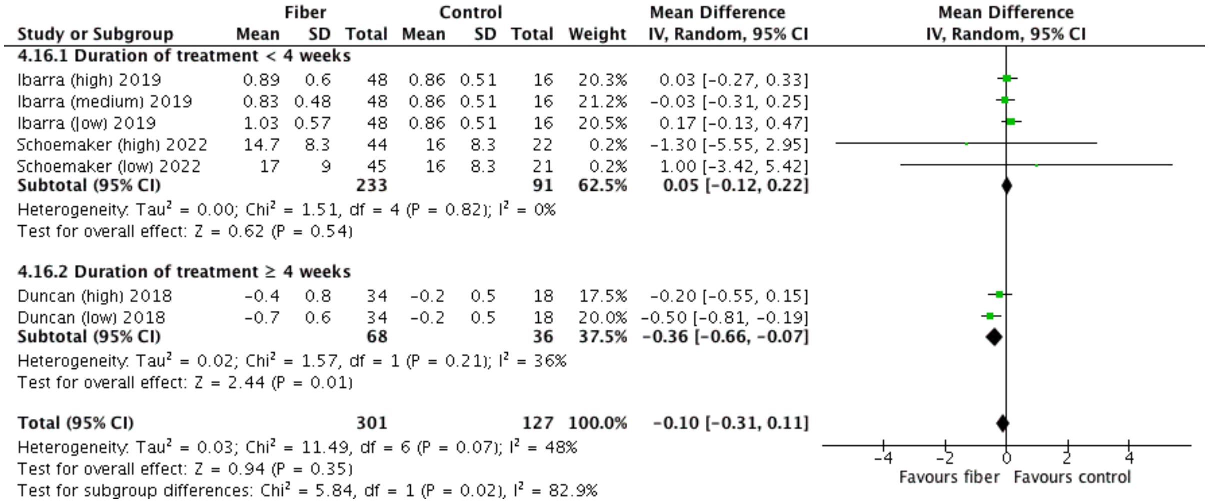

Supplementary Figure 9E Forest plot of subgroup analysis based on the treatment duration for PAC-SYM (global) in randomized controlled trials comparing fiber with control in adults with chronic constipation (n=428). Values were calculated as mean difference (95% CIs) using a random-effects model. Abbreviations: IV, inverse variance; PAC-SYM, Patient Assessment of Constipation Symptoms.

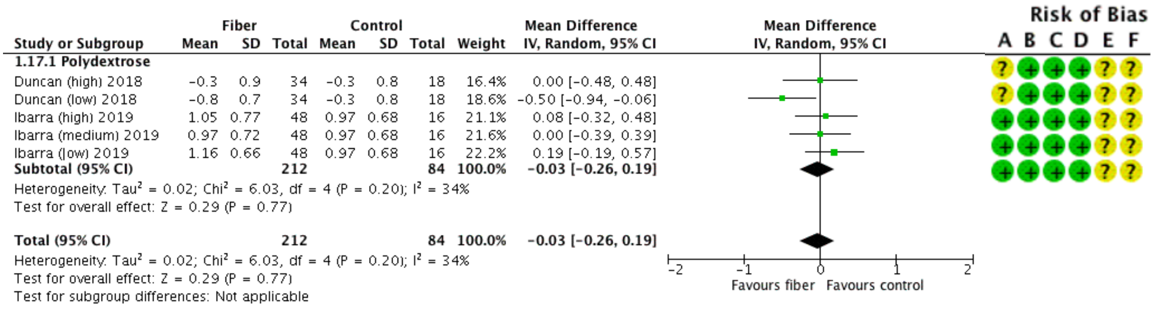

Supplementary Figure 10A Forest plot of PAC-SYM (abdominal) in randomized controlled trials comparing fiber with control in adults with chronic constipation (n=296). Values were calculated as mean difference (95% CIs) using a random-effects model. Risk of bias key: A=bias arising from the randomization process; B=bias arising from deviations from intended interventions; C=bias due to missing outcome data; D=bias in measurement of the outcome; E=bias in selection of the reported result; F=overall risk of bias. Abbreviations: IV, inverse variance; PAC-SYM, Patient Assessment of Constipation Symptoms.

Study or Subgroup	Fiber Mean	Fiber SD	Fiber Total	Control Mean	Control SD	Control Total	Weight	Mean Difference IV, Random, 95% CI
**5.20.1 Low dose (≤10 g/d)**								
Duncan (low) 2018	−0.8	0.7	34	−0.3	0.8	18	18.6%	−0.50 [−0.94, −0.06]
Ibarra (medium) 2019	0.97	0.72	48	0.97	0.68	16	21.6%	0.00 [−0.39, 0.39]
Ibarra (low) 2019	1.16	0.66	48	0.97	0.68	16	22.2%	0.19 [−0.19, 0.57]
**Subtotal (95% CI)**			**130**			**50**	**62.5%**	**−0.09 [−0.48, 0.30]**
Heterogeneity: Tau² = 0.08; Chi² = 5.60, df = 2 (P = 0.06); I² = 64%								
Test for overall effect: Z = 0.46 (P = 0.65)								
**5.20.2 High dose (>10 g/d)**								
Duncan (high) 2018	−0.3	0.9	34	−0.3	0.8	18	16.4%	0.00 [−0.48, 0.48]
Ibarra (high) 2019	1.05	0.77	48	0.97	0.68	16	21.1%	0.08 [−0.32, 0.48]
**Subtotal (95% CI)**			**82**			**34**	**37.5%**	**0.05 [−0.26, 0.35]**
Heterogeneity: Tau² = 0.00; Chi² = 0.06, df = 1 (P = 0.80); I² = 0%								
Test for overall effect: Z = 0.30 (P = 0.76)								
**Total (95% CI)**			**212**			**84**	**100.0%**	**−0.03 [−0.26, 0.19]**
Heterogeneity: Tau² = 0.02; Chi² = 6.03, df = 4 (P = 0.20); I² = 34%								
Test for overall effect: Z = 0.29 (P = 0.77)								
Test for subgroup differences: Chi² = 0.30, df = 1 (P = 0.58), I² = 0%								

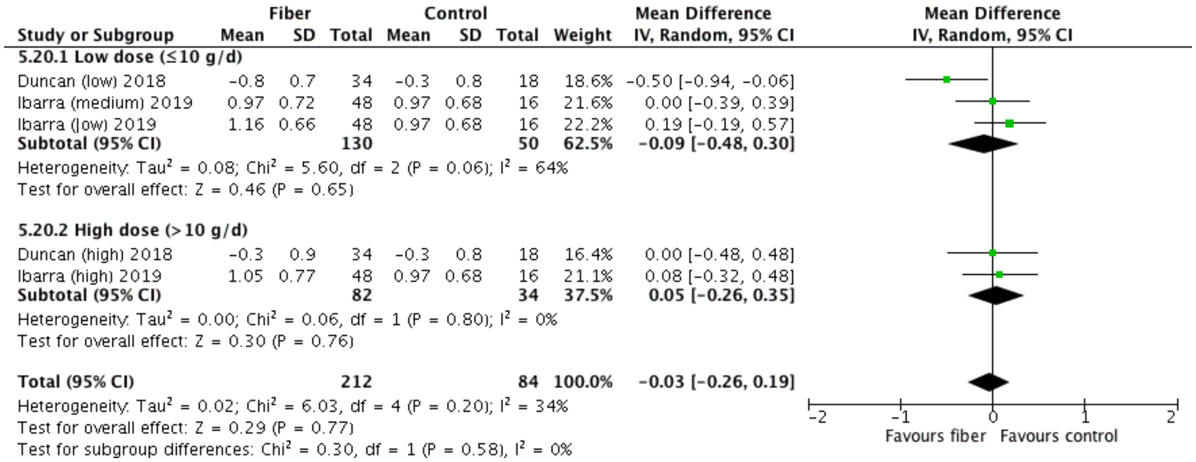

Supplementary Figure 10B Forest plot of subgroup analysis based on the dose administered for PAC-SYM (abdominal) in randomized controlled trials comparing fiber with control in adults with chronic constipation (n=296). Values were calculated as mean difference (95% CIs)) using a random-effects model. Abbreviations: IV, inverse variance; PAC-SYM, Patient Assessment of Constipation Symptoms.

Study or Subgroup	Fiber Mean	Fiber SD	Fiber Total	Control Mean	Control SD	Control Total	Weight	Mean Difference IV, Random, 95% CI
**4.17.1 Duration of treatment < 4 weeks**								
Ibarra (high) 2019	1.05	0.77	48	0.97	0.68	16	21.1%	0.08 [−0.32, 0.48]
Ibarra (medium) 2019	0.97	0.72	48	0.97	0.68	16	21.6%	0.00 [−0.39, 0.39]
Ibarra (low) 2019	1.16	0.66	48	0.97	0.68	16	22.2%	0.19 [−0.19, 0.57]
**Subtotal (95% CI)**			**144**			**48**	**65.0%**	**0.09 [−0.13, 0.32]**
Heterogeneity: Tau² = 0.00; Chi² = 0.47, df = 2 (P = 0.79); I² = 0%								
Test for overall effect: Z = 0.80 (P = 0.43)								
**4.17.2 Duration of treatment ≥ 4 weeks**								
Duncan (high) 2018	−0.3	0.9	34	−0.3	0.8	18	16.4%	0.00 [−0.48, 0.48]
Duncan (low) 2018	−0.8	0.7	34	−0.3	0.8	18	18.6%	−0.50 [−0.94, −0.06]
**Subtotal (95% CI)**			**68**			**36**	**35.0%**	**−0.26 [−0.75, 0.23]**
Heterogeneity: Tau² = 0.07; Chi² = 2.29, df = 1 (P = 0.13); I² = 56%								
Test for overall effect: Z = 1.04 (P = 0.30)								
**Total (95% CI)**			**212**			**84**	**100.0%**	**−0.03 [−0.26, 0.19]**
Heterogeneity: Tau² = 0.02; Chi² = 6.03, df = 4 (P = 0.20); I² = 34%								
Test for overall effect: Z = 0.29 (P = 0.77)								
Test for subgroup differences: Chi² = 1.63, df = 1 (P = 0.20), I² = 38.6%								

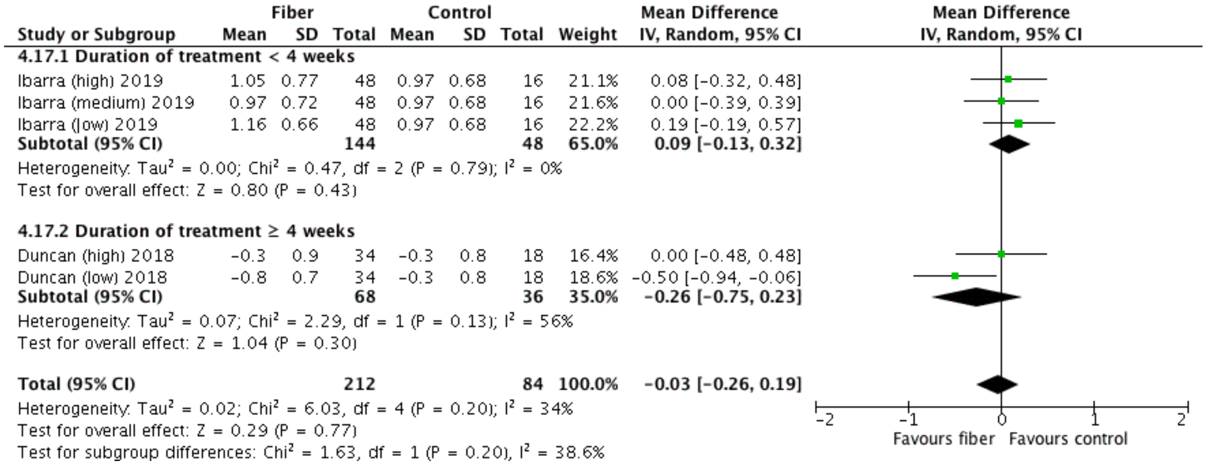

Supplementary Figure 10C Forest plot of subgroup analysis based on the duration of treatment for PAC-SYM (abdominal) in randomized controlled trials comparing fiber with control in adults with chronic constipation (n=296). Values were calculated as mean difference (95% CIs) using a random-effects model. Abbreviations: IV, inverse variance; PAC-SYM, Patient Assessment of Constipation Symptoms.

Study or Subgroup	Fiber Mean	Fiber SD	Fiber Total	Control Mean	Control SD	Control Total	Weight	Mean Difference IV, Random, 95% CI	A	B	C	D	E	F
**1.18.1 Polydextrose**														
Duncan (high) 2018	−0.3	0.8	34	−0.1	0.4	18	18.5%	−0.20 [−0.53, 0.13]	?	+	+	+	?	?
Duncan (low) 2018	−0.5	0.7	34	−0.1	0.4	18	19.9%	−0.40 [−0.70, −0.10]	?	+	+	+	?	?
Ibarra (high) 2019	0.43	0.46	48	0.37	0.5	16	21.1%	0.06 [−0.22, 0.34]	+	+	+	+	?	?
Ibarra (medium) 2019	0.33	0.42	48	0.37	0.5	16	21.4%	−0.04 [−0.31, 0.23]	+	+	+	+	?	?
Ibarra (low) 2019	0.61	0.71	48	0.37	0.5	16	19.0%	0.24 [−0.08, 0.56]	+	+	+	+	?	?
**Subtotal (95% CI)**			**212**			**84**	**100.0%**	**−0.07 [−0.28, 0.14]**						
Heterogeneity: Tau² = 0.03; Chi² = 9.85, df = 4 (P = 0.04); I² = 59%														
Test for overall effect: Z = 0.63 (P = 0.53)														
**Total (95% CI)**			**212**			**84**	**100.0%**	**−0.07 [−0.28, 0.14]**						
Heterogeneity: Tau² = 0.03; Chi² = 9.85, df = 4 (P = 0.04); I² = 59%														
Test for overall effect: Z = 0.63 (P = 0.53)														
Test for subgroup differences: Not applicable														

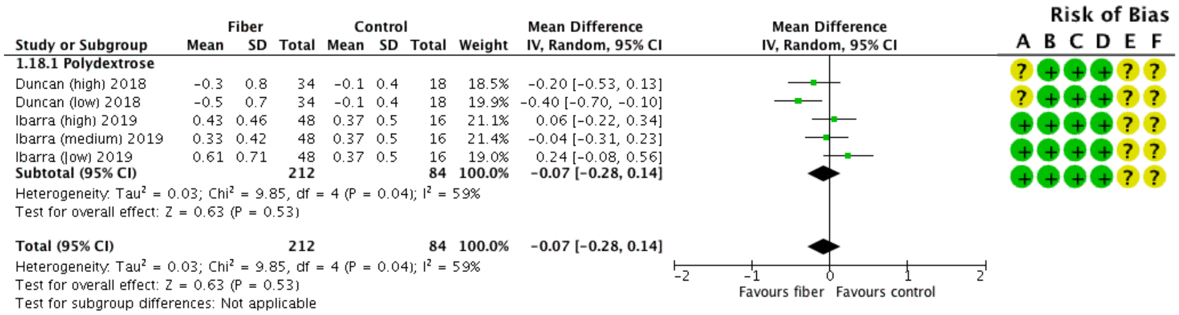

Supplementary Figure 11A Forest plot of PAC-SYM (rectal) in randomized controlled trials comparing fiber with control in adults with chronic constipation (n=296). Values were calculated as mean difference (95% CIs) using a random-effects model. Risk of bias key: A=bias arising from the randomization process; B=bias arising from deviations from intended interventions; C=bias due to missing outcome data; D=bias in measurement of the outcome; E=bias in selection of the reported result; F=overall risk of bias. Abbreviations: IV, inverse variance; PAC-SYM, Patient Assessment of Constipation Symptoms.

Study or Subgroup	Fiber Mean	Fiber SD	Fiber Total	Control Mean	Control SD	Control Total	Weight	Mean Difference IV, Random, 95% CI
**5.22.1 Low dose (≤10 g/d)**								
Duncan (low) 2018	−0.5	0.7	34	−0.1	0.4	18	19.9%	−0.40 [−0.70, −0.10]
Ibarra (medium) 2019	0.33	0.42	48	0.37	0.5	16	21.4%	−0.04 [−0.31, 0.23]
Ibarra (low) 2019	0.61	0.71	48	0.37	0.5	16	19.0%	0.24 [−0.08, 0.56]
**Subtotal (95% CI)**			**130**			**50**	**60.4%**	**−0.07 [−0.42, 0.28]**
Heterogeneity: $Tau^2 = 0.07$; $Chi^2 = 8.39$, df = 2 (P = 0.02); $I^2 = 76\%$								
Test for overall effect: Z = 0.39 (P = 0.70)								
**5.22.2 High dose (>10 g/d)**								
Duncan (high) 2018	−0.3	0.8	34	−0.1	0.4	18	18.5%	−0.20 [−0.53, 0.13]
Ibarra (high) 2019	0.43	0.46	48	0.37	0.5	16	21.1%	0.06 [−0.22, 0.34]
**Subtotal (95% CI)**			**82**			**34**	**39.6%**	**−0.06 [−0.31, 0.20]**
Heterogeneity: $Tau^2 = 0.01$; $Chi^2 = 1.42$, df = 1 (P = 0.23); $I^2 = 29\%$								
Test for overall effect: Z = 0.43 (P = 0.67)								
**Total (95% CI)**			**212**			**84**	**100.0%**	**−0.07 [−0.28, 0.14]**
Heterogeneity: $Tau^2 = 0.03$; $Chi^2 = 9.85$, df = 4 (P = 0.04); $I^2 = 59\%$								
Test for overall effect: Z = 0.63 (P = 0.53)								
Test for subgroup differences: $Chi^2 = 0.00$, df = 1 (P = 0.95), $I^2 = 0\%$								

Mean Difference IV, Random, 95% CI

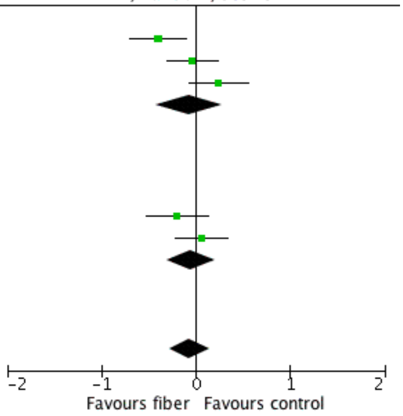

Supplementary Figure 11B Forest plot of subgroup analysis based on the dose administered for PAC-SYM (rectal) in randomized controlled trials comparing fiber with control in adults with chronic constipation (n=296). Values were calculated as mean difference (95% CIs) using a random-effects model. Abbreviations: IV, inverse variance; PAC-SYM, Patient Assessment of Constipation Symptoms.

Study or Subgroup	Fiber Mean	Fiber SD	Fiber Total	Control Mean	Control SD	Control Total	Weight	Mean Difference IV, Random, 95% CI
**4.18.1 Duration of treatment < 4 weeks**								
Ibarra (high) 2019	0.43	0.46	48	0.37	0.5	16	21.1%	0.06 [−0.22, 0.34]
Ibarra (medium) 2019	0.33	0.42	48	0.37	0.5	16	21.4%	−0.04 [−0.31, 0.23]
Ibarra (low) 2019	0.61	0.71	48	0.37	0.5	16	19.0%	0.24 [−0.08, 0.56]
**Subtotal (95% CI)**			**144**			**48**	**61.6%**	**0.07 [−0.09, 0.24]**
Heterogeneity: Tau² = 0.00; Chi² = 1.74, df = 2 (P = 0.42); I² = 0%								
Test for overall effect: Z = 0.85 (P = 0.39)								
**4.18.2 Duration of treatment ≥ 4 weeks**								
Duncan (high) 2018	−0.3	0.8	34	−0.1	0.4	18	18.5%	−0.20 [−0.53, 0.13]
Duncan (low) 2018	−0.5	0.7	34	−0.1	0.4	18	19.9%	−0.40 [−0.70, −0.10]
**Subtotal (95% CI)**			**68**			**36**	**38.4%**	**−0.31 [−0.53, −0.09]**
Heterogeneity: Tau² = 0.00; Chi² = 0.78, df = 1 (P = 0.38); I² = 0%								
Test for overall effect: Z = 2.74 (P = 0.006)								
**Total (95% CI)**			**212**			**84**	**100.0%**	**−0.07 [−0.28, 0.14]**
Heterogeneity: Tau² = 0.03; Chi² = 9.85, df = 4 (P = 0.04); I² = 59%								
Test for overall effect: Z = 0.63 (P = 0.53)								
Test for subgroup differences: Chi² = 7.33, df = 1 (P = 0.007), I² = 86.3%								

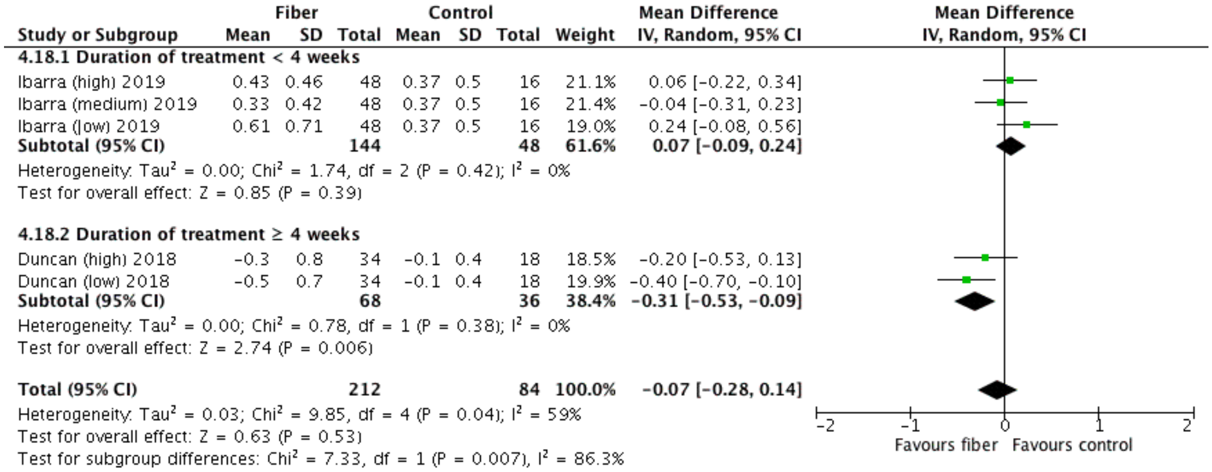

Supplementary Figure 11C Forest plot of subgroup analysis based on the duration of treatment for PAC-SYM (rectal) in randomized controlled trials comparing fiber with control in adults with chronic constipation (n=296). Values were calculated as mean difference (95% CIs) using a random-effects model. Abbreviations: IV, inverse variance; PAC-SYM, Patient Assessment of Constipation Symptoms.

Study or Subgroup	Fiber Mean	Fiber SD	Fiber Total	Control Mean	Control SD	Control Total	Weight	Mean Difference IV, Random, 95% CI
**1.19.1 Polydextrose**								
Duncan (high) 2018	−0.4	1	34	−0.2	0.8	18	17.5%	−0.20 [−0.70, 0.30]
Duncan (low) 2018	−0.8	0.9	34	−0.2	0.8	18	18.7%	−0.60 [−1.08, −0.12]
Ibarra (high) 2019	1.19	0.84	48	1.23	0.78	16	20.5%	−0.04 [−0.49, 0.41]
Ibarra (medium) 2019	1.2	0.76	48	1.23	0.78	16	21.3%	−0.03 [−0.47, 0.41]
Ibarra (low) 2019	1.34	0.69	48	1.23	0.78	16	22.0%	0.11 [−0.32, 0.54]
**Subtotal (95% CI)**			**212**			**84**	**100.0%**	**−0.14 [−0.37, 0.10]**
Heterogeneity: Tau² = 0.02; Chi² = 5.35, df = 4 (P = 0.25); I² = 25%								
Test for overall effect: Z = 1.14 (P = 0.25)								
**Total (95% CI)**			**212**			**84**	**100.0%**	**−0.14 [−0.37, 0.10]**
Heterogeneity: Tau² = 0.02; Chi² = 5.35, df = 4 (P = 0.25); I² = 25%								
Test for overall effect: Z = 1.14 (P = 0.25)								
Test for subgroup differences: Not applicable								

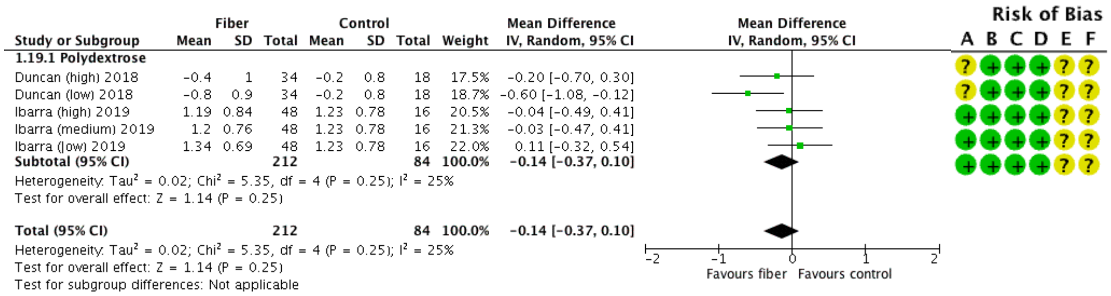

Supplementary Figure 12A Forest plot of PAC-SYM (stool) in randomized controlled trials comparing fiber with control in adults with chronic constipation (n=296). Values were calculated as mean difference (95% CIs) using a random-effects model. Risk of bias key: A=bias arising from the randomization process; B=bias arising from deviations from intended interventions; C=bias due to missing outcome data; D=bias in measurement of the outcome; E=bias in selection of the reported result; F=overall risk of bias. Abbreviations: IV, inverse variance; PAC-SYM, Patient Assessment of Constipation Symptoms.

Study or Subgroup	Fiber Mean	Fiber SD	Fiber Total	Control Mean	Control SD	Control Total	Weight	Mean Difference IV, Random, 95% CI
**5.23.1 Low dose (≤10 g/d)**								
Duncan (low) 2018	−0.8	0.9	34	−0.2	0.8	18	18.7%	−0.60 [−1.08, −0.12]
Ibarra (medium) 2019	1.2	0.76	48	1.23	0.78	16	21.3%	−0.03 [−0.47, 0.41]
Ibarra (low) 2019	1.34	0.69	48	1.23	0.78	16	22.0%	0.11 [−0.32, 0.54]
**Subtotal (95% CI)**			**130**			**50**	**62.0%**	**−0.16 [−0.58, 0.25]**
Heterogeneity: Tau² = 0.08; Chi² = 5.11, df = 2 (P = 0.08); I² = 61%								
Test for overall effect: Z = 0.77 (P = 0.44)								
**5.23.2 High dose (>10 g/d)**								
Duncan (high) 2018	−0.4	1	34	−0.2	0.8	18	17.5%	−0.20 [−0.70, 0.30]
Ibarra (high) 2019	1.19	0.84	48	1.23	0.78	16	20.5%	−0.04 [−0.49, 0.41]
**Subtotal (95% CI)**			**82**			**34**	**38.0%**	**−0.11 [−0.45, 0.22]**
Heterogeneity: Tau² = 0.00; Chi² = 0.22, df = 1 (P = 0.64); I² = 0%								
Test for overall effect: Z = 0.65 (P = 0.51)								
**Total (95% CI)**			**212**			**84**	**100.0%**	**−0.14 [−0.37, 0.10]**
Heterogeneity: Tau² = 0.02; Chi² = 5.35, df = 4 (P = 0.25); I² = 25%								
Test for overall effect: Z = 1.14 (P = 0.25)								
Test for subgroup differences: Chi² = 0.03, df = 1 (P = 0.85), I² = 0%								

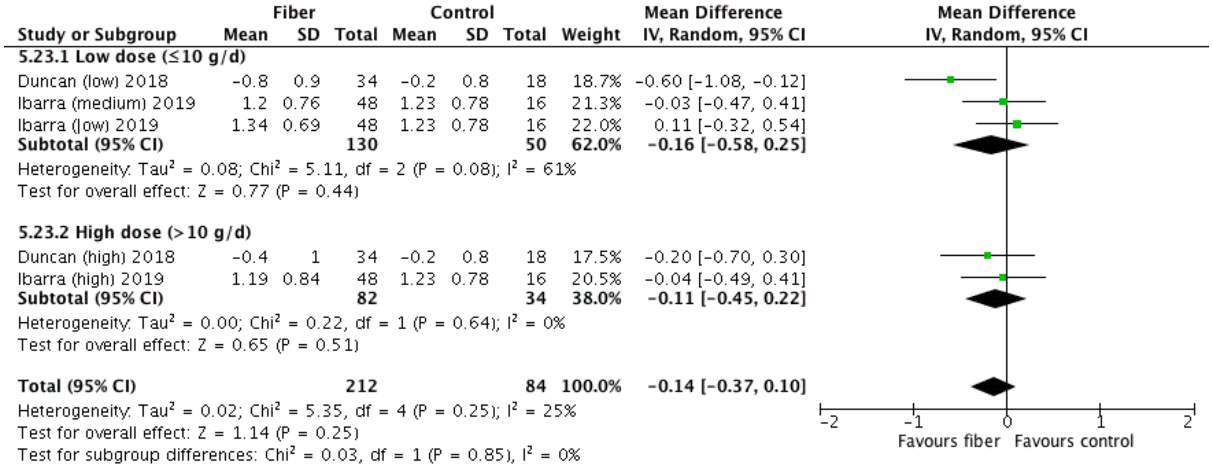

Supplementary Figure 12B Forest plot of subgroup analysis based on the dose administered for PAC-SYM (stool) in randomized controlled trials comparing fiber with control in adults with chronic constipation (n=296). Values were calculated as mean difference (95% CIs) using a random-effects model. Abbreviations: IV, inverse variance; PAC-SYM, Patient Assessment of Constipation Symptoms.

Study or Subgroup	Fiber Mean	Fiber SD	Fiber Total	Control Mean	Control SD	Control Total	Weight	Mean Difference IV, Random, 95% CI
**4.19.1 Duration of treatment < 4 weeks**								
Ibarra (high) 2019	1.19	0.84	48	1.23	0.78	16	20.5%	−0.04 [−0.49, 0.41]
Ibarra (medium) 2019	1.2	0.76	48	1.23	0.78	16	21.3%	−0.03 [−0.47, 0.41]
Ibarra (low) 2019	1.34	0.69	48	1.23	0.78	16	22.0%	0.11 [−0.32, 0.54]
**Subtotal (95% CI)**			**144**			**48**	**63.8%**	**0.02 [−0.24, 0.27]**
Heterogeneity: Tau² = 0.00; Chi² = 0.29, df = 2 (P = 0.87); I² = 0%								
Test for overall effect: Z = 0.12 (P = 0.90)								
**4.19.2 Duration of treatment ≥ 4 weeks**								
Duncan (high) 2018	−0.4	1	34	−0.2	0.8	18	17.5%	−0.20 [−0.70, 0.30]
Duncan (low) 2018	−0.8	0.9	34	−0.2	0.8	18	18.7%	−0.60 [−1.08, −0.12]
**Subtotal (95% CI)**			**68**			**36**	**36.2%**	**−0.41 [−0.80, −0.02]**
Heterogeneity: Tau² = 0.02; Chi² = 1.29, df = 1 (P = 0.26); I² = 22%								
Test for overall effect: Z = 2.04 (P = 0.04)								
**Total (95% CI)**			**212**			**84**	**100.0%**	**−0.14 [−0.37, 0.10]**
Heterogeneity: Tau² = 0.02; Chi² = 5.35, df = 4 (P = 0.25); I² = 25%								
Test for overall effect: Z = 1.14 (P = 0.25)								
Test for subgroup differences: Chi² = 3.15, df = 1 (P = 0.08), I² = 68.3%								

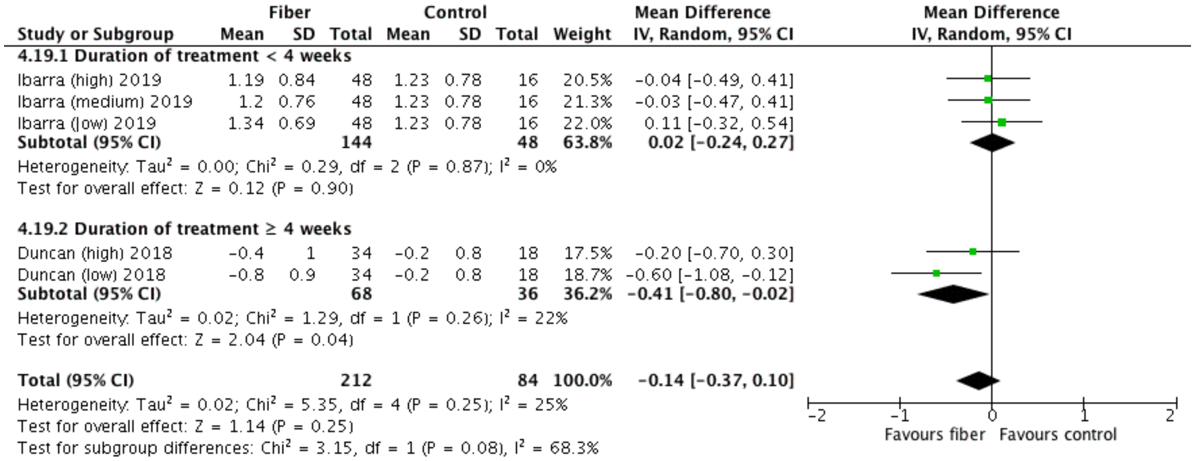

Supplementary Figure 12C Forest plot of subgroup analysis based on the treatment duration for PAC-SYM (stool) in randomized controlled trials comparing fiber with control in adults with chronic constipation (n=296). Values were calculated as mean difference (95% CIs) using a random-effects model. Abbreviations: IV, inverse variance; PAC-SYM, Patient Assessment of Constipation Symptoms.

Severity of straining

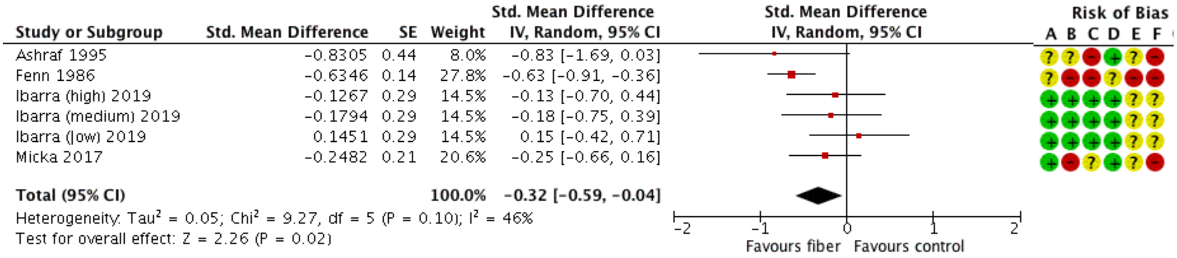

Supplementary Figure 13A Forest plot of severity of straining in randomized controlled trials comparing fiber with control in adults with chronic constipation (n=498). Values were calculated as standardized mean difference (95% CIs) using a random-effects model. Risk of bias key: A=bias arising from the randomization process; B=bias arising from deviations from intended interventions; C=bias due to missing outcome data; D=bias in measurement of the outcome; E=bias in selection of the reported result; F=overall risk of bias. Abbreviation: IV, inverse variance.

Study or Subgroup	Std. Mean Difference	SE	Weight	Std. Mean Difference IV, Random, 95% CI
**3.11.1 Prebiotics**				
Micka 2017	−0.2482	0.21	20.6%	−0.25 [−0.66, 0.16]
**Subtotal (95% CI)**			**20.6%**	**−0.25 [−0.66, 0.16]**
Heterogeneity: Not applicable				
Test for overall effect: Z = 1.18 (P = 0.24)				
**3.11.2 Non-prebiotics**				
Ashraf 1995	−0.8305	0.44	8.0%	−0.83 [−1.69, 0.03]
Fenn 1986	−0.6346	0.14	27.8%	−0.63 [−0.91, −0.36]
Ibarra (high) 2019	−0.1267	0.29	14.5%	−0.13 [−0.70, 0.44]
Ibarra (medium) 2019	−0.1794	0.29	14.5%	−0.18 [−0.75, 0.39]
Ibarra (low) 2019	0.1451	0.29	14.5%	0.15 [−0.42, 0.71]
**Subtotal (95% CI)**			**79.4%**	**−0.32 [−0.67, 0.02]**
Heterogeneity: Tau^2 = 0.08; Chi^2 = 8.73, df = 4 (P = 0.07); I^2 = 54%				
Test for overall effect: Z = 1.83 (P = 0.07)				
**Total (95% CI)**			**100.0%**	**−0.32 [−0.59, −0.04]**
Heterogeneity: Tau^2 = 0.05; Chi^2 = 9.27, df = 5 (P = 0.10); I^2 = 46%				
Test for overall effect: Z = 2.26 (P = 0.02)				
Test for subgroup differences: Chi^2 = 0.08, df = 1 (P = 0.78), I^2 = 0%				

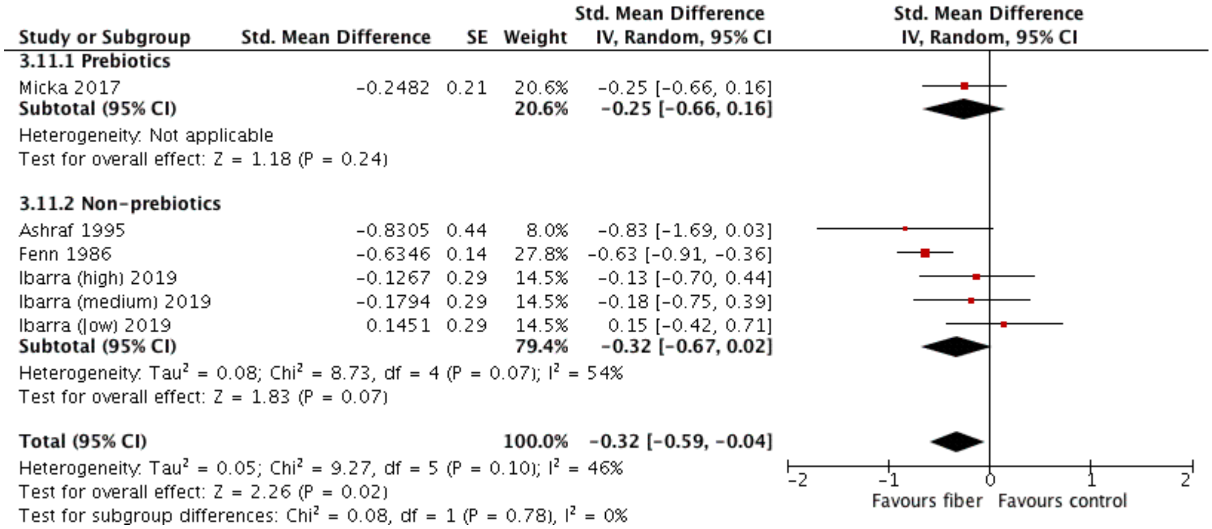

Supplementary Figure 13B Forest plot of subgroup analysis based on prebiotic status of the intervention for severity of straining in randomized controlled trials comparing fiber with control in adults with chronic constipation (n=498). Values were calculated as standardized mean difference (95% CIs) using a random-effects model. Abbreviation: IV, inverse variance.

Study or Subgroup	Std. Mean Difference	SE	Weight	Std. Mean Difference IV, Random, 95% CI
**5.12.1 Low dose (≤10 g/d)**				
Ibarra (medium) 2019	−0.1794	0.29	14.5%	−0.18 [−0.75, 0.39]
Ibarra (low) 2019	0.1451	0.29	14.5%	0.15 [−0.42, 0.71]
**Subtotal (95% CI)**			**29.1%**	**−0.02 [−0.42, 0.38]**
Heterogeneity: Tau² = 0.00; Chi² = 0.63, df = 1 (P = 0.43); I² = 0%				
Test for overall effect: Z = 0.08 (P = 0.93)				
**5.12.2 High dose (>10 g/d)**				
Ashraf 1995	−0.8305	0.44	8.0%	−0.83 [−1.69, 0.03]
Fenn 1986	−0.6346	0.14	27.8%	−0.63 [−0.91, −0.36]
Ibarra (high) 2019	−0.1267	0.29	14.5%	−0.13 [−0.70, 0.44]
Micka 2017	−0.2482	0.21	20.6%	−0.25 [−0.66, 0.16]
**Subtotal (95% CI)**			**70.9%**	**−0.45 [−0.73, −0.16]**
Heterogeneity: Tau² = 0.03; Chi² = 4.56, df = 3 (P = 0.21); I² = 34%				
Test for overall effect: Z = 3.10 (P = 0.002)				
**Total (95% CI)**			**100.0%**	**−0.32 [−0.59, −0.04]**
Heterogeneity: Tau² = 0.05; Chi² = 9.27, df = 5 (P = 0.10); I² = 46%				
Test for overall effect: Z = 2.26 (P = 0.02)				
Test for subgroup differences: Chi² = 2.95, df = 1 (P = 0.09), I² = 66.1%				

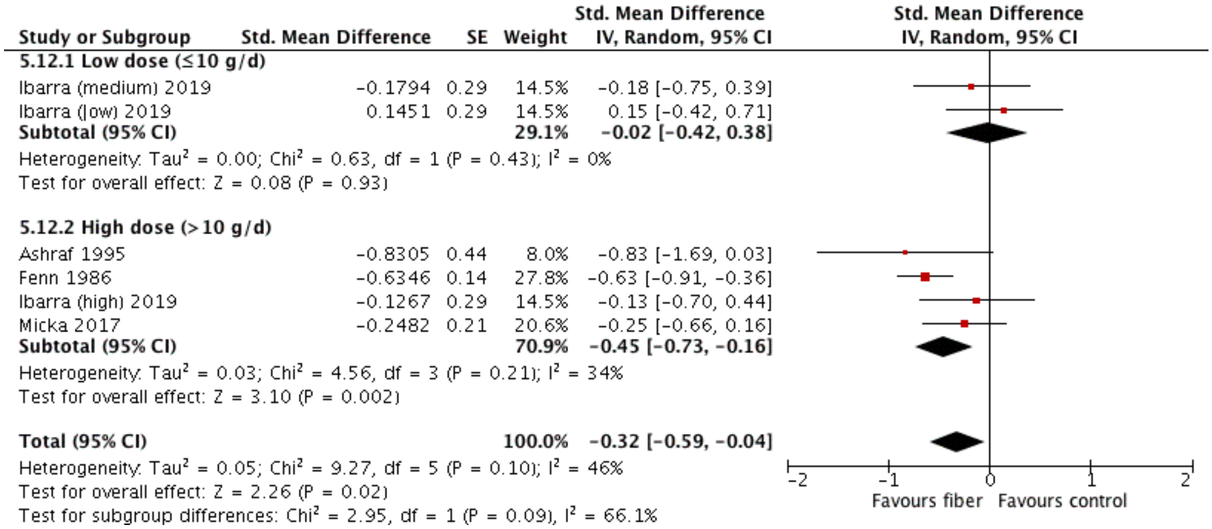

Supplementary Figure 13C Forest plot of subgroup analysis based on the dose administered for severity of straining in randomized controlled trials comparing fiber with control in adults with chronic constipation (n=498). Values were calculated as standardized mean difference (95% CIs) using a random-effects model. Abbreviation: IV, inverse variance.

Study or Subgroup	Std. Mean Difference	SE	Weight	Std. Mean Difference IV, Random, 95% CI
**4.9.1 Duration of treatment < 4 weeks**				
Fenn 1986	−0.6346	0.14	27.8%	−0.63 [−0.91, −0.36]
Ibarra (high) 2019	−0.1267	0.29	14.5%	−0.13 [−0.70, 0.44]
Ibarra (medium) 2019	−0.1794	0.29	14.5%	−0.18 [−0.75, 0.39]
Ibarra (low) 2019	0.1451	0.29	14.5%	0.15 [−0.42, 0.71]
**Subtotal (95% CI)**			**71.3%**	**−0.25 [−0.64, 0.13]**
Heterogeneity: Tau² = 0.09; Chi² = 7.81, df = 3 (P = 0.05); I² = 62%				
Test for overall effect: Z = 1.29 (P = 0.20)				
**4.9.2 Duration of treatment ≥ 4 weeks**				
Ashraf 1995	−0.8305	0.44	8.0%	−0.83 [−1.69, 0.03]
Micka 2017	−0.2482	0.21	20.6%	−0.25 [−0.66, 0.16]
**Subtotal (95% CI)**			**28.7%**	**−0.41 [−0.92, 0.10]**
Heterogeneity: Tau² = 0.05; Chi² = 1.43, df = 1 (P = 0.23); I² = 30%				
Test for overall effect: Z = 1.57 (P = 0.12)				
**Total (95% CI)**			**100.0%**	**−0.32 [−0.59, −0.04]**
Heterogeneity: Tau² = 0.05; Chi² = 9.27, df = 5 (P = 0.10); I² = 46%				
Test for overall effect: Z = 2.26 (P = 0.02)				
Test for subgroup differences: Chi² = 0.23, df = 1 (P = 0.63), I² = 0%				

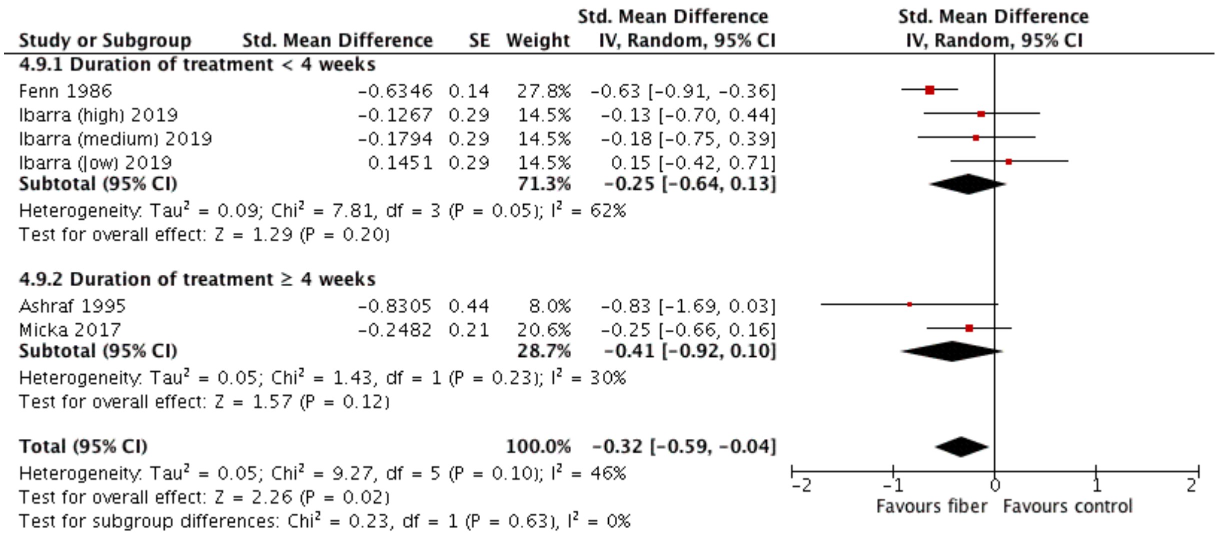

Supplementary Figure 13D Forest plot of subgroup analysis based on the treatment duration for severity of straining in randomized controlled trials comparing fiber with control in adults with chronic constipation (n=498). Values were calculated as standardized mean difference (95% CIs) using a random-effects model. Abbreviation: IV, inverse variance.

Severity of flatulence

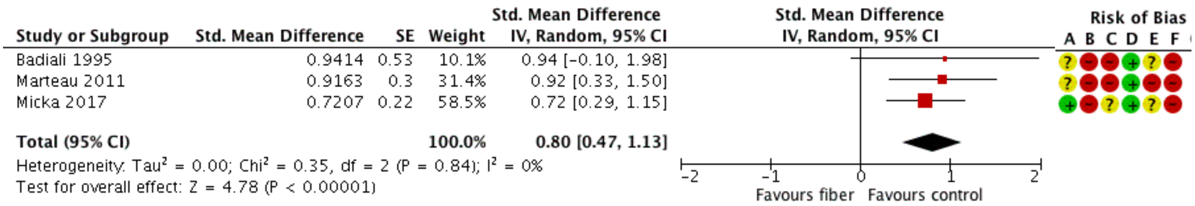

Supplementary Figure 14A Forest plot of severity of flatulence in randomized controlled trials comparing fiber with control in adults with chronic constipation (n=153). Values were calculated as standardized mean difference (95% CIs) using a random-effects model. Risk of bias key: A=bias arising from the randomization process; B=bias arising from deviations from intended interventions; C=bias due to missing outcome data; D=bias in measurement of the outcome; E=bias in selection of the reported result; F=overall risk of bias. Abbreviation: IV, inverse variance.

Study or Subgroup	Std. Mean Difference	SE	Weight	Std. Mean Difference IV, Random, 95% CI
**2.16.1 Inulin type fructans**				
Marteau 2011	0.9163	0.3	31.4%	0.92 [0.33, 1.50]
Micka 2017	0.7207	0.22	58.5%	0.72 [0.29, 1.15]
**Subtotal (95% CI)**			**89.9%**	**0.79 [0.44, 1.14]**
Heterogeneity: $Tau^2 = 0.00$; $Chi^2 = 0.28$, df = 1 (P = 0.60); $I^2 = 0\%$				
Test for overall effect: Z = 4.45 (P < 0.00001)				
**2.16.2 Wheat bran**				
Badiali 1995	0.9414	0.53	10.1%	0.94 [−0.10, 1.98]
**Subtotal (95% CI)**			**10.1%**	**0.94 [−0.10, 1.98]**
Heterogeneity: Not applicable				
Test for overall effect: Z = 1.78 (P = 0.08)				
**Total (95% CI)**			**100.0%**	**0.80 [0.47, 1.13]**
Heterogeneity: $Tau^2 = 0.00$; $Chi^2 = 0.35$, df = 2 (P = 0.84); $I^2 = 0\%$				
Test for overall effect: Z = 4.78 (P < 0.00001)				
Test for subgroup differences: $Chi^2 = 0.07$, df = 1 (P = 0.79), $I^2 = 0\%$				

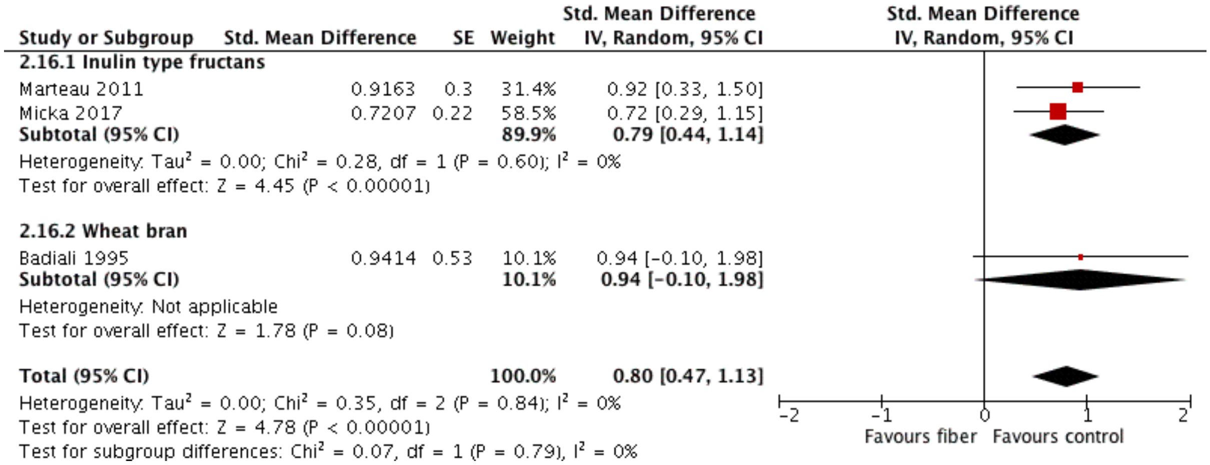

Supplementary Figure 14B Forest plot of subgroup analysis based on the type of fiber for severity of flatulence in randomized controlled trials comparing fiber with control in adults with chronic constipation (n=153). Values were calculated as standardized mean difference (95% CIs) using a random-effects model. Abbreviation: IV, inverse variance.

Study or Subgroup	Std. Mean Difference	SE	Weight	Std. Mean Difference IV, Random, 95% CI
**3.14.1 Prebiotics**				
Marteau 2011	0.9163	0.3	31.4%	0.92 [0.33, 1.50]
Micka 2017	0.7207	0.22	58.5%	0.72 [0.29, 1.15]
**Subtotal (95% CI)**			**89.9%**	**0.79 [0.44, 1.14]**
Heterogeneity: Tau² = 0.00; Chi² = 0.28, df = 1 (P = 0.60); I² = 0%				
Test for overall effect: Z = 4.45 (P < 0.00001)				
**3.14.2 Non-prebiotics**				
Badiali 1995	0.9414	0.53	10.1%	0.94 [-0.10, 1.98]
**Subtotal (95% CI)**			**10.1%**	**0.94 [-0.10, 1.98]**
Heterogeneity: Not applicable				
Test for overall effect: Z = 1.78 (P = 0.08)				
**Total (95% CI)**			**100.0%**	**0.80 [0.47, 1.13]**
Heterogeneity: Tau² = 0.00; Chi² = 0.35, df = 2 (P = 0.84); I² = 0%				
Test for overall effect: Z = 4.78 (P < 0.00001)				
Test for subgroup differences: Chi² = 0.07, df = 1 (P = 0.79), I² = 0%				

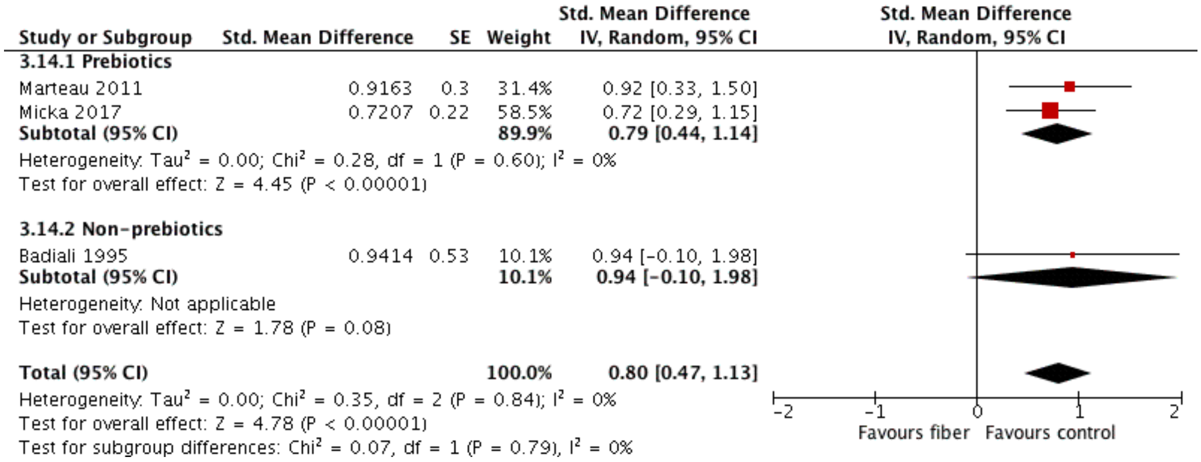

Supplementary Figure 14C Forest plot of subgroup analysis based on prebiotic status of the intervention for severity of flatulence in randomized controlled trials comparing fiber with control in adults with chronic constipation (n=153). Values were calculated as standardized mean difference (95% CIs) using a random-effects model. Abbreviation: IV, inverse variance.

Severity of incomplete evacuation

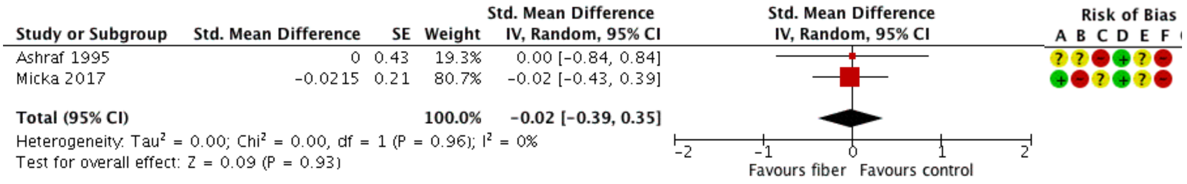

Supplementary Figure 15A Forest plot of severity of incomplete evacuation in randomized controlled trials comparing fiber with control in adults with chronic constipation (n=110). Values were calculated as standardized mean difference (95% CIs) using a random-effects model. Risk of bias key: A=bias arising from the randomization process; B=bias arising from deviations from intended interventions; C=bias due to missing outcome data; D=bias in measurement of the outcome; E=bias in selection of the reported result; F=overall risk of bias. Abbreviation: IV, inverse variance.

Study or Subgroup	Std. Mean Difference	SE	Weight	Std. Mean Difference IV, Random, 95% CI
**1.31.1 Psyllium**				
Ashraf 1995	0	0.43	19.3%	0.00 [−0.84, 0.84]
**Subtotal (95% CI)**			**19.3%**	**0.00 [−0.84, 0.84]**
Heterogeneity: Not applicable				
Test for overall effect: Z = 0.00 (P = 1.00)				
**1.31.2 Inulin-type fructans**				
Micka 2017	−0.0215	0.21	80.7%	−0.02 [−0.43, 0.39]
**Subtotal (95% CI)**			**80.7%**	**−0.02 [−0.43, 0.39]**
Heterogeneity: Not applicable				
Test for overall effect: Z = 0.10 (P = 0.92)				
**Total (95% CI)**			**100.0%**	**−0.02 [−0.39, 0.35]**
Heterogeneity: Tau² = 0.00; Chi² = 0.00, df = 1 (P = 0.96); I² = 0%				
Test for overall effect: Z = 0.09 (P = 0.93)				
Test for subgroup differences: Chi² = 0.00, df = 1 (P = 0.96), I² = 0%				

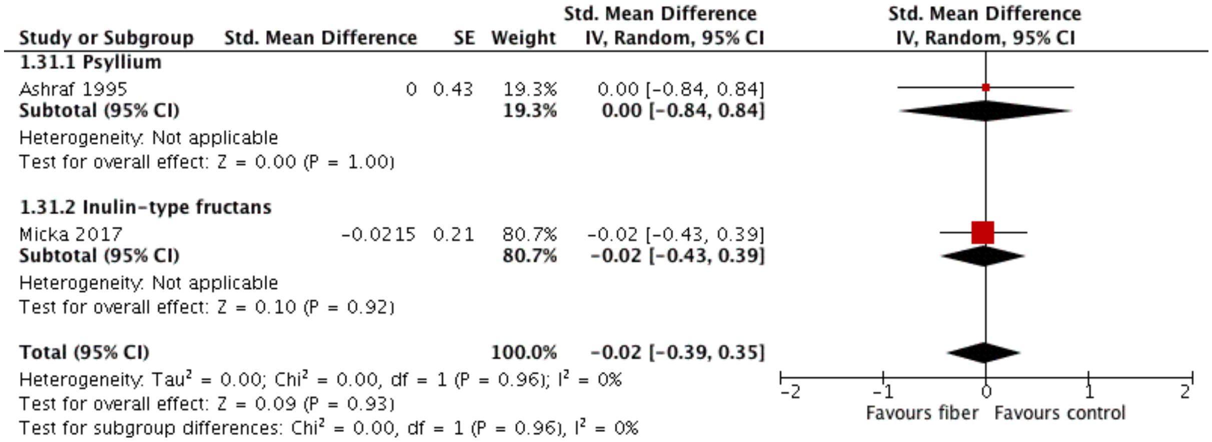

Supplementary Figure 15B Forest plot of subgroup analysis based on the type of fiber for severity of incomplete evacuation in randomized controlled trials comparing fiber with control in adults with chronic constipation (n=110). Values were calculated as standardized mean difference (95% CIs) using a random-effects model. Abbreviation: IV, inverse variance.

Severity of bloating

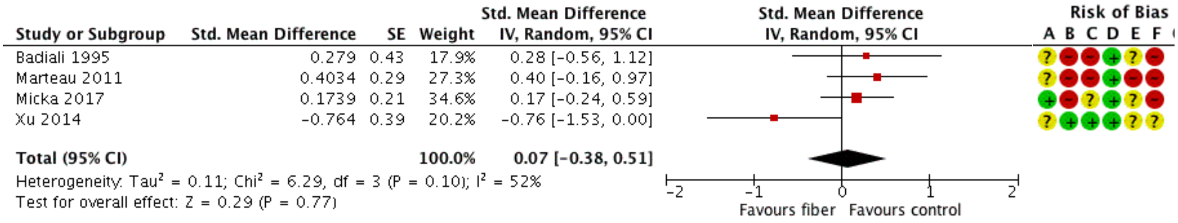

Supplementary Figure 16A Forest plot of severity of bloating in randomized controlled trials comparing fiber with control in adults with chronic constipation (n=239). Values were calculated as standardized mean difference (95% CIs) using a random-effects model. Risk of bias key: A=bias arising from the randomization process; B=bias arising from deviations from intended interventions; C=bias due to missing outcome data; D=bias in measurement of the outcome; E=bias in selection of the reported result; F=overall risk of bias. Abbreviation: IV, inverse variance.

Study or Subgroup	Std. Mean Difference	SE	Weight	Std. Mean Difference IV, Random, 95% CI
**2.13.1 Inulin-type fructans**				
Marteau 2011	0.4034	0.29	27.3%	0.40 [-0.16, 0.97]
Micka 2017	0.1739	0.21	34.6%	0.17 [-0.24, 0.59]
**Subtotal (95% CI)**			**62.0%**	**0.25 [-0.08, 0.59]**
Heterogeneity: $Tau^2 = 0.00$; $Chi^2 = 0.41$, df = 1 (P = 0.52); $I^2 = 0\%$				
Test for overall effect: Z = 1.49 (P = 0.14)				
**2.13.2 Pectin**				
Xu 2014	-0.764	0.39	20.2%	-0.76 [-1.53, 0.00]
**Subtotal (95% CI)**			**20.2%**	**-0.76 [-1.53, 0.00]**
Heterogeneity: Not applicable				
Test for overall effect: Z = 1.96 (P = 0.05)				
**2.13.3 Wheat bran**				
Badiali 1995	0.279	0.43	17.9%	0.28 [-0.56, 1.12]
**Subtotal (95% CI)**			**17.9%**	**0.28 [-0.56, 1.12]**
Heterogeneity: Not applicable				
Test for overall effect: Z = 0.65 (P = 0.52)				
**Total (95% CI)**			**100.0%**	**0.07 [-0.38, 0.51]**
Heterogeneity: $Tau^2 = 0.11$; $Chi^2 = 6.29$, df = 3 (P = 0.10); $I^2 = 52\%$				
Test for overall effect: Z = 0.29 (P = 0.77)				
Test for subgroup differences: $Chi^2 = 5.88$, df = 2 (P = 0.05), $I^2 = 66.0\%$				

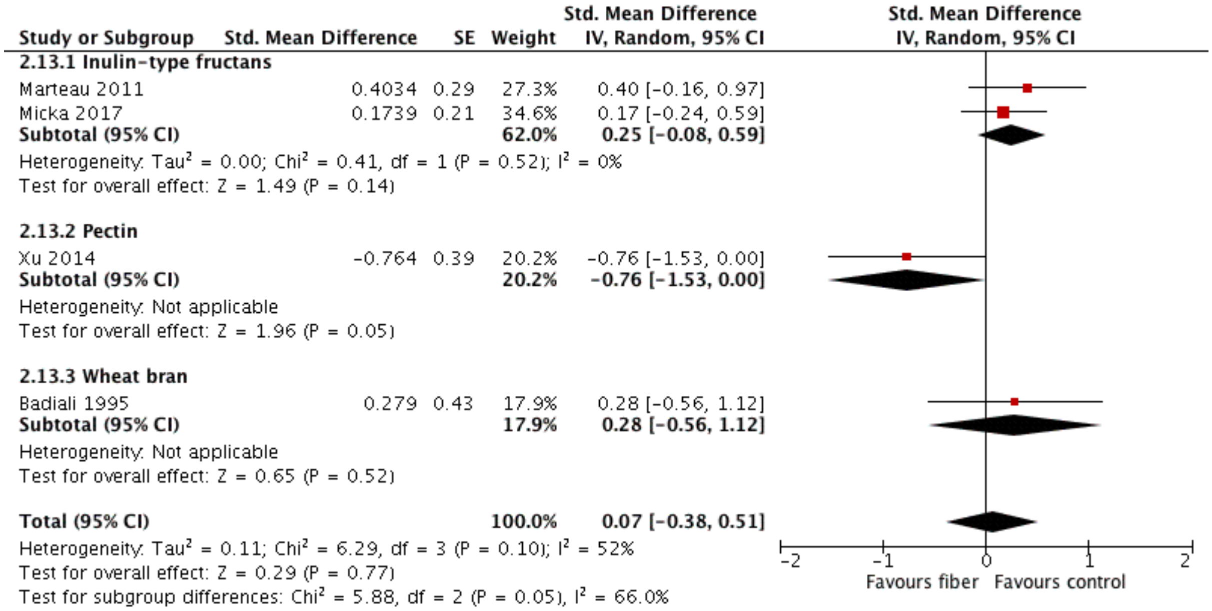

Supplementary Figure 16B Forest plot of subgroup analysis based on the type of fiber for severity of bloating in randomized controlled trials comparing fiber with control in adults with chronic constipation (n=239). Values were calculated as standardized mean difference (95% CIs) using a random-effects model. Abbreviation: IV, inverse variance.

Study or Subgroup	Std. Mean Difference	SE	Weight	Std. Mean Difference IV, Random, 95% CI
**3.12.1 Prebiotics**				
Marteau 2011	0.4034	0.29	27.3%	0.40 [-0.16, 0.97]
Micka 2017	0.1739	0.21	34.6%	0.17 [-0.24, 0.59]
**Subtotal (95% CI)**			**62.0%**	**0.25 [-0.08, 0.59]**
Heterogeneity: Tau^2 = 0.00; Chi^2 = 0.41, df = 1 (P = 0.52); I^2 = 0%				
Test for overall effect: Z = 1.49 (P = 0.14)				
**3.12.2 Non-prebiotics**				
Badiali 1995	0.279	0.43	17.9%	0.28 [-0.56, 1.12]
Xu 2014	-0.764	0.39	20.2%	-0.76 [-1.53, 0.00]
**Subtotal (95% CI)**			**38.0%**	**-0.26 [-1.28, 0.76]**
Heterogeneity: Tau^2 = 0.38; Chi^2 = 3.23, df = 1 (P = 0.07); I^2 = 69%				
Test for overall effect: Z = 0.50 (P = 0.62)				
**Total (95% CI)**			**100.0%**	**0.07 [-0.38, 0.51]**
Heterogeneity: Tau^2 = 0.11; Chi^2 = 6.29, df = 3 (P = 0.10); I^2 = 52%				
Test for overall effect: Z = 0.29 (P = 0.77)				
Test for subgroup differences: Chi^2 = 0.87, df = 1 (P = 0.35), I^2 = 0%				

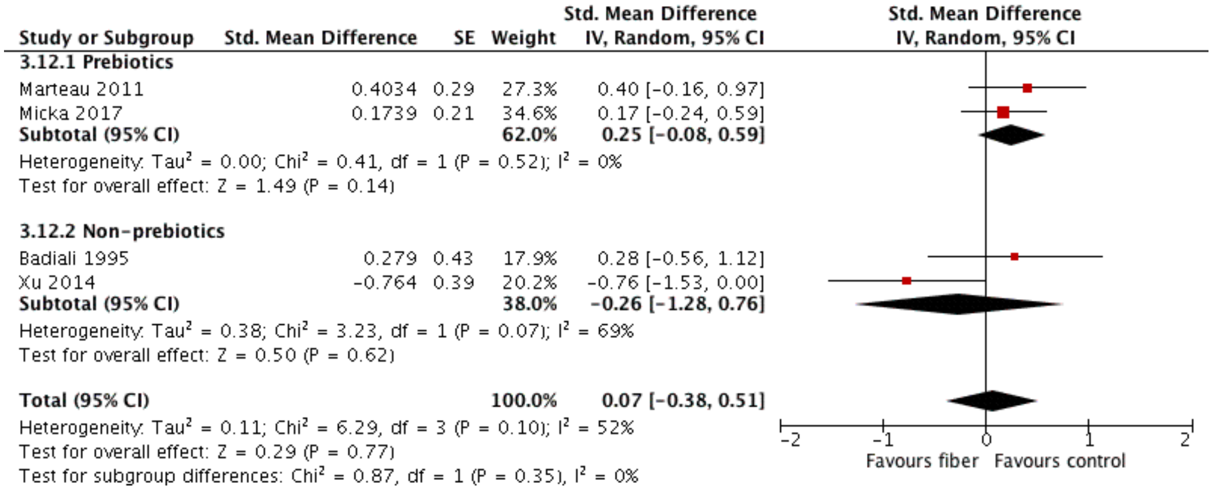

Supplementary Figure 16C Forest plot of subgroup analysis based on prebiotic status of the intervention for severity of bloating in randomized controlled trials comparing fiber with control in adults with chronic constipation (n=239). Values were calculated as standardized mean difference (95% CIs) using a random-effects model. Abbreviation: IV, inverse variance.

Severity of abdominal pain/discomfort

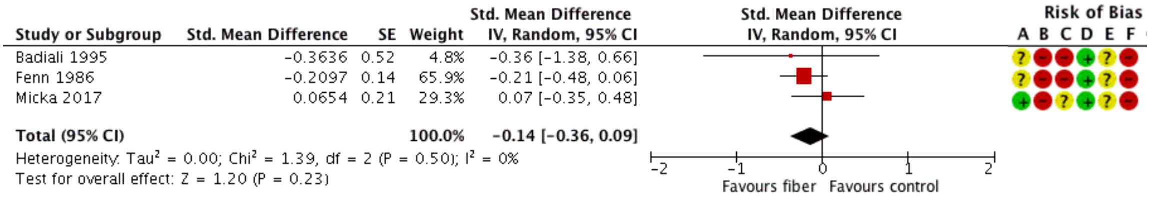

Supplementary Figure 17A Forest plot of severity of abdominal pain/discomfort in randomized controlled trials comparing fiber with control in adults with chronic constipation (n=299). Values were calculated as standardized mean difference (95% CIs) using a random-effects model. Risk of bias key: A=bias arising from the randomization process; B=bias arising from deviations from intended interventions; C=bias due to missing outcome data; D=bias in measurement of the outcome; E=bias in selection of the reported result; F=overall risk of bias. Abbreviation: IV, inverse variance.

Study or Subgroup	Std. Mean Difference	SE	Weight	Std. Mean Difference IV, Random, 95% CI
**2.15.1 Psyllium**				
Fenn 1986	-0.2097	0.14	65.9%	-0.21 [-0.48, 0.06]
**Subtotal (95% CI)**			**65.9%**	**-0.21 [-0.48, 0.06]**
Heterogeneity: Not applicable				
Test for overall effect: Z = 1.50 (P = 0.13)				
**2.15.2 Inulin-type fructans**				
Micka 2017	0.0654	0.21	29.3%	0.07 [-0.35, 0.48]
**Subtotal (95% CI)**			**29.3%**	**0.07 [-0.35, 0.48]**
Heterogeneity: Not applicable				
Test for overall effect: Z = 0.31 (P = 0.76)				
**2.15.3 Wheat bran**				
Badiali 1995	-0.3636	0.52	4.8%	-0.36 [-1.38, 0.66]
**Subtotal (95% CI)**			**4.8%**	**-0.36 [-1.38, 0.66]**
Heterogeneity: Not applicable				
Test for overall effect: Z = 0.70 (P = 0.48)				
**Total (95% CI)**			**100.0%**	**-0.14 [-0.36, 0.09]**
Heterogeneity: $Tau^2 = 0.00$; $Chi^2 = 1.39$, df = 2 (P = 0.50); $I^2 = 0\%$				
Test for overall effect: Z = 1.20 (P = 0.23)				
Test for subgroup differences: $Chi^2 = 1.39$, df = 2 (P = 0.50), $I^2 = 0\%$				

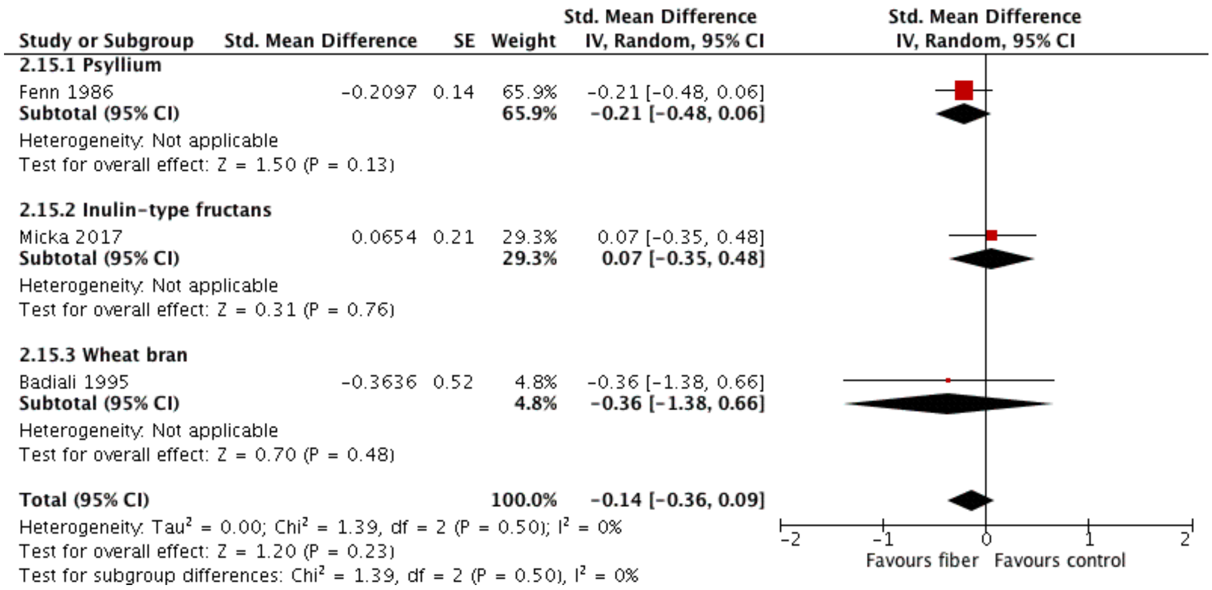

Supplementary Figure 17B Forest plot of subgroup analysis based on type of fiber for severity of abdominal pain/discomfort in randomized controlled trials comparing fiber with control in adults with chronic constipation (n=299). Values were calculated as standardized mean difference (95% CIs) using a random-effects model. Abbreviation: IV, inverse variance.

Study or Subgroup	Std. Mean Difference	SE	Weight	Std. Mean Difference IV, Random, 95% CI
**3.13.1 Prebiotics**				
Micka 2017	0.0654	0.21	29.3%	0.07 [−0.35, 0.48]
**Subtotal (95% CI)**			**29.3%**	**0.07 [−0.35, 0.48]**
Heterogeneity: Not applicable				
Test for overall effect: Z = 0.31 (P = 0.76)				
**3.13.2 Non-prebiotics**				
Badiali 1995	−0.3636	0.52	4.8%	−0.36 [−1.38, 0.66]
Fenn 1986	−0.2097	0.14	65.9%	−0.21 [−0.48, 0.06]
**Subtotal (95% CI)**			**70.7%**	**−0.22 [−0.49, 0.04]**
Heterogeneity: $Tau^2 = 0.00$; $Chi^2 = 0.08$, df = 1 (P = 0.78); $I^2 = 0\%$				
Test for overall effect: Z = 1.63 (P = 0.10)				
**Total (95% CI)**			**100.0%**	**−0.14 [−0.36, 0.09]**
Heterogeneity: $Tau^2 = 0.00$; $Chi^2 = 1.39$, df = 2 (P = 0.50); $I^2 = 0\%$				
Test for overall effect: Z = 1.20 (P = 0.23)				
Test for subgroup differences: $Chi^2 = 1.31$, df = 1 (P = 0.25), $I^2 = 23.5\%$				

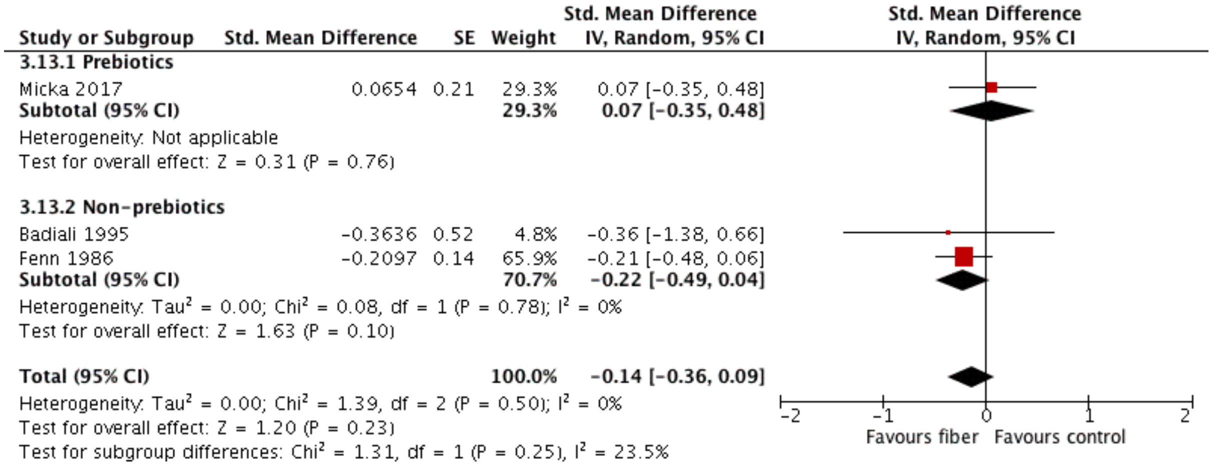

Supplementary Figure 17C Forest plot of subgroup analysis based on prebiotic status of the intervention for severity of abdominal pain/discomfort in randomized controlled trials comparing fiber with control in adults with chronic constipation (n=299). Values were calculated as standardized mean difference (95% CIs) using a random-effects model. Abbreviation: IV, inverse variance.

Study or Subgroup	Std. Mean Difference	SE	Weight	Std. Mean Difference IV, Random, 95% CI
**4.10.1 Duration of treatment < 4 weeks**				
Fenn 1986	−0.2097	0.14	65.9%	−0.21 [−0.48, 0.06]
**Subtotal (95% CI)**			**65.9%**	**−0.21 [−0.48, 0.06]**
Heterogeneity: Not applicable				
Test for overall effect: Z = 1.50 (P = 0.13)				
**4.10.2 Duration of treatment ≥ 4 weeks**				
Badiali 1995	−0.3636	0.52	4.8%	−0.36 [−1.38, 0.66]
Micka 2017	0.0654	0.21	29.3%	0.07 [−0.35, 0.48]
**Subtotal (95% CI)**			**34.1%**	**0.01 [−0.38, 0.39]**
Heterogeneity: Tau² = 0.00; Chi² = 0.59, df = 1 (P = 0.44); I² = 0%				
Test for overall effect: Z = 0.03 (P = 0.98)				
**Total (95% CI)**			**100.0%**	**−0.14 [−0.36, 0.09]**
Heterogeneity: Tau² = 0.00; Chi² = 1.39, df = 2 (P = 0.50); I² = 0%				
Test for overall effect: Z = 1.20 (P = 0.23)				
Test for subgroup differences: Chi² = 0.80, df = 1 (P = 0.37), I² = 0%				

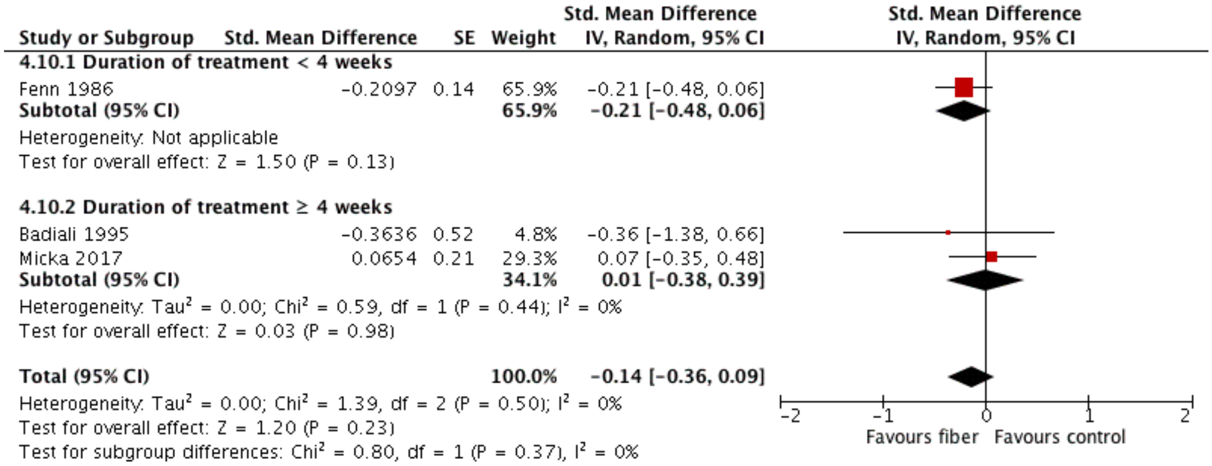

Supplementary Figure 17D Forest plot of subgroup analysis based on duration of treatment for severity of abdominal pain/discomfort in randomized controlled trials comparing fiber with control in adults with chronic constipation (n=299). Values were calculated as standardized mean difference (95% CIs) using a random-effects model. Abbreviation: IV, inverse variance.

Patient Assessment of Constipation Quality of Life (PAC-QoL) global and subscales (satisfaction, physical discomfort, worries and concerns, psychosocial discomfort)

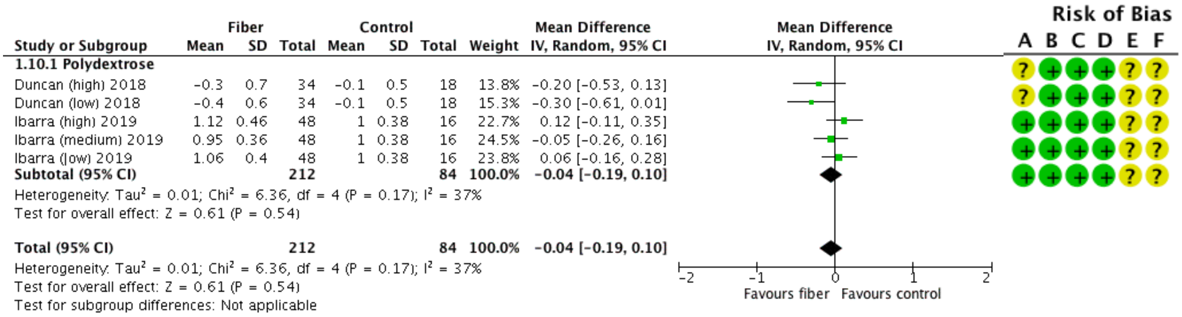

Supplementary Figure 18A Forest plot of PAC-QoL (global) in randomized controlled trials comparing fiber with control in adults with chronic constipation (n=296). Values were calculated as mean difference (95% CIs) using a random-effects model. Risk of bias key: A=bias arising from the randomization process; B=bias arising from deviations from intended interventions; C=bias due to missing outcome data; D=bias in measurement of the outcome; E=bias in selection of the reported result; F=overall risk of bias. Abbreviations: IV, inverse variance; PAC-QoL, Patient Assessment of Constipation Quality of Life.

Study or Subgroup	Fiber Mean	Fiber SD	Fiber Total	Control Mean	Control SD	Control Total	Weight	Mean Difference IV, Random, 95% CI
**5.14.1 Low dose (≤10 g/d)**								
Duncan (low) 2018	−0.4	0.6	34	−0.1	0.5	18	15.3%	−0.30 [−0.61, 0.01]
Ibarra (medium) 2019	0.95	0.36	48	1	0.38	16	24.5%	−0.05 [−0.26, 0.16]
Ibarra (low) 2019	1.06	0.4	48	1	0.38	16	23.8%	0.06 [−0.16, 0.28]
**Subtotal (95% CI)**			**130**			**50**	**63.6%**	**−0.07 [−0.26, 0.11]**
Heterogeneity: Tau² = 0.01; Chi² = 3.52, df = 2 (P = 0.17); I² = 43%								
Test for overall effect: Z = 0.75 (P = 0.45)								
**5.14.2 High dose (>10 g/d)**								
Duncan (high) 2018	−0.3	0.7	34	−0.1	0.5	18	13.8%	−0.20 [−0.53, 0.13]
Ibarra (high) 2019	1.12	0.46	48	1	0.38	16	22.7%	0.12 [−0.11, 0.35]
**Subtotal (95% CI)**			**82**			**34**	**36.4%**	**−0.02 [−0.33, 0.29]**
Heterogeneity: Tau² = 0.03; Chi² = 2.45, df = 1 (P = 0.12); I² = 59%								
Test for overall effect: Z = 0.11 (P = 0.92)								
**Total (95% CI)**			**212**			**84**	**100.0%**	**−0.04 [−0.19, 0.10]**
Heterogeneity: Tau² = 0.01; Chi² = 6.36, df = 4 (P = 0.17); I² = 37%								
Test for overall effect: Z = 0.61 (P = 0.54)								
Test for subgroup differences: Chi² = 0.09, df = 1 (P = 0.77), I² = 0%								

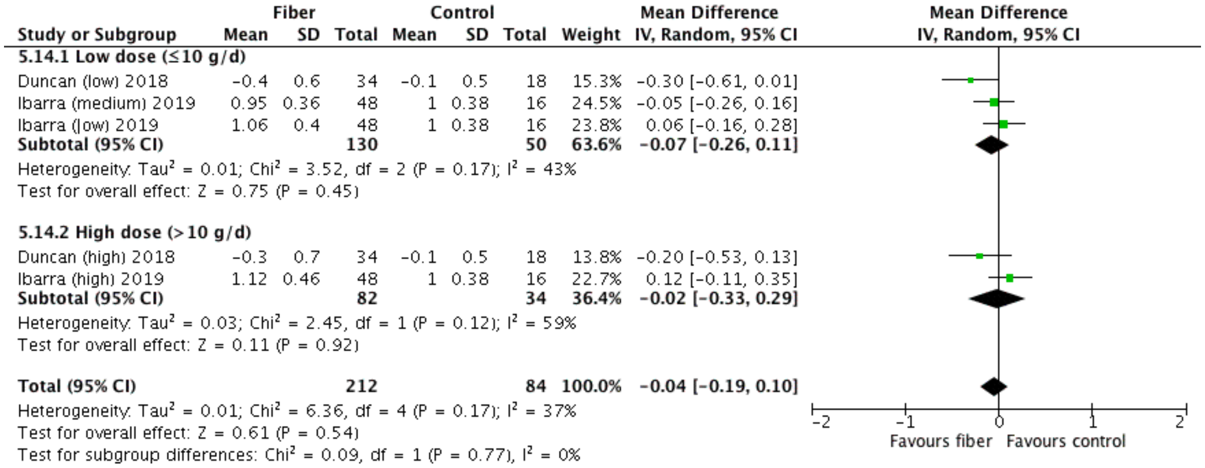

Supplementary Figure 18B Forest plot of subgroup analysis based on dose administered for PAC-QoL (global) in randomized controlled trials comparing fiber with control in adults with chronic constipation (n=296). Values were calculated as mean difference (95% CIs) using a random-effects model. Abbreviations: IV, inverse variance; PAC-QoL, Patient Assessment of Constipation Quality of Life.

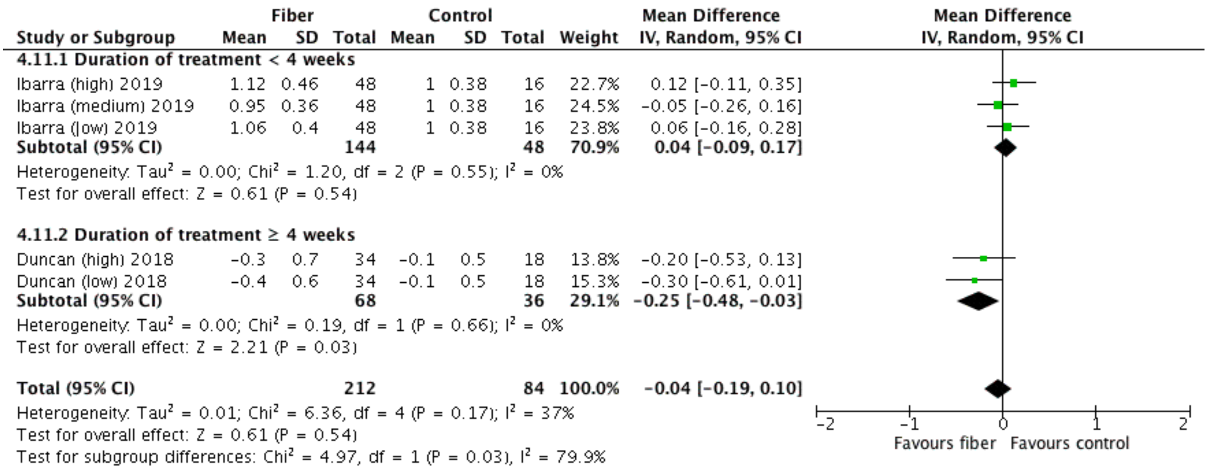

Supplementary Figure 18C Forest plot of subgroup analysis based on treatment duration for PAC-QoL (global) in randomized controlled trials comparing fiber with control in adults with chronic constipation (n=296). Values were calculated as mean difference (95% CIs) using a random-effects model. Abbreviations: IV, inverse variance; PAC-QoL, Patient Assessment of Constipation Quality of Life.

Study or Subgroup	Fiber Mean	Fiber SD	Fiber Total	Control Mean	Control SD	Control Total	Weight	Mean Difference IV, Random, 95% CI	A	B	C	D	E	F
Duncan (high) 2018	−0.3	1.2	34	−0.1	0.9	18	12.6%	−0.20 [−0.78, 0.38]	?	+	+	+	?	?
Duncan (low) 2018	−0.6	0.9	34	−0.1	0.9	18	14.4%	−0.50 [−1.01, 0.01]	?	+	+	+	?	?
Ibarra (high) 2019	1.88	0.75	48	1.56	0.58	16	19.6%	0.32 [−0.03, 0.67]	+	+	+	+	?	?
Ibarra (medium) 2019	1.85	0.73	48	1.56	0.58	16	19.7%	0.29 [−0.06, 0.64]	+	+	+	+	?	?
Ibarra (low) 2019	1.49	0.72	48	1.56	0.58	16	19.8%	−0.07 [−0.42, 0.28]	+	+	+	+	?	?
Micka 2017	1.53	1.15	44	1.93	1.38	44	13.9%	−0.40 [−0.93, 0.13]	+	−	?	+	?	−
**Total (95% CI)**			**256**			**128**	**100.0%**	**−0.05 [−0.32, 0.23]**						

Heterogeneity: $Tau^2 = 0.07$; $Chi^2 = 12.15$, $df = 5$ ($P = 0.03$); $I^2 = 59\%$

Test for overall effect: $Z = 0.34$ ($P = 0.74$)

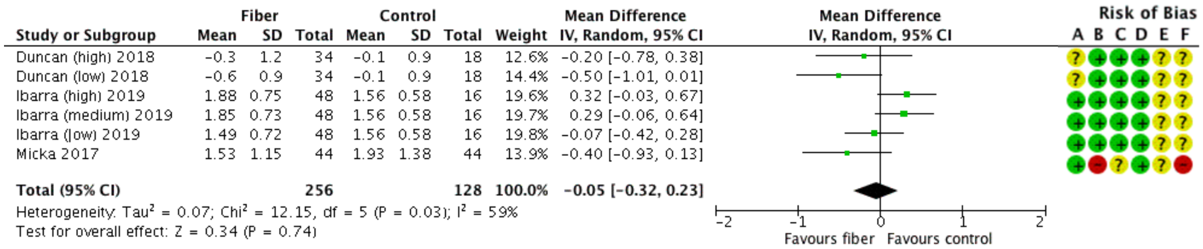

Supplementary Figure 19A Forest plot of PAC-QoL (satisfaction) in randomized controlled trials comparing fiber with control in adults with chronic constipation (n=384). Values were calculated as mean difference (95% CIs) using a random-effects model. Risk of bias key: A=bias arising from the randomization process; B=bias arising from deviations from intended interventions; C=bias due to missing outcome data; D=bias in measurement of the outcome; E=bias in selection of the reported result; F=overall risk of bias. Abbreviations: IV, inverse variance; PAC-QoL, Patient Assessment of Constipation Quality of Life.

Study or Subgroup	Fiber Mean	Fiber SD	Fiber Total	Control Mean	Control SD	Control Total	Weight	Mean Difference IV, Random, 95% CI
**2.17.1 Polydextrose**								
Duncan (high) 2018	−0.3	1.2	34	−0.1	0.9	18	12.6%	−0.20 [−0.78, 0.38]
Duncan (low) 2018	−0.6	0.9	34	−0.1	0.9	18	14.4%	−0.50 [−1.01, 0.01]
Ibarra (high) 2019	1.88	0.75	48	1.56	0.58	16	19.6%	0.32 [−0.03, 0.67]
Ibarra (medium) 2019	1.85	0.73	48	1.56	0.58	16	19.7%	0.29 [−0.06, 0.64]
Ibarra (low) 2019	1.49	0.72	48	1.56	0.58	16	19.8%	−0.07 [−0.42, 0.28]
**Subtotal (95% CI)**			**212**			**84**	**86.1%**	**0.01 [−0.27, 0.30]**
Heterogeneity: Tau² = 0.06; Chi² = 9.57, df = 4 (P = 0.05); I² = 58%								
Test for overall effect: Z = 0.09 (P = 0.93)								
**2.17.2 Inulin-type fructans**								
Micka 2017	1.53	1.15	44	1.93	1.38	44	13.9%	−0.40 [−0.93, 0.13]
**Subtotal (95% CI)**			**44**			**44**	**13.9%**	**−0.40 [−0.93, 0.13]**
Heterogeneity: Not applicable								
Test for overall effect: Z = 1.48 (P = 0.14)								
**Total (95% CI)**			**256**			**128**	**100.0%**	**−0.05 [−0.32, 0.23]**
Heterogeneity: Tau² = 0.07; Chi² = 12.15, df = 5 (P = 0.03); I² = 59%								
Test for overall effect: Z = 0.34 (P = 0.74)								
Test for subgroup differences: Chi² = 1.80, df = 1 (P = 0.18), I² = 44.3%								

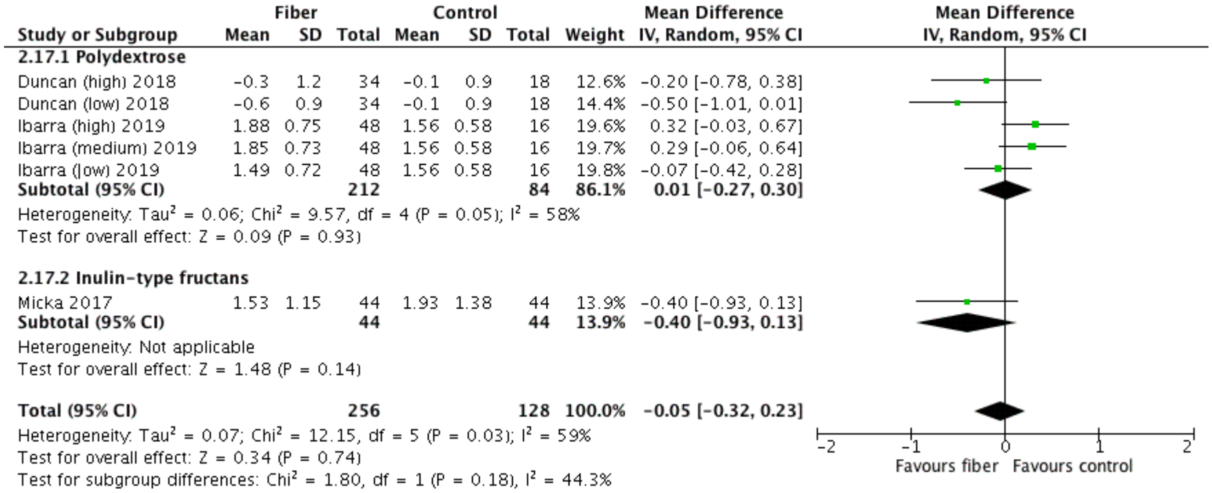

Supplementary Figure 19B Forest plot of subgroup analysis based on type of fiber for PAC-QoL (satisfaction) in randomized controlled trials comparing fiber with control in adults with chronic constipation (n=384). Values were calculated as standardized mean difference (95% CIs) using a random-effects model. Abbreviations: IV, inverse variance; PAC-QoL, Patient Assessment of Constipation Quality of Life.

Study or Subgroup	Fiber Mean	Fiber SD	Fiber Total	Control Mean	Control SD	Control Total	Weight	Mean Difference IV, Random, 95% CI
**5.15.1 Low dose (≤10 g/d)**								
Duncan (low) 2018	−0.6	0.9	34	−0.1	0.9	18	14.4%	−0.50 [−1.01, 0.01]
Ibarra (medium) 2019	1.85	0.73	48	1.56	0.58	16	19.7%	0.29 [−0.06, 0.64]
Ibarra (low) 2019	1.49	0.72	48	1.56	0.58	16	19.8%	−0.07 [−0.42, 0.28]
**Subtotal (95% CI)**			**130**			**50**	**53.9%**	**−0.06 [−0.47, 0.35]**
Heterogeneity: Tau² = 0.09; Chi² = 6.40, df = 2 (P = 0.04); I² = 69%								
Test for overall effect: Z = 0.28 (P = 0.78)								
**5.15.2 High dose (>10 g/d)**								
Duncan (high) 2018	−0.3	1.2	34	−0.1	0.9	18	12.6%	−0.20 [−0.78, 0.38]
Ibarra (high) 2019	1.88	0.75	48	1.56	0.58	16	19.6%	0.32 [−0.03, 0.67]
Micka 2017	1.53	1.15	44	1.93	1.38	44	13.9%	−0.40 [−0.93, 0.13]
**Subtotal (95% CI)**			**126**			**78**	**46.1%**	**−0.05 [−0.52, 0.42]**
Heterogeneity: Tau² = 0.11; Chi² = 5.69, df = 2 (P = 0.06); I² = 65%								
Test for overall effect: Z = 0.23 (P = 0.82)								
**Total (95% CI)**			**256**			**128**	**100.0%**	**−0.05 [−0.32, 0.23]**
Heterogeneity: Tau² = 0.07; Chi² = 12.15, df = 5 (P = 0.03); I² = 59%								
Test for overall effect: Z = 0.34 (P = 0.74)								
Test for subgroup differences: Chi² = 0.00, df = 1 (P = 0.99), I² = 0%								

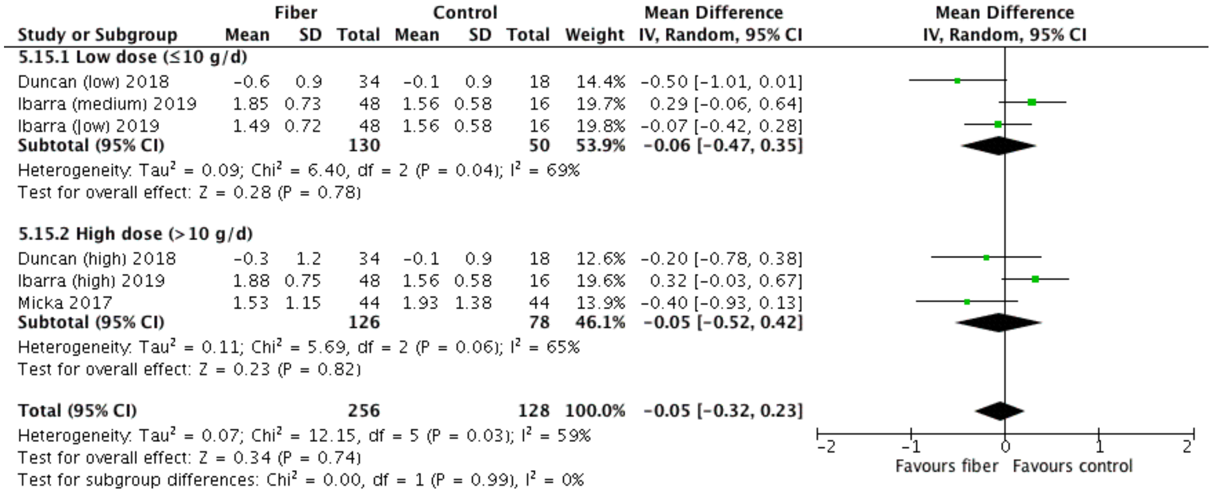

Supplementary Figure 19C Forest plot of subgroup analysis based on dose administered for PAC-QoL (satisfaction) in randomized controlled trials comparing fiber with control in adults with chronic constipation (n=384). Values were calculated as mean difference (95% CIs) using a random-effects model. Abbreviations: IV, inverse variance; PAC-QoL, Patient Assessment of Constipation Quality of Life.

Study or Subgroup	Fiber Mean	Fiber SD	Fiber Total	Control Mean	Control SD	Control Total	Weight	Mean Difference IV, Random, 95% CI
**4.12.1 Duration of treatment < 4 weeks**								
Ibarra (high) 2019	1.88	0.75	48	1.56	0.58	16	19.6%	0.32 [−0.03, 0.67]
Ibarra (medium) 2019	1.85	0.73	48	1.56	0.58	16	19.7%	0.29 [−0.06, 0.64]
Ibarra (low) 2019	1.49	0.72	48	1.56	0.58	16	19.8%	−0.07 [−0.42, 0.28]
**Subtotal (95% CI)**			**144**			**48**	**59.1%**	**0.18 [−0.07, 0.43]**
Heterogeneity: $Tau^2 = 0.02$; $Chi^2 = 2.94$, df = 2 (P = 0.23); $I^2 = 32\%$								
Test for overall effect: Z = 1.42 (P = 0.15)								
**4.12.2 Duration of treatment ≥ 4 weeks**								
Duncan (high) 2018	−0.3	1.2	34	−0.1	0.9	18	12.6%	−0.20 [−0.78, 0.38]
Duncan (low) 2018	−0.6	0.9	34	−0.1	0.9	18	14.4%	−0.50 [−1.01, 0.01]
Micka 2017	1.53	1.15	44	1.93	1.38	44	13.9%	−0.40 [−0.93, 0.13]
**Subtotal (95% CI)**			**112**			**80**	**40.9%**	**−0.38 [−0.69, −0.07]**
Heterogeneity: $Tau^2 = 0.00$; $Chi^2 = 0.59$, df = 2 (P = 0.75); $I^2 = 0\%$								
Test for overall effect: Z = 2.38 (P = 0.02)								
**Total (95% CI)**			**256**			**128**	**100.0%**	**−0.05 [−0.32, 0.23]**
Heterogeneity: $Tau^2 = 0.07$; $Chi^2 = 12.15$, df = 5 (P = 0.03); $I^2 = 59\%$								
Test for overall effect: Z = 0.34 (P = 0.74)								
Test for subgroup differences: $Chi^2 = 7.58$, df = 1 (P = 0.006), $I^2 = 86.8\%$								

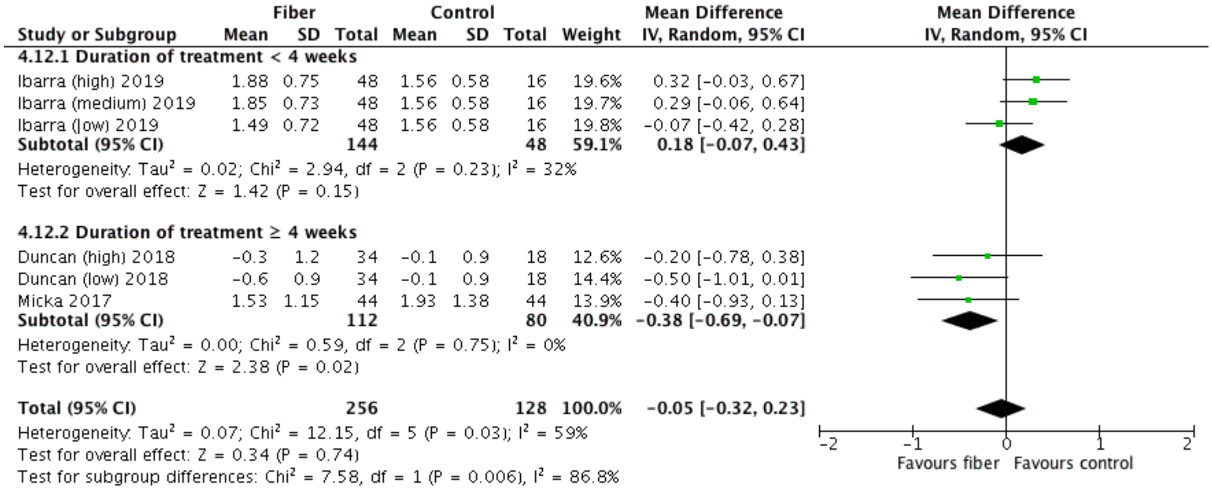

Supplementary Figure 19D Forest plot of subgroup analysis based on treatment duration for PAC-QoL (satisfaction) in randomized controlled trials comparing fiber with control in adults with chronic constipation (n=384). Values were calculated as mean difference (95% CIs) using a random-effects model. Abbreviations: IV, inverse variance; PAC-QoL, Patient Assessment of Constipation Quality of Life.

Study or Subgroup	Fiber Mean	Fiber SD	Fiber Total	Control Mean	Control SD	Control Total	Weight	Mean Difference IV, Random, 95% CI	Risk of Bias A	B	C	D	E	F
Duncan (high) 2018	1.7	0.95	34	1.2	0.69	18	15.3%	0.50 [0.05, 0.95]	?	+	+	+	?	?
Duncan (low) 2018	1.5	0.9	34	1.2	0.69	18	15.9%	0.30 [−0.14, 0.74]	?	+	+	+	?	?
Ibarra (high) 2019	1.18	0.87	48	1.05	0.73	16	16.2%	0.13 [−0.30, 0.56]	+	+	+	+	?	?
Ibarra (medium) 2019	0.92	0.76	48	1.05	0.73	16	17.2%	−0.13 [−0.55, 0.29]	+	+	+	+	?	?
Ibarra (low) 2019	1.2	0.76	48	1.05	0.73	16	17.2%	0.15 [−0.27, 0.57]	+	+	+	+	?	?
Micka 2017	1.2	1	44	1.37	0.92	44	18.2%	−0.17 [−0.57, 0.23]	+	−	?	+	?	−
**Total (95% CI)**			**256**			**128**	**100.0%**	**0.12 [−0.08, 0.32]**						

Heterogeneity: $Tau^2 = 0.02$; $Chi^2 = 6.77$, $df = 5$ ($P = 0.24$); $I^2 = 26\%$
Test for overall effect: $Z = 1.14$ ($P = 0.25$)

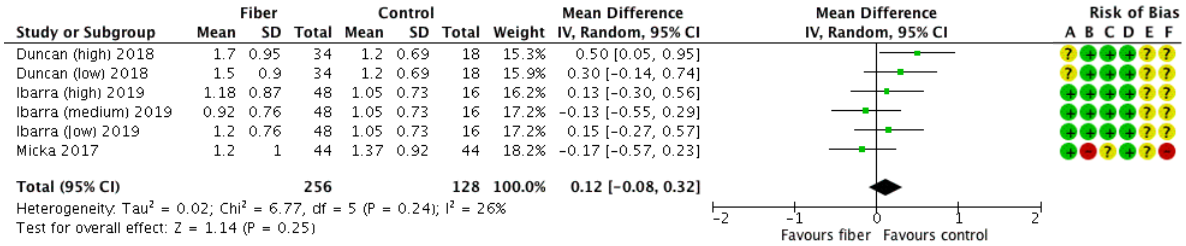

Supplementary Figure 20A Forest plot of PAC-QoL (physical discomfort) in randomized controlled trials comparing fiber with control in adults with chronic constipation (n=384). Values were calculated as mean difference (95% CIs) using a random-effects model. Risk of bias key: A=bias arising from the randomization process; B=bias arising from deviations from intended interventions; C = bias due to missing outcome data; D=bias in measurement of the outcome; E = bias in selection of the reported result; F= overall risk of bias. Abbreviations: IV, inverse variance; PAC-QoL, Patient Assessment of Constipation Quality of Life.

Study or Subgroup	Fiber Mean	Fiber SD	Fiber Total	Control Mean	Control SD	Control Total	Weight	Mean Difference IV, Random, 95% CI
**2.18.1 Polydextrose**								
Duncan (high) 2018	1.7	0.95	34	1.2	0.69	18	15.3%	0.50 [0.05, 0.95]
Duncan (low) 2018	1.5	0.9	34	1.2	0.69	18	15.9%	0.30 [−0.14, 0.74]
Ibarra (high) 2019	1.18	0.87	48	1.05	0.73	16	16.2%	0.13 [−0.30, 0.56]
Ibarra (medium) 2019	0.92	0.76	48	1.05	0.73	16	17.2%	−0.13 [−0.55, 0.29]
Ibarra (low) 2019	1.2	0.76	48	1.05	0.73	16	17.2%	0.15 [−0.27, 0.57]
**Subtotal (95% CI)**			**212**			**84**	**81.8%**	**0.18 [−0.02, 0.38]**
Heterogeneity: $Tau^2 = 0.00$; $Chi^2 = 4.41$, df = 4 (P = 0.35); $I^2 = 9\%$								
Test for overall effect: Z = 1.74 (P = 0.08)								
**2.18.2 Inulin type fructans**								
Micka 2017	1.2	1	44	1.37	0.92	44	18.2%	−0.17 [−0.57, 0.23]
**Subtotal (95% CI)**			**44**			**44**	**18.2%**	**−0.17 [−0.57, 0.23]**
Heterogeneity: Not applicable								
Test for overall effect: Z = 0.83 (P = 0.41)								
**Total (95% CI)**			**256**			**128**	**100.0%**	**0.12 [−0.08, 0.32]**
Heterogeneity: $Tau^2 = 0.02$; $Chi^2 = 6.77$, df = 5 (P = 0.24); $I^2 = 26\%$								
Test for overall effect: Z = 1.14 (P = 0.25)								
Test for subgroup differences: $Chi^2 = 2.33$, df = 1 (P = 0.13), $I^2 = 57.1\%$								

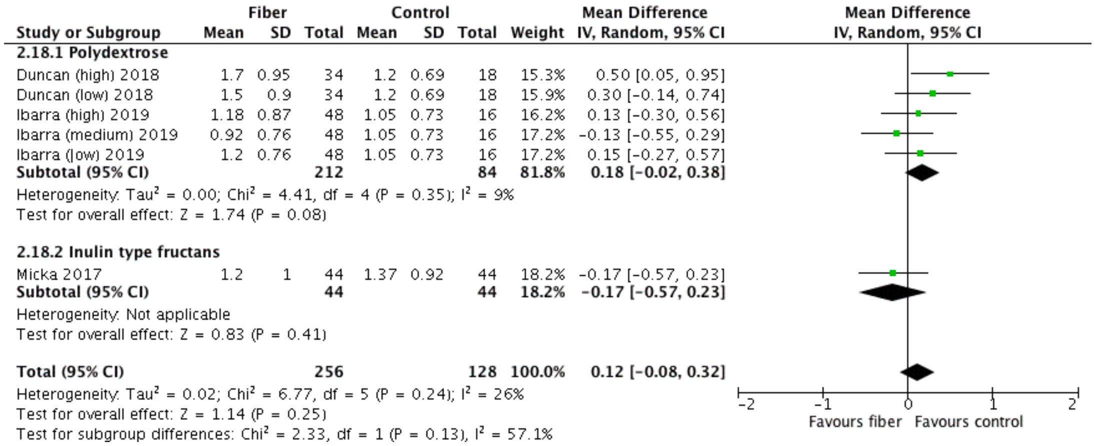

Supplementary Figure 20B Forest plot of subgroup analysis based on type of fiber for PAC-QoL (physical discomfort) in randomized controlled trials comparing fiber with control in adults with chronic constipation (n=384). Values were calculated as standardized mean difference (95% CIs) using a random-effects model. Abbreviations: IV, inverse variance; PAC-QoL, Patient Assessment of Constipation Quality of Life.

Study or Subgroup	Fiber Mean	Fiber SD	Fiber Total	Control Mean	Control SD	Control Total	Weight	Mean Difference IV, Random, 95% CI
**5.16.1 Low dose (≤10 g/d)**								
Duncan (low) 2018	1.5	0.9	34	1.2	0.69	18	15.9%	0.30 [−0.14, 0.74]
Ibarra (medium) 2019	0.92	0.76	48	1.05	0.73	16	17.2%	−0.13 [−0.55, 0.29]
Ibarra (low) 2019	1.2	0.76	48	1.05	0.73	16	17.2%	0.15 [−0.27, 0.57]
**Subtotal (95% CI)**			**130**			**50**	**50.3%**	**0.10 [−0.15, 0.35]**
Heterogeneity: $Tau^2 = 0.00$; $Chi^2 = 2.02$, df = 2 (P = 0.36); $I^2 = 1\%$								
Test for overall effect: Z = 0.80 (P = 0.42)								
**5.16.2 High dose (>10 g/d)**								
Duncan (high) 2018	1.7	0.95	34	1.2	0.69	18	15.3%	0.50 [0.05, 0.95]
Ibarra (high) 2019	1.18	0.87	48	1.05	0.73	16	16.2%	0.13 [−0.30, 0.56]
Micka 2017	1.2	1	44	1.37	0.92	44	18.2%	−0.17 [−0.57, 0.23]
**Subtotal (95% CI)**			**126**			**78**	**49.7%**	**0.14 [−0.24, 0.52]**
Heterogeneity: $Tau^2 = 0.07$; $Chi^2 = 4.73$, df = 2 (P = 0.09); $I^2 = 58\%$								
Test for overall effect: Z = 0.73 (P = 0.46)								
**Total (95% CI)**			**256**			**128**	**100.0%**	**0.12 [−0.08, 0.32]**
Heterogeneity: $Tau^2 = 0.02$; $Chi^2 = 6.77$, df = 5 (P = 0.24); $I^2 = 26\%$								
Test for overall effect: Z = 1.14 (P = 0.25)								
Test for subgroup differences: $Chi^2 = 0.03$, df = 1 (P = 0.85), $I^2 = 0\%$								

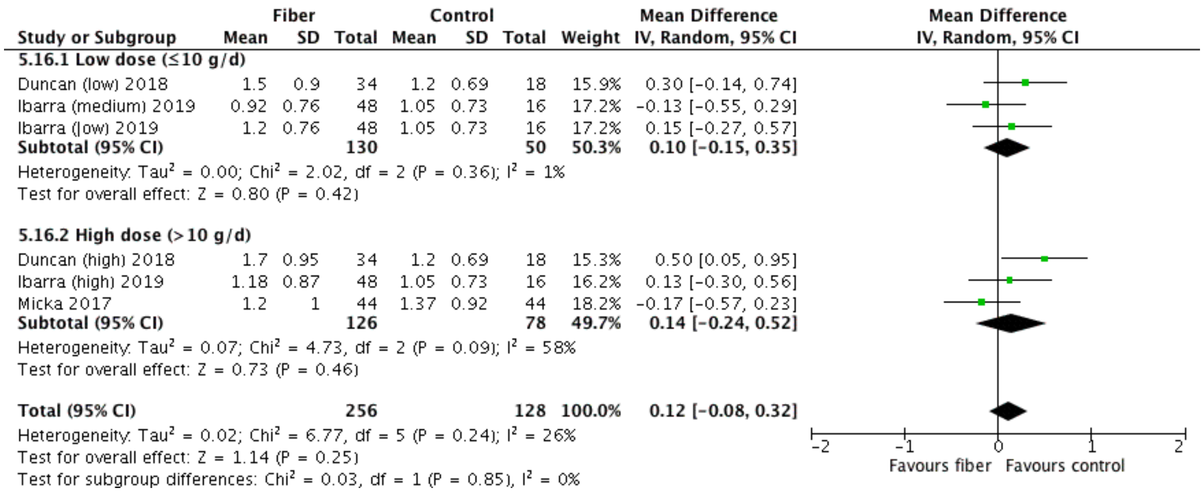

Supplementary Figure 20C Forest plot of subgroup analysis based on dose administered for PAC-QoL (physical discomfort) in randomized controlled trials comparing fiber with control in adults with chronic constipation (n=384). Values were calculated as mean difference (95% CIs) using a random-effects model. Abbreviations: IV, inverse variance; PAC-QoL, Patient Assessment of Constipation Quality of Life.

Study or Subgroup	Fiber Mean	Fiber SD	Fiber Total	Control Mean	Control SD	Control Total	Weight	Mean Difference IV, Random, 95% CI
**4.13.1 Duration of treatment < 4 weeks**								
Ibarra (high) 2019	1.18	0.87	48	1.05	0.73	16	16.2%	0.13 [−0.30, 0.56]
Ibarra (medium) 2019	0.92	0.76	48	1.05	0.73	16	17.2%	−0.13 [−0.55, 0.29]
Ibarra (low) 2019	1.2	0.76	48	1.05	0.73	16	17.2%	0.15 [−0.27, 0.57]
**Subtotal (95% CI)**			**144**			**48**	**50.6%**	**0.05 [−0.20, 0.29]**
Heterogeneity: $Tau^2 = 0.00$; $Chi^2 = 1.07$, df = 2 (P = 0.59); $I^2 = 0\%$								
Test for overall effect: Z = 0.38 (P = 0.70)								
**4.13.2 Duration of treatment ≥ 4 weeks**								
Duncan (high) 2018	1.7	0.95	34	1.2	0.69	18	15.3%	0.50 [0.05, 0.95]
Duncan (low) 2018	1.5	0.9	34	1.2	0.69	18	15.9%	0.30 [−0.14, 0.74]
Micka 2017	1.2	1	44	1.37	0.92	44	18.2%	−0.17 [−0.57, 0.23]
**Subtotal (95% CI)**			**112**			**80**	**49.4%**	**0.20 [−0.20, 0.60]**
Heterogeneity: $Tau^2 = 0.08$; $Chi^2 = 5.14$, df = 2 (P = 0.08); $I^2 = 61\%$								
Test for overall effect: Z = 0.98 (P = 0.33)								
**Total (95% CI)**			**256**			**128**	**100.0%**	**0.12 [−0.08, 0.32]**
Heterogeneity: $Tau^2 = 0.02$; $Chi^2 = 6.77$, df = 5 (P = 0.24); $I^2 = 26\%$								
Test for overall effect: Z = 1.14 (P = 0.25)								
Test for subgroup differences: $Chi^2 = 0.40$, df = 1 (P = 0.53), $I^2 = 0\%$								

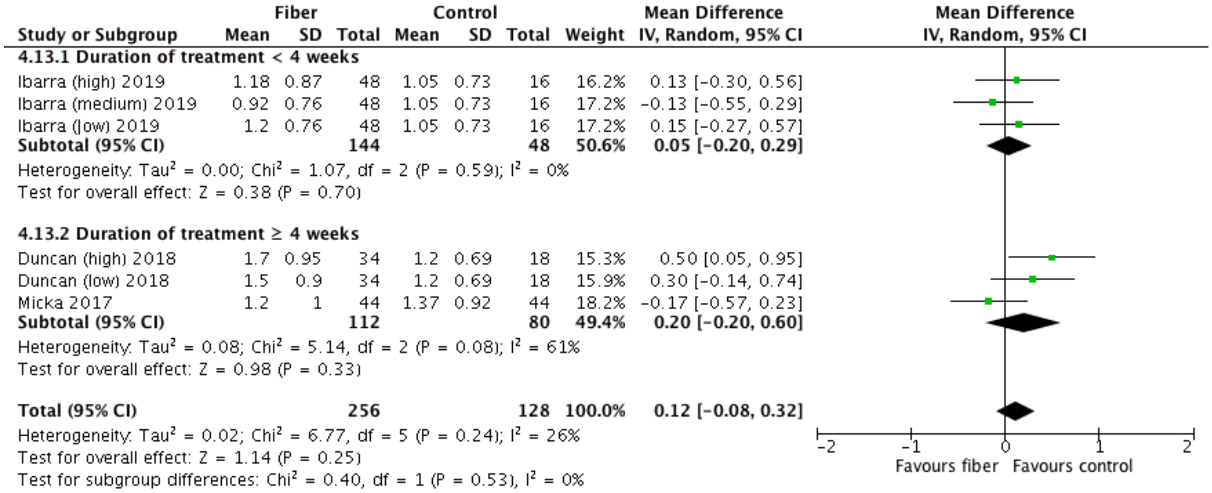

Supplementary Figure 20D Forest plot of subgroup analysis based on treatment duration for PAC-QoL (physical discomfort) in randomized controlled trials comparing fiber with control in adults with chronic constipation (n=384). Values were calculated as mean difference (95% CIs) using a random-effects model. Abbreviations: IV, inverse variance; PAC-QoL, Patient Assessment of Constipation Quality of Life.

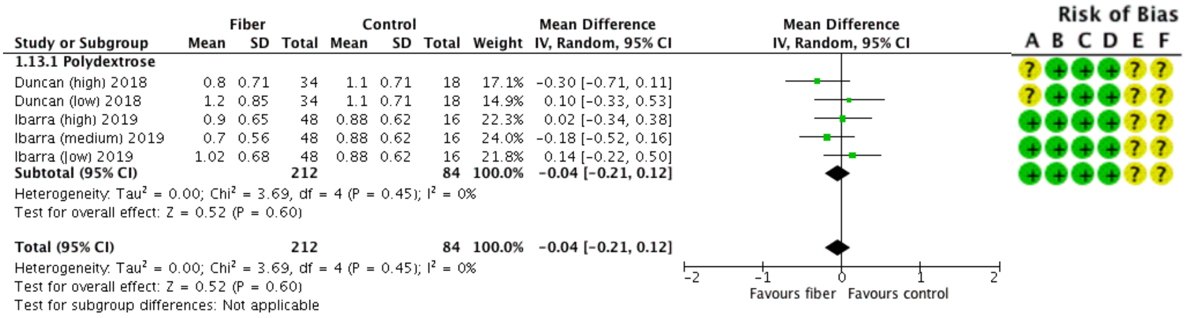

Supplementary Figure 21A Forest plot of PAC-QoL (worries and concerns) in randomized controlled trials comparing fiber with control in adults with chronic constipation (n=296). Values were calculated as mean difference (95% CIs) using a random-effects model. Risk of bias key: A=bias arising from the randomization process; B=bias arising from deviations from intended interventions; C=bias due to missing outcome data; D=bias in measurement of the outcome; E=bias in selection of the reported result; F=overall risk of bias. Abbreviations: IV, inverse variance; PAC-QoL, Patient Assessment of Constipation Quality of Life.

Study or Subgroup	Fiber Mean	Fiber SD	Fiber Total	Control Mean	Control SD	Control Total	Weight	Mean Difference IV, Random, 95% CI
**5.17.1 Low dose (≤10 g/d)**								
Duncan (low) 2018	1.2	0.85	34	1.1	0.71	18	18.6%	0.10 [−0.33, 0.53]
Ibarra (medium) 2019	0.7	0.56	48	0.88	0.62	16	20.9%	−0.18 [−0.52, 0.16]
Ibarra (low) 2019	0	0.68	48	0.88	0.62	16	20.5%	−0.88 [−1.24, −0.52]
**Subtotal (95% CI)**			**130**			**50**	**60.0%**	**−0.33 [−0.89, 0.24]**
Heterogeneity: $Tau^2 = 0.21$; $Chi^2 = 13.39$, df = 2 (P = 0.001); $I^2 = 85\%$								
Test for overall effect: Z = 1.14 (P = 0.25)								
**5.17.2 High dose (>10 g/d)**								
Duncan (high) 2018	0.8	0.71	34	1.1	0.71	18	19.3%	−0.30 [−0.71, 0.11]
Ibarra (high) 2019	0.9	0.65	48	0.88	0.62	16	20.6%	0.02 [−0.34, 0.38]
**Subtotal (95% CI)**			**82**			**34**	**40.0%**	**−0.12 [−0.44, 0.19]**
Heterogeneity: $Tau^2 = 0.01$; $Chi^2 = 1.35$, df = 1 (P = 0.24); $I^2 = 26\%$								
Test for overall effect: Z = 0.78 (P = 0.43)								
**Total (95% CI)**			**212**			**84**	**100.0%**	**−0.25 [−0.60, 0.09]**
Heterogeneity: $Tau^2 = 0.12$; $Chi^2 = 16.68$, df = 4 (P = 0.002); $I^2 = 76\%$								
Test for overall effect: Z = 1.44 (P = 0.15)								
Test for subgroup differences: $Chi^2 = 0.38$, df = 1 (P = 0.54), $I^2 = 0\%$								

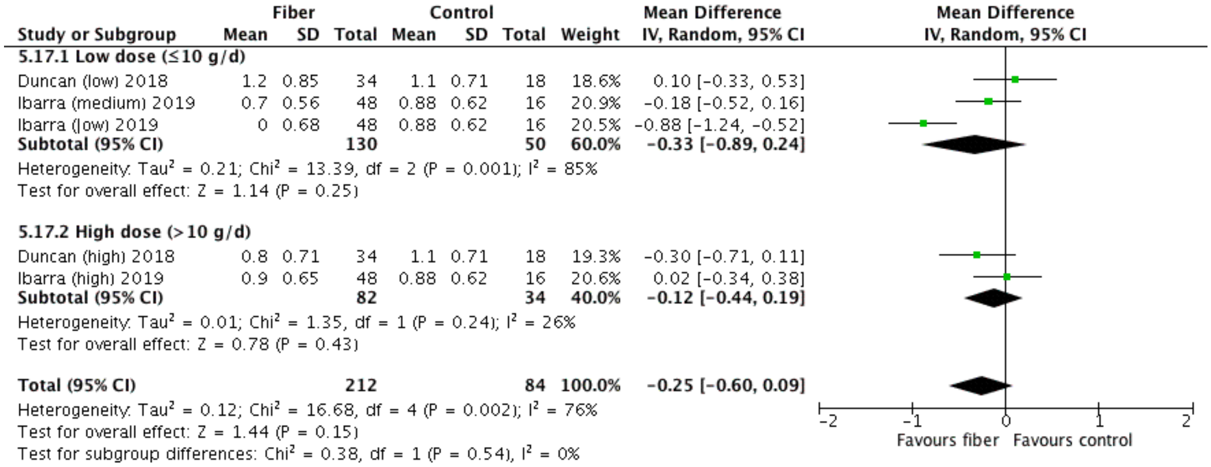

Supplementary Figure 21B Forest plot of subgroup analysis based on dose administered for PAC-QoL (worries and concerns) in randomized controlled trials comparing fiber with control in adults with chronic constipation (n=296). Values were calculated as mean difference (95% CIs) using a random-effects model. Abbreviations: IV, inverse variance; PAC-QoL, Patient Assessment of Constipation Quality of Life.

	Fiber			Control				Mean Difference
Study or Subgroup	Mean	SD	Total	Mean	SD	Total	Weight	IV, Random, 95% CI
**4.14.1 Duration of treatment < 4 weeks**								
Ibarra (high) 2019	0.9	0.65	48	0.88	0.62	16	20.6%	0.02 [−0.34, 0.38]
Ibarra (medium) 2019	0.7	0.56	48	0.88	0.62	16	20.9%	−0.18 [−0.52, 0.16]
Ibarra (low) 2019	0	0.68	48	0.88	0.62	16	20.5%	−0.88 [−1.24, −0.52]
**Subtotal (95% CI)**			**144**			**48**	**62.1%**	**−0.35 [−0.87, 0.18]**
Heterogeneity: Tau² = 0.19; Chi² = 13.45, df = 2 (P = 0.001); I² = 85%								
Test for overall effect: Z = 1.28 (P = 0.20)								
**4.14.2 Duration of treatment ≥ 4 weeks**								
Duncan (high) 2018	0.8	0.71	34	1.1	0.71	18	19.3%	−0.30 [−0.71, 0.11]
Duncan (low) 2018	1.2	0.85	34	1.1	0.71	18	18.6%	0.10 [−0.33, 0.53]
**Subtotal (95% CI)**			**68**			**36**	**37.9%**	**−0.11 [−0.50, 0.28]**
Heterogeneity: Tau² = 0.03; Chi² = 1.74, df = 1 (P = 0.19); I² = 42%								
Test for overall effect: Z = 0.54 (P = 0.59)								
**Total (95% CI)**			**212**			**84**	**100.0%**	**−0.25 [−0.60, 0.09]**
Heterogeneity: Tau² = 0.12; Chi² = 16.68, df = 4 (P = 0.002); I² = 76%								
Test for overall effect: Z = 1.44 (P = 0.15)								
Test for subgroup differences: Chi² = 0.50, df = 1 (P = 0.48), I² = 0%								

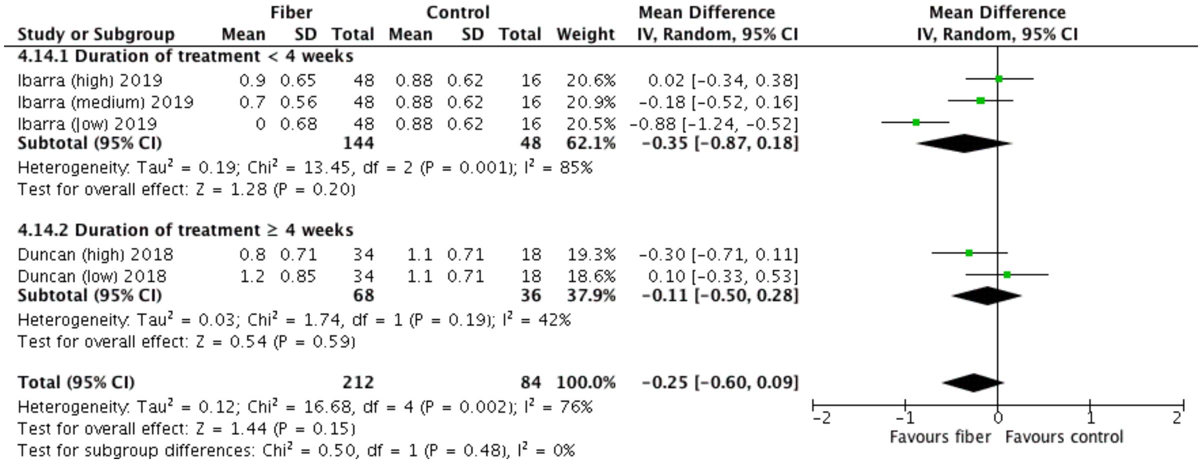

Supplementary Figure 21C Forest plot of subgroup analysis based on treatment duration for PAC-QoL (worries and concerns) in randomized controlled trials comparing fiber with control in adults with chronic constipation (n=296). Values were calculated as mean difference (95% CIs) using a random-effects model. Abbreviations: IV, inverse variance; PAC-QoL, Patient Assessment of Constipation Quality of Life.

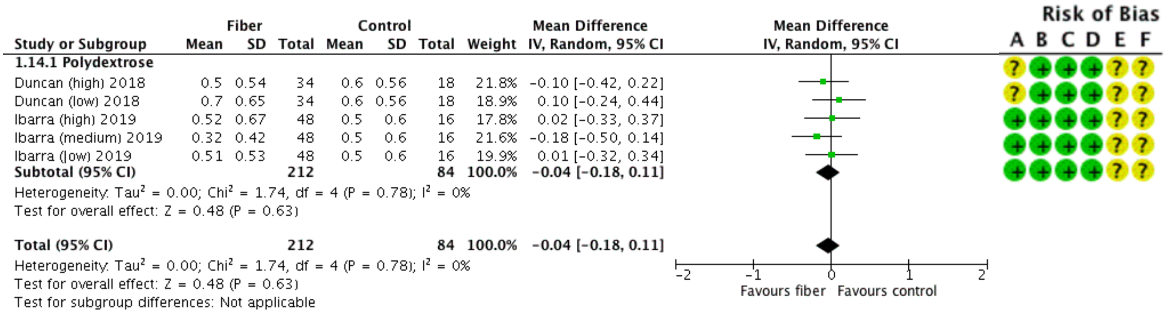

Supplementary Figure 22A Forest plot of PAC-QoL (psychosocial discomfort) in randomized controlled trials comparing fiber with control in adults with chronic constipation (n=296). Values were calculated as mean difference (95% CIs) using a random-effects model. Risk of bias key: A=bias arising from the randomization process; B=bias arising from deviations from intended interventions; C=bias due to missing outcome data; D=bias in measurement of the outcome; E=bias in selection of the reported result; F=overall risk of bias. Abbreviations: IV, inverse variance; PAC-QoL, Patient Assessment of Constipation Quality of Life.

Study or Subgroup	Fiber Mean	Fiber SD	Fiber Total	Control Mean	Control SD	Control Total	Weight	Mean Difference IV, Random, 95% CI
**5.18.2 Low dose (≤10 g/d)**								
Duncan (low) 2018	0.7	0.65	34	0.6	0.56	18	18.9%	0.10 [−0.24, 0.44]
Ibarra (medium) 2019	0.32	0.42	48	0.5	0.6	16	21.6%	−0.18 [−0.50, 0.14]
Ibarra (low) 2019	0.51	0.53	48	0.5	0.6	16	19.9%	0.01 [−0.32, 0.34]
**Subtotal (95% CI)**			**130**			**50**	**60.5%**	**−0.03 [−0.22, 0.16]**
Heterogeneity: Tau² = 0.00; Chi² = 1.48, df = 2 (P = 0.48); I² = 0%								
Test for overall effect: Z = 0.31 (P = 0.76)								
**5.18.3 High dose (>10 g/d)**								
Duncan (high) 2018	0.5	0.54	34	0.6	0.56	18	21.8%	−0.10 [−0.42, 0.22]
Ibarra (high) 2019	0.52	0.67	48	0.5	0.6	16	17.8%	0.02 [−0.33, 0.37]
**Subtotal (95% CI)**			**82**			**34**	**39.5%**	**−0.05 [−0.28, 0.19]**
Heterogeneity: Tau² = 0.00; Chi² = 0.25, df = 1 (P = 0.62); I² = 0%								
Test for overall effect: Z = 0.39 (P = 0.70)								
**Total (95% CI)**			**212**			**84**	**100.0%**	**−0.04 [−0.18, 0.11]**
Heterogeneity: Tau² = 0.00; Chi² = 1.74, df = 4 (P = 0.78); I² = 0%								
Test for overall effect: Z = 0.48 (P = 0.63)								
Test for subgroup differences: Chi² = 0.01, df = 1 (P = 0.92), I² = 0%								

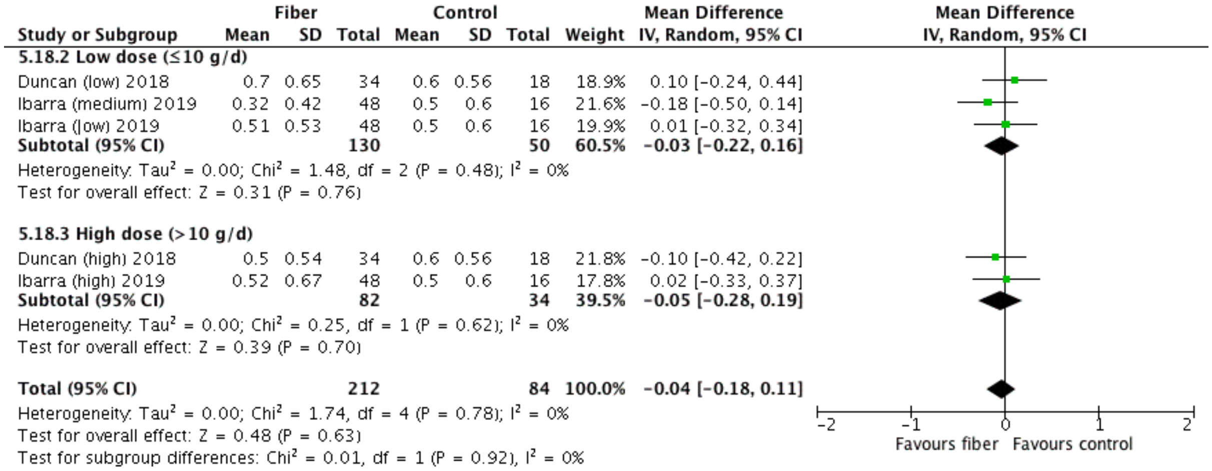

Supplementary Figure 22B Forest plot of subgroup analysis based on dose administered for PAC-QoL (psychosocial discomfort) in randomized controlled trials comparing fiber with control in adults with chronic constipation (n=296). Values were calculated as mean difference (95% CIs) using a random-effects model. Abbreviations: IV, inverse variance; PAC-QoL, Patient Assessment of Constipation Quality of Life.

Study or Subgroup	Fiber Mean	Fiber SD	Fiber Total	Control Mean	Control SD	Control Total	Weight	Mean Difference IV, Random, 95% CI
**4.15.1 Duration of treatment < 4 weeks**								
Ibarra (high) 2019	0.52	0.67	48	0.5	0.6	16	17.8%	0.02 [−0.33, 0.37]
Ibarra (medium) 2019	0.32	0.42	48	0.5	0.6	16	21.6%	−0.18 [−0.50, 0.14]
Ibarra (low) 2019	0.51	0.53	48	0.5	0.6	16	19.9%	0.01 [−0.32, 0.34]
**Subtotal (95% CI)**			**144**			**48**	**59.3%**	**−0.06 [−0.25, 0.14]**
Heterogeneity: Tau² = 0.00; Chi² = 0.92, df = 2 (P = 0.63); I² = 0%								
Test for overall effect: Z = 0.58 (P = 0.56)								
**4.15.2 Duration of treatment ≥ 4 weeks**								
Duncan (high) 2018	0.5	0.54	34	0.6	0.56	18	21.8%	−0.10 [−0.42, 0.22]
Duncan (low) 2018	0.7	0.65	34	0.6	0.56	18	18.9%	0.10 [−0.24, 0.44]
**Subtotal (95% CI)**			**68**			**36**	**40.7%**	**−0.01 [−0.24, 0.22]**
Heterogeneity: Tau² = 0.00; Chi² = 0.72, df = 1 (P = 0.40); I² = 0%								
Test for overall effect: Z = 0.06 (P = 0.95)								
**Total (95% CI)**			**212**			**84**	**100.0%**	**−0.04 [−0.18, 0.11]**
Heterogeneity: Tau² = 0.00; Chi² = 1.74, df = 4 (P = 0.78); I² = 0%								
Test for overall effect: Z = 0.48 (P = 0.63)								
Test for subgroup differences: Chi² = 0.10, df = 1 (P = 0.75), I² = 0%								

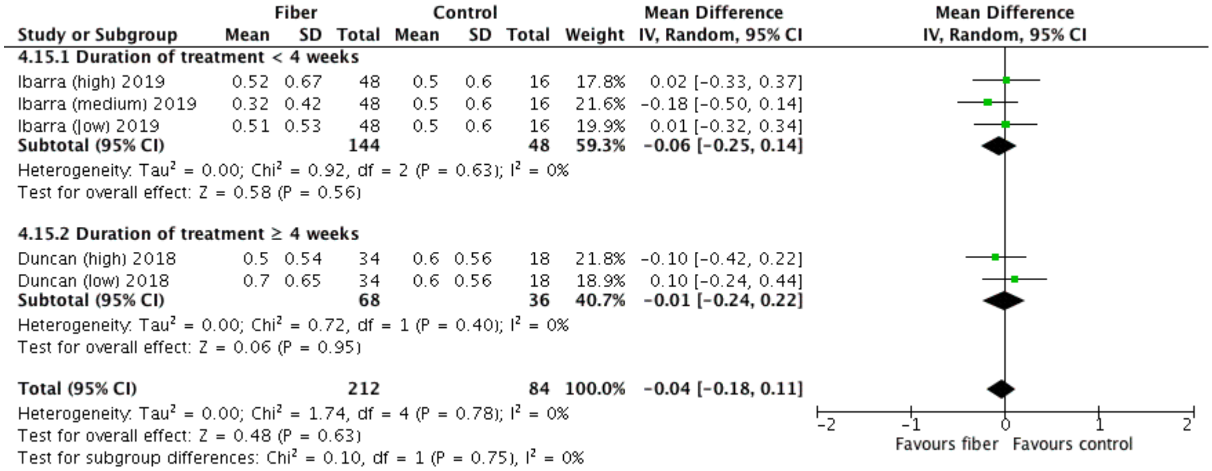

Supplementary Figure 22C Forest plot of subgroup analysis based on duration of treatment for PAC-QoL (psychosocial discomfort) in randomized controlled trials comparing fiber with control in adults with chronic constipation (n=296). Values were calculated as mean difference (95% CIs) using a random-effects model. Abbreviations: IV, inverse variance; PAC-QoL, Patient Assessment of Constipation Quality of Life.

Section 4. Funnel plots to assess publication bias

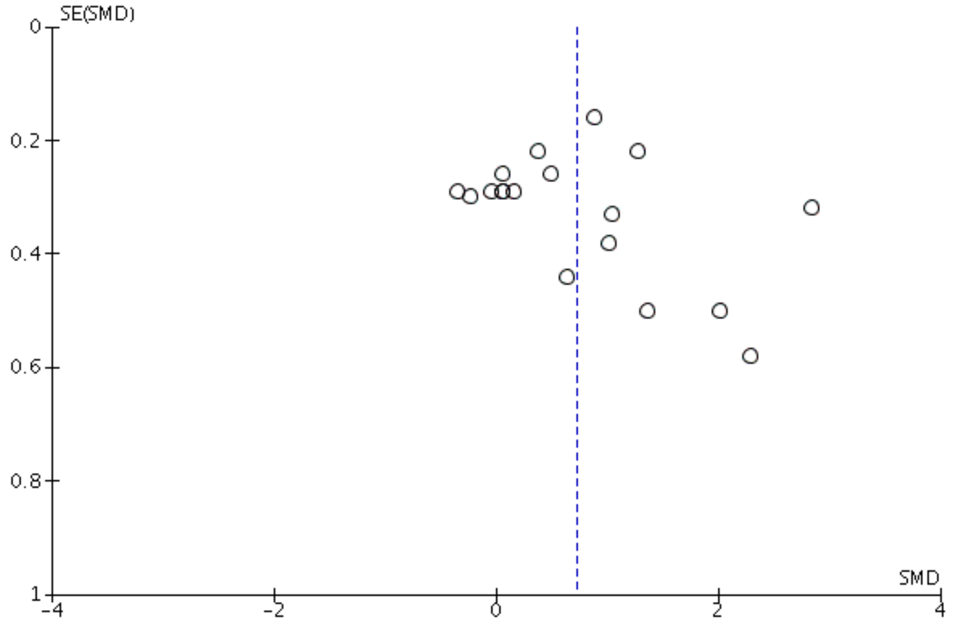

Supplementary Figure 23 Funnel plot of stool frequency outcome in randomized controlled trials comparing fiber supplementation to control in adults with chronic constipation (n=1040). Values were calculated as standardized mean difference (95% CIs) using a random-effects model. Abbreviation: SMD = standardized mean difference.

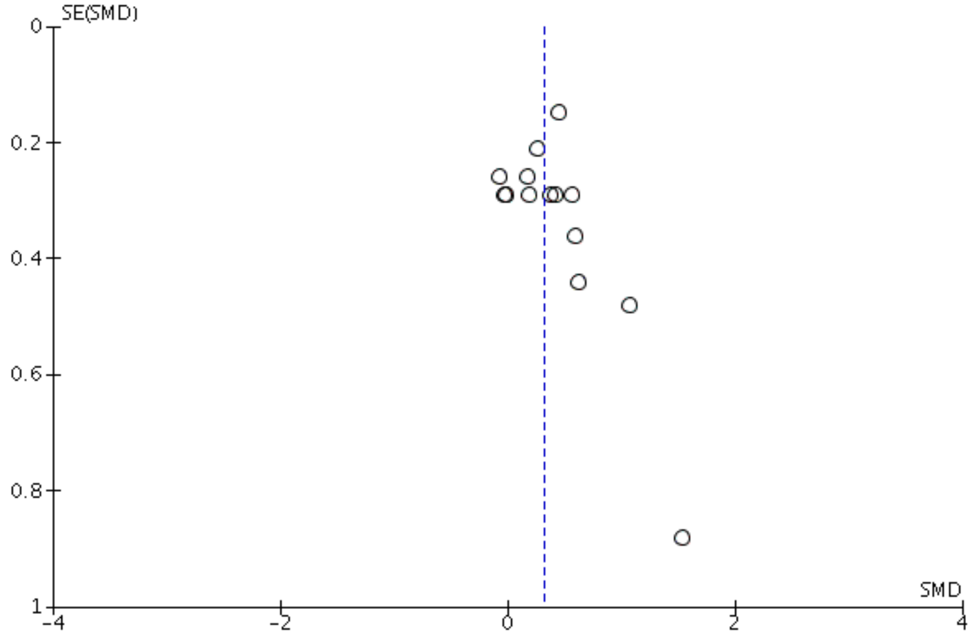

Supplementary Figure 24 Funnel plot of stool consistency outcome in randomized controlled trials comparing fiber supplementation to control in adults with chronic constipation (n=918). Values were calculated as standardized mean difference (95% CIs) using a random-effects model. Abbreviation: SMD = standardized mean difference.

References

1. Ito D, Yamamoto Y, Maekita T, Yamagishi N, Kawashima S, Yoshikawa T, Tanioka K, Yoshida T, Iguchi M, Kunitatsu K et al. Do synbiotics really enhance beneficial synbiotics effect on defecation symptoms in healthy adults?: Randomized, double-blind, placebo-controlled trial. Medicine. 2022;101(8):e28858.
2. Müller M, Hermes GDA, Emanuel E C, Holst JJ, Zoetendal EG, Smidt H, Troost F, Schaap FG, Damink SO, Jocken JWE, Lenaerts K, Masclee AAM, Blaak EE. Effect of wheat bran derived prebiotic supplementation on gastrointestinal transit, gut microbiota, and metabolic health: a randomized controlled trial in healthy adults with a slow gut transit. Gut Microbes. 2020;12(1):1704141.
3. Alyousif Z, Mendoza DR, Auger J, De Carvalho V, Amos S, Sims C, Dahl WJ.Gastrointestinal Tolerance and Microbiome Response to Snacks Fortified with Pea Hull Fiber: A Randomized Trial in Older Adults. Curr Dev Nutr. 2020;4(2):nzaa005.
4. Zhang M, Wang B, Zhou C, Huang X. Application of Probiotics Combined With Soluble Dietary Fiber in Middle-aged and Elderly Patients With Chronic Constipation. [Chinese]. Chinese Journal of Gastroenterology. 2019;24(2):95-8.
5. Watson AW, Houghton D, Avery PJ, Stewart C, Vaughan EE, Meyer PD, de Bos Kuil MJJ, Weijs PJM, Brandt K. Changes in stool frequency following chicory inulin consumption, and effects on stool consistency, quality of life and composition of gut microbiota. Food Hydrocoll. 2019;96:688-98.
6. Machado AM, da Silva NBM, Chaves JBP, Alfenas RDG. Consumption of yacon flour improves body composition and intestinal function in overweight adults: A randomized, double-blind, placebo-controlled clinical trial. Clinical Nutrition Espen. 2019;29:22-9.
7. Huaman JW, Mego M, Bendezú A, Monrroy H, Samino S, Accarino A, Saperas E, Azpiroz F. Correction of Dyssynergic Defecation, but Not Fiber Supplementation, Reduces Symptoms of Functional Dyspepsia in Patients With Constipation in a Randomized Trial. Clin Gastroenterol Hepatol. 2020;18(11):2463-2470.e1.
8. Hara K, Wada T, Kaneko T. Effect of Fuji FF (Inulin) containing green tea on balance of gut microbiota and bowel habit-a randomized, double-blind, placebo-controlled, cross-over trial-. [Japanese]. Japanese Pharmacology and Therapeutics. 2019;47(3):479-83.
9. Gokan N, Hoshino T, Takara T. Improvement of the intestinal environment by konjac processed food -a randomized, double-blind, placebo-controlled, parallel-group comparison trial. [Japanese]. Japanese Pharmacology and Therapeutics. 2019;47(9):1445-54.

10. Chu JR, Kang SY, Kim SE, Lee SJ, Lee YC, Sung MK. Prebiotic UG1601 mitigates constipation-related events in association with gut microbiota: A randomized placebo-controlled intervention study. World Journal of Gastroenterology. 2019;25(40):6129-44.
11. Cheng J, Tennilä J, Stenman L, Ibarra A, Kumar M, Gupta KK, Sharma SS, Sen D, Garg S, Penurkar M, Kumar S, Ouwehand AC. Influence of Lactitol and Psyllium on Bowel Function in Constipated Indian Volunteers: A Randomized, Controlled Trial. Nutrients. 2019;11(5):1130.
12. Anzawa D, Mawatari T, Tanaka Y, Yamamoto M, Genda T, Takahashi S, Nishijima T, Kamasaka H, Suzuki S, Kuriki T.. Effects of synbiotics containing Bifidobacterium animalis subsp. lactis GCL2505 and inulin on intestinal bifidobacteria: A randomized, placebo-controlled, crossover study. Food Sci Nutr. 2019;7(5):1828-1837.
13. Hoshino T, Yamashita SI, Suzuki N, Takara T. Effects of food containing resistant dextrin and galactooligosaccharides on the intestinal environment in healthy Japanese adults-A randomized, double-blind, placebo-controlled, parallel-group trial. [Japanese]. Japanese Pharmacology and Therapeutics. 2018;46(10):1707-22.
14. Vandeputte D, Falony G, Vieira-Silva S, Wang J, Sailer M, Theis S, Verbeke K, Raes J. Prebiotic inulin-type fructans induce specific changes in the human gut microbiota. Gut. 2017;66(11):1968-74.
15. Nishimura A, Kitazono E, Imose K, Urita Y, Matsui T. Effect of functional barley BARLEYmax (Tantangara) on intestinal regulation: A double-blind, randomized, placebo-controlled parallel group comparison clinical study. [Japanese]. Japanese Pharmacology and Therapeutics. 2017;45(6):1047-55.
16. Kinoshita K, Horikawa A, Kunishige K, Koikeda T. Evaluation of bowel movement improvement by ingestion of oligosaccharide powder: -a randomized, double-blind, placebo-controlled, crossover, comparative study-. [Japanese]. Japanese Pharmacology and Therapeutics. 2017;45(5):773-80.
17. Aoe S, Nakazawa Y, Ohmisya S. Combined effect of a probiotic (Bifidobacterium) and a prebiotic (polydextrose) on the fecal microbiota and stool parameters of healthy young women. Integrative Molecular Medicine. 2017;4(5):1-5.
18. Ishida Y, Sadakiyo T, Watanabe H, Mitsuzumi H, Ushio S, Takehara I, Takano K. The effect of a beverage containing isomaltodextrin on bowel movements in healthy subjects -A randomized, double-blind, placebo-controlled, crossover study. Japanese Pharmacology and Therapeutics. 2017;45(4):609-16.
19. Hamaguchi N, Hirai H, Kondo S, Umemura S, Kimura T. Effect of resistant glucan on bowel movements in healthy subjects: -A randomized, double-blind, placebo-controlled, parallel-group trial. Japanese Pharmacology and Therapeutics. 2017;45(12):1935-45.

20. Gomez Castrejon KL, Campos Vega R. Effect of antioxidant dietary fiber from spent coffee (coffea arabica L.) grounds on gastrointestinal health of adults. Annals of Nutrition and Metabolism. 2017;71 (Supplement 2):1204-5.
21. Buddington RK, Kapadia C, Neumer F, Theis S. Oligofructose Provides Laxation for Irregularity Associated with Low Fiber Intake. Nutrients. 2017;9(12):18.
22. Nakazeko T, Tanabe S, Matsuo S, Sakano K, Hayashi M, Ebihara S. Safety evaluation of long-term or excessive intake of instant noodles (non-fried soba) containing highly cross-linked phosphate starch. Japanese pharmacology and therapeutics. 2016;44(5):713-24.
23. Major GA, Murray K, Nowak A, Hoad C, Silos-Santiago A, Kurtz CB, Johnston J, Marciana L, Gowland P, Spiller RC. MRI shows increased water and gas in the bowel of constipated patients after psyllium. Gastroenterol. 2016;150(4):S531.
24. Hamaguchi N, Hirai H, Bito H, Ogawa K. Effects of Resistant Glucan Mixture on Bowel Movement in Female Volunteers. Journal of Nutritional Science & Vitaminology. 2016;62(1):62-6.
25. Hoffmann Sardá FA, Giuntini EB, Gomez MLPA, Lui MGY, Negrini JAE, Tadini CC, Lajolo FM, Menezes EW. Impact of resistant starch from unripe banana flour on hunger, satiety, and glucose homeostasis in healthy volunteers. J Funct Foods. 2016; 24:63–74.
26. Gabrielli F, Macchini D, Guttadauro A, Frassani S, Bertolini A, Giussani C, DE Simone M, Cioffi U. Psyllium fiber vs. placebo in early treatment after STARR for obstructed defecation: a randomized double-blind clinical trial. Minerva Chir. 2016;71(2):98-105.
27. Abellán Ruiz MS, Barnuevo Espinosa MD, Contreras Fernández CJ, Luque Rubia AJ, Sánchez Ayllón F, Aldeguer García M, García Santamaría C, López Román FJ. Digestion-resistant maltodextrin effects on colonic transit time and stool weight: a randomized controlled clinical study. Eur Journal of Nutr. 2016;55(8):2389-97.
28. De Souza Lima Sant Anna M, Rodrigues VC, Araujo TF, De Oliveira TT, Do Carmo Gouveia Peluzio M, De Luces Fortes Ferreira CL. Yacon-Based Product in the Modulation of Intestinal Constipation. Journal of Medicinal Food. 2015;18(9):980-6.
29. Koeda T, Hara K, Wada T, Morita T, Arai E. Effect of inulin containing rice bread on bowel habit in Japanese young healthy adult. [Japanese]. Japanese Pharmacology and Therapeutics. 2015;43(12):1731-7.
30. Kishimoto Y, Tachibe M, Kitagawa M, Nishibata T, Shioya N, Sugimura H. Effect of highly cross-linked starch on defecation in adult female subjects with constipation tendency. [Japanese]. Japanese Pharmacology and Therapeutics. 2014;42(9):655-9.

31. Scheid MMA, Genaro PS, Moreno YMF, Pastore GM. Freeze-dried powdered yacon: effects of FOS on serum glucose, lipids and intestinal transit in the elderly. European Journal of Nutrition. 2014;53(7):1457-64.
32. Polymeros D, Beintaris I, Gaglia A, Karamanolis G, Papanikolaou IS, Dimitriadis G, Triantafyllou K. Partially hydrolyzed guar gum accelerates colonic transit time and improves symptoms in adults with chronic constipation. Dig Dis Sci. 2014;59(9):2207-14.
33. Lawton CL, Walton J, Hoyland A, Howarth E, Allan P, Chesters D, Dye L. Short term (14 days) consumption of insoluble wheat bran fibre-containing breakfast cereals improves subjective digestive feelings, general wellbeing and bowel function in a dose dependent manner. Nutrients. 2013;5(4):1436-55.
34. Granata M, Brandi G, Borsari A, Gasbarri R, Di Gioia D. Synbiotic yogurt consumption by healthy adults and the elderly: the fate of bifidobacteria and LGG probiotic strain. International Journal of Food Sciences and Nutrition. 2013;64(2):162-8.
35. Asano Y, Katayama M. Effects of soybean soluble polysaccharide derived from "okara" on japanese women with constipation. Annals of Nutrition and Metabolism. 2013;1):1635.
36. François IE, Lescroart O, Veraverbeke WS, Marzorati M, Possemiers S, Evenepoel P, Hamer H, Houben E, Windey K, Welling GW. Effects of a wheat bran extract containing arabinoxylan oligosaccharides on gastrointestinal health parameters in healthy adult human volunteers: a double-blind, randomised, placebo-controlled, cross-over trial. Br J Nutr. 2012;108(12):2229-42.
37. Yen CH, Kuo YW, Tseng YH, Lee MC, Chen HL. Beneficial effects of fructo-oligosaccharides supplementation on fecal bifidobacteria and index of peroxidation status in constipated nursing-home residents-A placebo-controlled, diet-controlled trial. Nutrition. 2011;27(3):323-8.
38. Tomono Y, Yamamoto T, Yamaguchi H. Effect of synthesized inulin on bowel habit and fecal microflora in healthy adults with low fecal frequency. Japanese Pharmacology and Therapeutics. 2010;38(11):1031-40.
39. Surakka A, Kajander K, Rajilic-Stojanovic M, Karjalainen H, Hatakka K, Vapaatalo H, Zoetendal EG, Vos WM de, Korpela R, Tynkkynen S. Yoghurt containing galactooligosaccharides facilitates defecation among elderly subjects and selectively increases the number of Bifidobacteria. International Journal of Probiotics and Prebiotics. 2009;4(1):65-74.
40. Pilipenko VI, Burlyaeva EA, Shakhovskaya AK, Isakov VA. Efficacy of using inulin fortified fermented milk products in patients with functional constipation. [Russian]. Voprosy Pitaniia. 2009;78(3):56-61.
41. Park EJ, Jhon DY. Effects of bamboo shoot consumption on lipid profiles and bowel function in healthy young women. Nutrition. 2009;25(7-8):723-8.

42. Liu BH, Yang XQ, Ren DL, Ding YJ, Zhao K, Qian Q, Yang GG, Wang D. Efficacy of cellulose on functional constipation. [Chinese]. Zhonghua wei chang wai ke za zhi = Chinese journal of gastrointestinal surgery. 2009;12(2):182-4.
43. Hengst C, Ptok S, Roessler A, Fechner A, Jahreis G. Effects of polydextrose supplementation on different faecal parameters in healthy volunteers. International Journal of Food Sciences & Nutrition. 2009;60 Suppl 5:96-105.
44. Paineau D, Payen F, Panserieu S, Coulombier G, Sobaszek A, Lartigau I, Brabet M, Galmiche JP, Tripodi D, Sacher-Huvelin S. The effects of regular consumption of short-chain fructo-oligosaccharides on digestive comfort of subjects with minor functional bowel disorders. British Journal of Nutrition. 2008;99(2):311-8.
45. Chen HL, Cheng HC, Wu WT, Liu YJ, Liu SY. Supplementation of konjac glucomannan into a low-fiber Chinese diet promoted bowel movement and improved colonic ecology in constipated adults: a placebo-controlled, diet-controlled trial. Journal of the American College of Nutrition. 2008;27(1):102-8.
46. Geyer M, Manrique I, Degen L, Beglinger C. Effect of yacon (Smallanthus sonchifolius) on colonic transit time in healthy volunteers. Digestion. 2008;78(1):30-33
47. Kim JY, Kim OY, Yoo HJ, Kim TI, Kim WH, Yoon YD. Effects of Fiber Supplements on Functional Constipation. Korean journal of nutrition. 2006;39(1):35-43.
48. Hongisto SM, Paajanen L, Saxelin M, Korpela R. A combination of fibre-rich rye bread and yoghurt containing Lactobacillus GG improves bowel function in women with self-reported constipation. Eur J Clin Nutr. 2006;60(3):319-324.
49. Lin SD, Lim PS, Wang HF, Hsiao CC. Effects of isomaltooligosaccharide chiffon cake on serum biochemical parameters, constipation, and fecal putrefactive metabolites in hyperlipidemic subjects. [Chinese]. Nutritional Sciences Journal. 2005;30(2):108-15.
50. Kim TI, Park SJ, Choi CH, Lee SK, Kim WH. Effect of ear mushroom (Auricularia) on functional constipation. [Korean]. The Korean journal of gastroenterology = Taehan Sohwagi Hakhoe chi. 2004;44(1):34-41.
51. Den Hond E, Geypens B, Ghoos Y. Effect of high performance chicory inulin on constipation. Nutrition Research. 2000;20(5):731-6.
52. Teuri U, Korpela R. Galacto-oligosaccharides relieve constipation in elderly people. Annals of Nutrition & Metabolism. 1998;42(6):319-27.
53. Sasaki K. Efficacy of Dietary Fiber in Young Women with Self-Perceived Constipation. Rinsho to kenkyu (the japanese journal of clinical and experimental medicine). 1998;75(9):1990-4.
54. Patrick PG, Gohman SM, Marx SC, DeLegge MH, Greenberg NA. Effect of supplements of partially hydrolyzed guar gum on the occurrence of

constipation and use of laxative agents. Journal of the American Dietetic Association. 1998;98(8):912-4.
55. Neal LJ. "Power pudding": natural laxative therapy for the elderly who are homebound. Home Healthcare Nurse. 1995;13(3):66-71.
56. Marsicano LJ, Berrizbeitia ML, Mondelo A. Use of the Glucomannan dietery fiber in the modification of the intestinal habit. GEN. 1995;49(1):7-14.
57. Gibson GR, Beatty ER, Wang X, Cummings JH. Selective stimulation of bifidobacteria in the human colon by oligofructose and inulin. Gastroenterology. 1995;108(4):975-82.
58. Cheskin LJ, Kamal N, Crowell MD, Schuster MM, Whitehead WE. Mechanisms of constipation in older persons and effects of fiber compared with placebo. Journal of the American Geriatrics Society. 1995;43(6):666-9.
59. Bobbio F, Giussani E, Zaccala G. Comparative study of an association of lactulose and glucomannan (Dimalosio) vs. lactulose in the treatment of chronic constipation. [Italian]. Rassegna Internazionale di Clinica e Terapia. 1995;75(7):313-22.
60. Takahashi H, Wako N, Okubo T, Ishihara N, Yamanaka J, Yamamoto T. Influence of partially hydrolyzed guar gum on constipation in women. Journal of Nutritional Science & Vitaminology. 1994;40(3):251-9.
61. Odes HS, Lazovski H, Stern I, Madar Z. [Fiber-enriched cereal for constipation and for hypercholesterolemia]. Harefuah. 1993;125(5-6):136-8.
62. Quaade F, Vrist E, Astrup A. Dietary fiber added to a very-low caloric diet reduces hunger and alleviates constipation. [Danish]. Ugeskrift for laeger. 1990;152(2):95-8.
63. Astrup A, Vrist E, Quaade F. Dietary fibre added to very low calorie diet reduces hunger and alleviates constipation. International Journal of Obesity. 1990;14(2):105-12.
64. Prior A, Whorwell PJ. Double blind study of ispaghula in irritable bowel syndrome. Gut. 1987;28(11):1510-3.
65. Pergola F. [Evaluation of a new therapy (beidellian montmorillonite--guar gum) in the treatment of constipation]. Annales de Gastroenterologie et d Hepatologie. 1987;23(1):43-6.
66. Borgia M, Brancato V, Borgia R. [Controlled study on the effects of 2 different therapeutic approaches in the treatment of chronic constipation]. Clinica Terapeutica. 1986;118(3):165-70.
67. Pulpeiro A, Rodríguez French A, Zagalsky D, Forte IN. Tratamiento de la constipación con una fibra dietética / Treatment of constipation with a dietetic fiber. Medicina. 1985;45:513-6.
68. Sculati O, Giampiccoli G. Clinical trial of a new preparation with a high concentration of dietary fiber (fibraform). Current Therapeutic Research - Clinical and Experimental. 1984;36(2):261-6.
69. Pulpeiro A, Rodriguez A, Forti IN. Treatment of chronic constipation with a dietary fiber: natuvit. Medecine & chirurgie digestives. 1984;13(4):315-8.

70. Corinaldesi R, Stanghellini V, Bocci G. Dietary fibers and intestinal transit times. Current Therapeutic Research - Clinical and Experimental. 1982;31(2):173-80.
71. Bjorneklett A, Fausa O, Lovik A, Ritland S, Gjone E. Fibre tablets as effective alternative to wheat bran in chronic constipation. Acta medica scandinavica supplement. 1978;621:45.
72. Schlagheck TG, Doane JC, Borden LC, Daggy BP, Morel JG, Pucke JE, Allgood LD. Evaluation of an appropriate placebo for laxation studies with psyllium in idiopathic constipation patients. Gastroenterology. 1996;110(4):A754-A.
73. Danese, S. (Feb 2012 – Jan 2016). ClinicalTrials.gov. Dose-Responsive Effect of Polydextrose on Whole Gut Transit Time (PDX-Transit). Identifier: NCT01540669. Available from: https://clinicaltrials.gov/ct2/show/NCT01540669.
74. Benamouzig, R. (May 2013 – Oct 2018). ClinicalTrials.gov. Effects of scFOS on Stool Frequency in People With Functionnal Constipation. Identifier: NCT01847950. Available from: https://clinicaltrials.gov/ct2/show/NCT01847950
75. Gendre, D. (Oct 2018) ClinicalTrials.gov. Effect of scFOS on Increase in Stool Frequency in Constipated People (CONSYST). Identifier: NCT03707002. Available from: https://clinicaltrials.gov/ct2/show/NCT03707002
76. Danone Research. (June 2015 - Aug 2017). ClinicalTrials.gov. Effect of a Fermented Milk Product With Probiotics Alone or in Combination With Fibers on Constipation Symptoms. 2017. Identifier: NCT02461485. Available from: https://clinicaltrials.gov/ct2/show/NCT02461485.
77. Ning, L. ClinicalTrials.gov. (Nov 2014 – Feb 2016). Evaluating the Synergism of Soluble Dietary Fiber With Fecal Microbiota Transplantation in Slow Transit Constipation. Identifier: NCT02291354. Available from: https://clinicaltrials.gov/show/NCT02291354.
